# Radical cyanomethylation *via* vinyl azide cascade-fragmentation†
†Dedicated to Professor Richard J. K. Taylor on the occasion of his 70^th^ birthday.
‡
‡Electronic supplementary information (ESI) available: Experimental protocols, cyclic voltammetry, quantum yield measurements and spectral data. See DOI: 10.1039/c9sc01370a


**DOI:** 10.1039/c9sc01370a

**Published:** 2019-05-07

**Authors:** James R. Donald, Sophie L. Berrell

**Affiliations:** a Department of Chemistry , University of York , Heslington , York , YO10 5DD , UK . Email: james.donald@york.ac.uk

## Abstract

Herein, a novel methodology for radical cyanomethylation is described. The process is initiated by radical addition to the vinyl azide reagent 3-azido-2-methylbut-3-en-2-ol which triggers a cascade-fragmentation mechanism driven by the loss of dinitrogen and the stabilised 2-hydroxypropyl radical, ultimately effecting cyanomethylation. Cyanomethyl groups can be efficiently introduced into a range of substrates *via* trapping of α-carbonyl, heterobenzylic, alkyl, sulfonyl and aryl radicals, generated from a variety of functional groups under both photoredox catalysis and non-catalytic conditions. The value of this approach is exemplified by the late-stage cyanomethylation of pharmaceuticals.

## Abstract 2

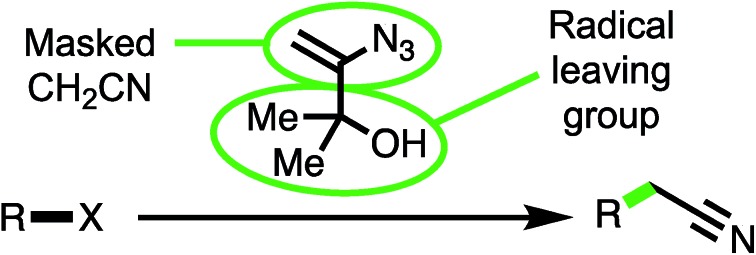
The cyanomethylation of radicals with a vinyl azide reagent is described, *via* a cascade-fragmentation mechanism.

## Introduction

The recent renaissance of synthetic organic radical chemistry has seen the development of several approaches for the introduction of nitrile functionality into molecules through the trapping of radical intermediates with a variety of closed-shell reagents. These are valuable transformations given the importance of nitriles, which are present within the structures of a number of pharmaceuticals and bioactive natural products.^1^ Nitriles are also widely used as directing groups in C–H activation chemistry^2^ and as versatile synthetic intermediates, particularly as precursors to heterocycles^3^ and functionality at the carboxylic acid oxidation level.^4^

**Scheme 1:**
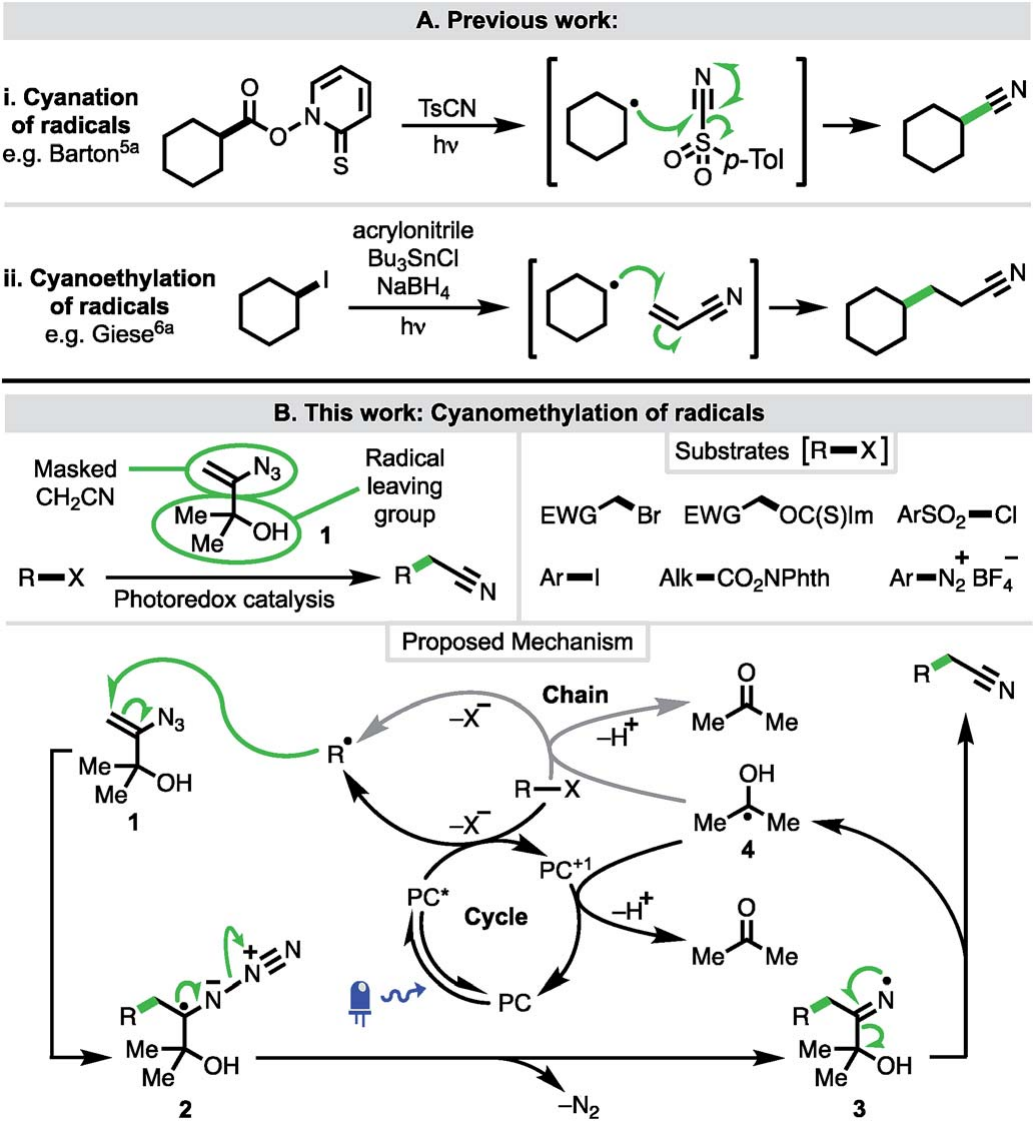
The cyanation, cyanoethylation and cyanomethylation of radicals.

Modern methods to intercept radicals and directly install cyano groups use a range of cyanating reagents and build upon classical studies by Barton using tosyl cyanide and the eponymous Barton esters (Scheme 1A(i)).5*a* Alkyl examples include photoredox-catalysed deboronative cyanation5*b* and α-heteroatom C–H cyanation with tosyl cyanide,5*c* and decarboxylative cyanation with the iodane cyanobenziodoxolone (CBX).5*d* Enantioselective variants have achieved cyanation at benzylic positions *via* C–H abstraction under asymmetric copper catalysis5*e* and decarboxylation of *N*-hydroxyphthalimido esters under cooperative photoredox-asymmetric Cu catalysis;5*f* both methods using TMSCN as the cyanide source. The direct C–H cyanation of arenes has also been performed under photoredox catalysis, using cyanide generated from TMSCN to trap an aryl radical cation.5*g*

The cyanoethylation of radicals exploits the well-established Giese reaction of radical conjugate addition to acrylonitrile (Scheme 1A(ii)).6*a* Notable recent examples feature nucleophilic alkyl and acyl radicals generated from enamines6*f* trifluoroborate salts,6*b**N*-hydroxyphthalimido esters,6b–e and carboxylic acids.6g,h

In contrast to cyanation and cyanoethylation, a method in which radicals can be trapped in a cyanomethylation reaction (*i.e.* a two-carbon homologation process) is not known. At present, radical cyanomethylation can only be achieved *via* the converse approach of adding an electrophilic cyanomethyl radical to electron-rich substrates, limiting both substrate scope and the sites at which cyanomethylation is possible.^7^ Thus, to address this deficiency, we planned to develop a new approach that would enable the facile introduction of useful cyanomethyl groups into a broad range of substrates under mild conditions, such as *via* the use of visible-light driven photoredox catalysis.

To this end, 3-azido-2-methylbut-3-en-2-ol (**1**)^8^ was considered ideally suited to achieve the cyanomethylation of radicals because it encompasses two key design elements: (i) a vinyl azide which can act as a masked cyanomethyl group, and (ii) a dimethylcarbinol as a latent radical leaving group (Scheme 1B). Following radical generation from a substrate *e.g. via* the oxidative quenching of an excited-state photoredox catalyst (PC^*^ → PC^+1^), it was anticipated that reagent **1** would intercept open-shell species to initiate a cascade process through radical addition to the olefin,^9^ affording adduct **2** which would readily expel dinitrogen to produce iminyl radical **3**.^10^ Subsequent fragmentation of iminyl radical **3** through α-C–C bond cleavage and ejection of the stabilised 2-hydroxypropyl radical **4** was envisaged to drive the formation of the nitrile functionality.^11^ Importantly, the low oxidation potential of radical **4** [*E*_Ox_^1/2^ = –0.61 V *vs.* saturated calomel electrode (SCE)]^12^ would potentially make reagent **1** amenable to use both under photoredox catalysis, where radical **4** could readily undergo electron transfer to the oxidised form of a photocatalyst (PC^+^) to close a redox-neutral oxidative quenching cycle, and in other electron transfer processes such as to another molecule of substrate R–X in a chain propagation (see proposed mechanism). Interestingly, azide **1** has previously been utilised in the ionic cyanomethylation of stabilised *p*-quinone methides, promoted by BF_3_·OEt_2_*via* a distinct mechanism.^13^ In this paper, we report the successful implementation of vinyl azide **1** as a new reagent for the direct cyanomethylation of a range of radicals generated from a broad variety of precursors under both photoredox-catalysed and non-photocatalysed radical generation.

**Scheme 2:**
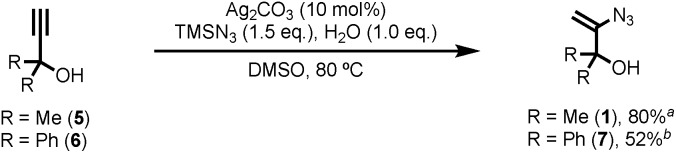
Reagent synthesis. Reaction time ^a^2 h, ^b^6 h.

Known vinyl azide **1** and novel diphenyl analogue **7** were prepared from the corresponding alkynes *via* Bi's Ag(i) catalysed hydroazidation methodology (Scheme 2).^8^ Careful control of the equivalents of water and modification of the work-up and purification procedures facilitated isolation of product **1** in 80% yield on a 60 mmol scale (6 g obtained,^14^ see ESI‡ for details). The cyclic voltammogram of azide **1** exhibited a single reduction process with a peak current at –1.68 V *vs.* SCE. The relatively large magnitude of this value suggests that direct reduction of **1***via* single-electron transfer is unlikely to be competitive with the proposed reaction mechanism.

Reaction development commenced with the evaluation of vinyl azide **1** in the cyanomethylation of 2-bromoacetophenone [*E*_red_^1/2^ = –1.13 V *vs.* SCE]^15^ in the presence of 2,6-lutidine and a range of photocatalysts (1.0 mol%) with strongly reducing photoexcited-states capable of inducing radical formation *via* spin-centre shift. All of the catalysts tested afforded cyanomethylated product **9** in high efficiency (Table 1, entries 1–3, see ESI‡ for full details). Ru(bpy)_3_Cl_2_·6H_2_O was selected on grounds of cost and commercial availability, providing nitrile **9** in 93% yield by ^1^H NMR, and 97% isolated yield on a 1.0 mmol scale. Diphenyl bearing vinyl azide **7** performed with similar efficacy in the radical cyanomethylation process (92% yield) suggesting that a family of related structures might be viable reagents for this transformation. Given that the reaction by-products from reagent **1** are simply nitrogen and acetone, it was preferred over azide **7** which liberates benzophenone, for reasons of atom-economy and purification. When run in CH_2_Cl_2_ or DMF, the reaction proceeded with efficiency comparable to using MeCN as solvent (entries 5 and 6).

**Table 1:** Reaction optimisation^*a*^

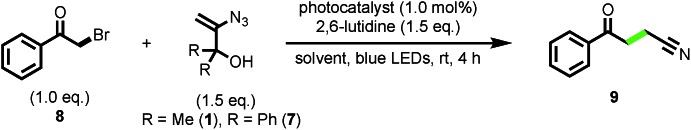				
Entry	Azide	Photocatalyst	Solvent	Yield of **9** (%)
1	**1**	Ru(bpy)_3_Cl_2_·6H_2_O	MeCN	93, 97^*b*^
2	**1**	*fac*-Ir(ppy)_3_	MeCN	97
3	**1**	4CzIPN	MeCN	95
4	**7**	Ru(bpy)_3_Cl_2_·6H_2_O	MeCN	92
5	**1**	Ru(bpy)_3_Cl_2_·6H_2_O	CH_2_Cl_2_	93
6	**1**	Ru(bpy)_3_Cl_2_·6H_2_O	DMF	93
7	**1**	—	MeCN	0
8^*c*^	**1**	Ru(bpy)_3_Cl_2_·6H_2_O	MeCN	0
9^*d*^	**1**	Ru(bpy)_3_Cl_2_·6H_2_O	MeCN	7
10^*e*^	**1**	Ru(bpy)_3_Cl_2_·6H_2_O	MeCN	0

^*a*^Reactions performed on a 0.2 mmol scale. Yields were determined by ^1^H NMR integration against 1,3-benzodioxole as an internal standard.

^*b*^Isolated yield on a 1.0 mmol scale.

^*c*^No light.

^*d*^No 2,6-lutidine.

^*e*^With TEMPO [(2,2,6,6-tetramethylpiperidin-1-yl)oxyl] (2.0 eq.).

Control experiments confirmed that both photocatalyst and light were necessary for product formation, and that the yield was much lower in the absence of base – presumably due to the acid (HBr) promoted decomposition of vinyl azide **1** (entries 7–9). Performing the reaction in the presence of TEMPO (2.0 eq.) completely suppressed the formation of product **9**, and lowered the conversion of bromide **8**, with 89% remaining after 4 h; indicative of a radical mechanism (entry 10). Quantum yield measurements for the reactions with azides **1** and **7** (entries 1 and 4) determined values of *Φ* = 1.8 and *Φ* = 0.6, respectively; suggesting that mechanistic contributions from radical chain processes cannot be ruled out (see ESI‡ for details).^16^

**Scheme 3:**
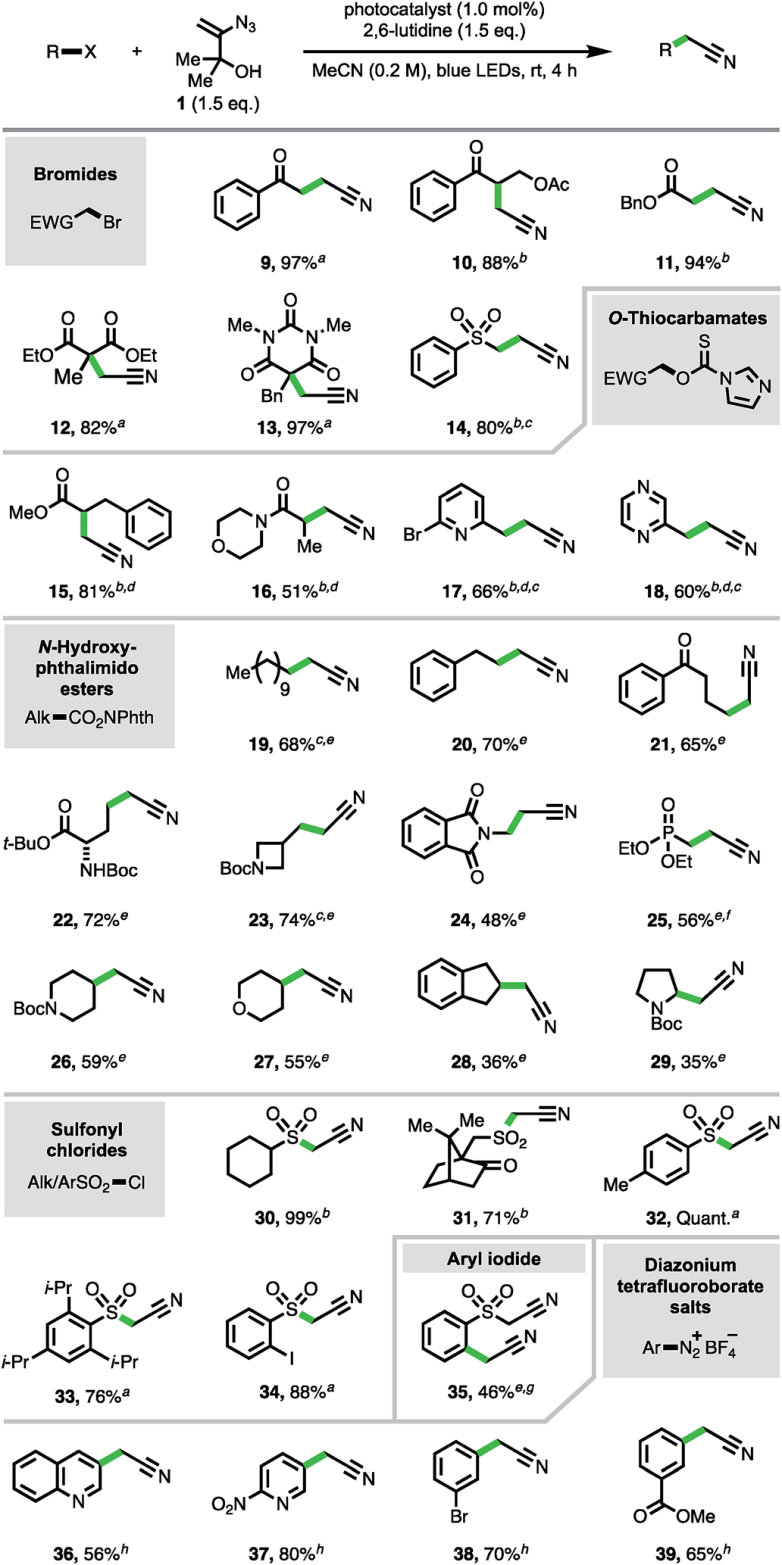
Cyanomethylation of radicals. Reaction conditions: all reactions run on a 1.0 mmol scale; ^a^Ru(bpy)_3_Cl_2_·6H_2_O; ^b^*fac*-Ir(ppy)_3_; ^c^24 h, ^d^no 2,6-lutidine; ^e^*fac*-Ir(5-Fppy)_3_, (+)-sodium l-ascorbate (1.5 eq.); ^f^no (+)-sodium l-ascorbate; ^g^8 h; ^h^no photocatalyst, no LEDs.

The focus turned next to exploration of the nature of the substrates and radical intermediates that could be cyanomethylated with vinyl azide **1**. Cyanomethylation of various electrophilic α-carbonyl alkyl radicals prepared from the corresponding bromides was performed in high yield with reagent **1**, under photoredox catalysis (products **9–14**, Scheme 3). Exchanging Ru(bpy)_3_Cl_2_·6H_2_O [*E*_1/2_(Ru^III^/Ru^II*^) = –0.81 V *vs.* SCE] for the more strongly reducing photoexcited-state catalyst *fac*-Ir(ppy)_3_ [*E*_1/2_(Ir^IV^/Ir^III*^) = –1.73 V *vs.* SCE] and increasing the reaction time afforded improved yields for the more challenging substrates **10**, **11** and **14**.^17^ Particularly pleasing was the formation of β-acetoxy ketone **10** in 88% isolated yield, without any obvious trace of elimination under the reaction conditions, highlighting the advantages of an approach which avoids the strong base mediated functionalization of MeCN.

To expand the substrate scope, we sought to utilise imidazolyl thiocarbamates as radical precursors, which have previously been applied in a Barton–McCombie deoxygenation reaction under photoredox catalysis.^18^ Lactic acid derivatives **15** and **16** were produced by deoxygenative cyanomethylation *via* interception of the intermediate α-carbonyl radicals. This approach was also successfully applied to the trapping of heterobenzylic radicals to afford β-heteroarylpropionitriles **17** and **18** in 66% and 60% yields respectively.

Next, to provide a new one-carbon homologation strategy from carboxylic acids to cyanomethyl groups, the cyanomethylation of electronically unactivated alkyl radicals generated from *N*-hydroxyphthalimido esters was investigated.6c,d,^19^ The best results were obtained with the highly reducing photocatalyst *fac*-Ir(5-Fppy)_3_ [*E*_1/2_(Ir^IV^/Ir^III*^) = –1.91 V; *E*_1/2_(Ir^III^/Ir^II^) = –2.18 V *vs.* SCE]^20^ in conjunction with (+)-sodium l-ascorbate, producing products **19–24** resulting from primary radicals in 48–74% yields. The addition of (+)-sodium l-ascorbate was detrimental to the formation of phosphonate product **25**, likely due the lability of the β-phosphonato *N*-hydroxyphthalimido ester.^21^ Excitingly, azide **1** was also competent in intercepting secondary alkyl radicals, *e.g.* to produce cyanomethyl compounds **26–28**, and even afforded product **29** derived from trapping of the electron-rich *N*-Boc pyrrolidinyl radical intermediate, albeit in a modest yield.

Sulfonyl radicals were also efficiently trapped by reagent **1** (products **30–34**), providing a direct access to α-sulfonyl acetonitriles from sulfonyl chlorides and obviating the typical synthetic procedure involving reduction to the intermediate sulfinate followed by alkylation with a halo-acetonitrile reagent.^22^ Resubjection of iodo-α-sulfonyl acetonitrile **34** to the reaction in the presence of *fac*-Ir(5-Fppy)_3_ and (+)-sodium l-ascorbate afforded the di-cyanomethylated product **35** in 46% yield. This result highlighted that aryl radicals can participate in the cyanomethylation reaction to afford arylacetonitriles,^23^ which are valuable synthetic precursors to heterocyclic structures,3*e* and that sequential radical cyanomethylation is possible, with radical formation gated by the redox potentials of the functional groups involved.^24^

To further scope the trapping of aryl radicals with vinyl azide **1**, aryl diazonium salts were explored as radical precursors.^25^ Reaction screening of phenyldiazonium tetrafluoroborate with reagent **1** revealed that the addition of 2,6-lutidine alone was sufficient to produce aryl radical intermediates, affording phenylacetonitrile in 52% yield.^26^ The conditions provided convenient access to substituted arylacetonitriles **36–39** under mild conditions from the corresponding aryldiazonium tetrafluoroborates.

**Scheme 4:**
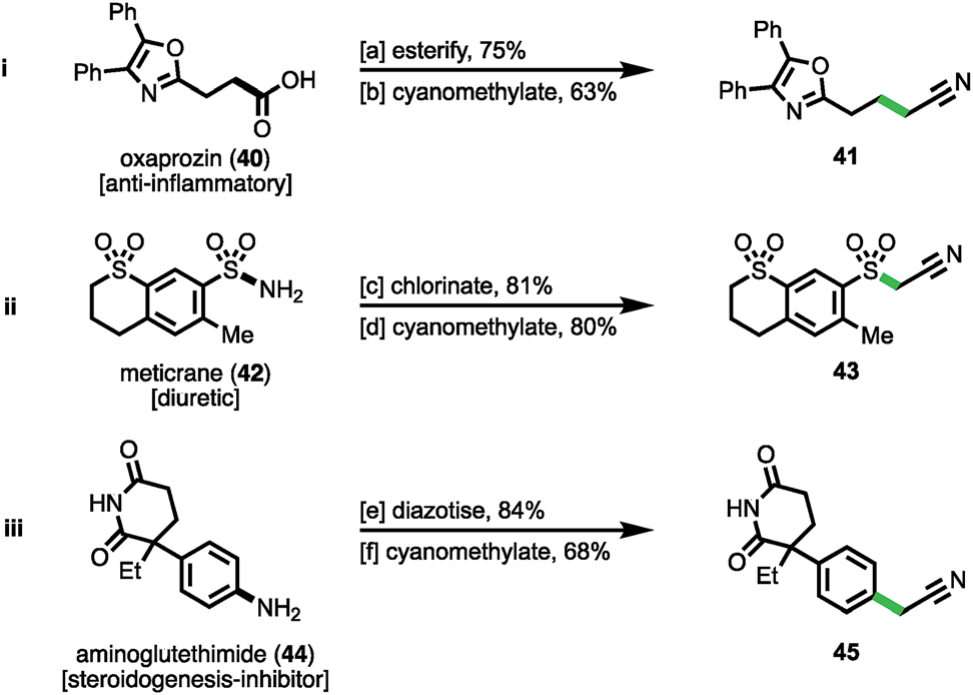
Late-stage cyanomethylation of pharmaceuticals. Reaction conditions: ^a^oxaprozin (**40**), PhthNOH, DCC, DMAP, CH_2_Cl_2_, rt, 16 h; ^b^*N*-hydroxyphthalimido ester (1.0 mmol), **1** (1.5 mmol), *fac*-Ir(5-Fppy)_3_ (0.01 mmol), DMSO (0.2 M), blue LEDs, rt, 24 h; ^c^meticrane (**42**), ClSO_3_H, 100 °C, 2 h; ^d^sulfonyl chloride (1.0 mmol), **1** (1.5 mmol), 2,6-lutidine (1.5 mmol), Ru(bpy)_3_Cl_2_·6H_2_O (0.01 mmol), MeCN (0.2 M), blue LEDs, rt, 4 h; ^e^aminoglutethimide (**44**), NaNO_2_, aq. HBF_4_, H_2_O, 0 °C, 30 min; ^f^diazonium salt (1.0 mmol), **1** (1.5 mmol), 2,6-lutidine (1.5 mmol), MeCN (0.2 M), rt, 4 h.

Finally, to demonstrate the cyanomethylation of radicals in more complex settings, the late stage functionalisation of pharmaceutical agents was undertaken. The *N*-hydroxyphthalimido ester derivative of the nonsteroidal anti-inflammatory (NSAID) oxaprozin (**40**)27*a* was subjected to a decarboxylative cyanomethylation, yielding homologated nitrile **41** in 63% isolated yield (47% from oxaprozin, Scheme 4(i)). Secondly, the sulfonyl chloride derivative of the diuretic meticrane (**42**)27*b* was readily prepared by heating in chlorosulfonic acid; this isolated intermediate was efficiently cyanomethylated with reagent **1** under photoredox catalysis, affording sulfonylacetonitrile **43** in 80% yield (Scheme 4(ii)). Lastly, the aniline bearing aminoglutethimide (**44**) was selected for modification, a compound that acts as a steroidogenesis inhibitor used for the treatment of Cushing's syndrome,27*c* seizures and a number of cancers.27*d* Following diazotization, the isolated salt was efficiently cyanomethylated under the mild reaction conditions (Scheme 4(iii)). These examples help to highlight the diversity of functional groups widely found within medicinally relevant compounds that after activation, can be employed as substrates for the radical cyanomethylation procedure.

## Conclusions

In conclusion, by exploiting the radical decomposition of functionalised vinyl azide **1***via* loss of dinitrogen and fragmentation of the resultant iminyl radical, a cascade-fragmentation approach towards the cyanomethylation of radicals has been developed. Reagent **1** is readily prepared on scale and can be used to intercept α-carbonyl, heterobenzylic, alkyl, sulfonyl and aryl radicals prepared from a range of precursors under both photoredox catalysis and more classical radical generation. This methodology facilitates access to synthetically versatile cyanomethyl groups without the need for cyanide or strong base, under mild conditions, making it amenable to the derivatisation of more complex substrates as demonstrated in the late-stage cyanomethylation of pharmaceutical agents. Further exploration of this reactivity pattern in the design of reagents with which to trap radicals is on-going and will be reported in due course.

## Conflicts of interest

There are no conflicts to declare.

## Supplementary Material

Supplementary information
