# Supplementary material for: Radical cyanomethylation via vinyl azide cascade-fragmentation
Source: Chem Sci. 2019 May 7;10(22):5832–6. doi: 10.1039/c9sc01370a (PMC6568274; doi:10.1039/c9sc01370a)

Electronic Supplementary Material (ESI) for Chemical Science.
This journal is © The Royal Society of Chemistry 2019

# Radical cyanomethylation via vinyl azide cascade-fragmentation

James R. Donald* and Sophie L. Berrell

Department of Chemistry, University of York, York, YO10 5DD, U.K.

* E-mail: james.donald@york.ac.uk

**Supporting Information**

## Table of Contents


iii General information

iii Synthesis of vinyl azide reagents and substrates

viii Optimisation of reaction conditions

ix General procedures

x Images of LED experimental setup

xii Substrate scope

xxvii Quantum yield measurements

xxviii Cyclic voltammetry

xxx References

xxxii $^{1}H$ and $^{13}C$ NMR spectra

## General Information

Except where stated, all reagents were purchased from commercial sources and used without further purification. MeCN, $CH_2Cl_2$ and DMF were dried on an Innovative Technology Inc. PureSolv Solvent Purification System. $^1$H NMR and $^{13}$C NMR spectra were recorded on a JEOL ECX400 or JEOL ECS400 spectrometer, operating at 400 MHz and 100 MHz, respectively. All spectral data were acquired at 295 K. Chemical shifts are reported in parts per million (ppm, δ), downfield from tetramethylsilane (TMS, δ = 0.00 ppm) and are referenced to residual solvent ($CDCl_3$, δ = 7.26 ppm ($^1$H) and 77.16 ppm ($^{13}$C)). Coupling constants (*J*) are reported in Hertz (Hz) and rounded to the nearest 0.5 Hz. The multiplicity abbreviations used are: br broad, s singlet, d doublet, t triplet, q quartet, m multiplet, app apparent. Signal assignment was achieved by analysis of DEPT, COSY, HMBC and HSQC experiments where required. Infrared (IR) spectra were recorded on a PerkinElmer UATR 2 spectrometer, either as a compressed solid or neat oil. High-resolution mass-spectra were obtained by the University of York Mass Spectrometry Service, using electrospray ionization (ESI) or atmospheric pressure chemical ionization (APCI) on a Bruker Daltonics, Micro-tof spectrometer. Melting points were determined using Gallenkamp apparatus. Thin layer chromatography was carried out on Merck silica gel $60F_{254}$ pre-coated aluminum foil sheets and were visualized using UV light (254 nm) and stained with basic aqueous potassium permanganate. Column chromatography was carried out using Fluka silica gel ($SiO_2$), 35–70 µm, 60 Å, under a light positive pressure, eluting with the specified solvent system.

**3-Azido-2-methylbut-3-en-2-ol (1)**

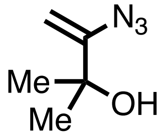


In a modification of the reported procedure,[1] silver carbonate (1.65 g, 6.00 mmol) was added to a solution of 2-methyl-3-butyn-2-ol (**5**) (5.05 g, 6.00 mL, 60.0 mmol), trimethylsilyl azide (10.4 g, 11.9 mL, 90.0 mmol) and water (1.08 g, 1.08 mL, 60 mmol) in anhydrous DMSO (120 mL) at 80 °C, and the resulting mixture was stirred at this temperature for 2 h. After cooling to rt, the reaction was diluted with water (500 mL) and extracted with $Et_2O$ (3 x 200 mL). The combined organic layers were dried ($MgSO_4$), carefully concentrated under reduced pressure (<15 °C bath temperature, > 100 mbar pressure) and the residue purified by silica gel flash column chromatography, eluting with pentane/$Et_2O$ (8:2) to give azide **1** as colourless fibrous needles (6.08 g, 80% yield) which were stored in the freezer to prevent sublimation in the storage vessel.

**$R_f$** 0.22 (8:2 pentane/$Et_2O$); **M.P.** 32–34 °C; **$^1$H NMR** (400 MHz, $CDCl_3$) δ (ppm) 5.10 (d, *J* = 2.5 Hz, 1H), 6.68 (d, *J* = 2.5 Hz, 1H), 1.92 (s, 1H), 1.36 (s, 6H); **$^{13}$C NMR** (100 MHz, $CDCl_3$) δ (ppm) 153.5, 95.2, 71.5, 28.3; **HRMS** as previously observed, **HRMS** data could not be obtained for this compound.[1] All data recorded were in agreement with that previously reported.[1]

**2-Azido-1,1-diphenylprop-2-en-1-ol (7)**

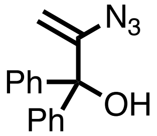


In a modification of the reported procedure,[1] silver carbonate (55 mg, 0.20 mmol) was added to a solution of 1,1-diphenyl-2-propyn-1-ol (**6**) (417 mg, 2.00 mmol), trimethylsilyl azide (346 mg, 398 μL, 3.00 mmol) and water (36 mg, 36 μL, 2.00 mmol) in anhydrous DMSO (8 mL) at 80 °C, and the resulting mixture was stirred at this temperature for 6 h. After cooling to rt, the reaction was diluted with water (30 mL) and extracted with $Et_2O$ (3 x 20 mL). The combined organic layers were dried ($MgSO_4$), concentrated under reduced pressure and the residue purified by silica gel flash column chromatography, eluting with hexane/$Et_2O$ (19:1) to give 2-azido-1,1-diphenylprop-2-en-1-ol (**7**) as colourless solid (260 mg, 52% yield).

$\mathbf{R_f}$ 0.31 (9:1 hexane/$Et_2O$); **M.P.** 44–46 °C; $^1$**H NMR** (400 MHz, $CDCl_3$) δ (ppm) 7.42–7.28 (m, 10H), 5.03 (d, *J* = 2.5 Hz, 1H), 4.72 (d, *J* = 2.5 Hz, 1H), 3.02 (s, 1H); $^{13}$**C NMR** (100 MHz, $CDCl_3$) δ (ppm) 151.0, 143.2, 128.2, 128.0, 127.7, 102.2, 80.7; **IR** (film, $\nu_{max}/cm^{-1}$) 3537, 2155, 2109, 1447, 1262, 1162, 1026; **HRMS** ($ESI^+$) *m*/*z* calcd. for $C_{15}H_{13}N_3NaO$ $[M+Na]^+$ 274.0951, found 274.0948.

**Preparation of novel reaction substrates for substrate scope**

**(±)-2-Bromo-3-oxo-3-phenylpropyl acetate**

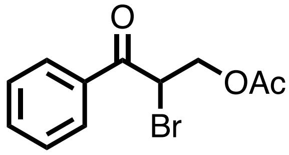


Activated manganese dioxide (17.8 g, 204 mmol) was added to a solution of (±)-(2S/R,3R/S)-2-bromo-3-hydroxy-3-phenylpropyl acetate[2] (1.86 g, 6.81 mmol) in $CH_2Cl_2$ (68 mL) and the resulting suspension stirred at rt for 48 h. The mixture was filtered, washing with $CH_2Cl_2$ (50 mL) and the filtrate concentrated under reduced pressure. The residue was purified by silica gel flash column chromatography, eluting with hexane/EtOAc (8:2) to give (±)-2-bromo-3-oxo-3-phenylpropyl acetate as a colourless low-melting solid (1.14 g, 62% yield).

$\mathbf{R_f}$ 0.38 (7:3 hexane/EtOAc); $^1$**H NMR** (400 MHz, $CDCl_3$) δ (ppm) 8.01 (d, *J* = 7.5 Hz, 2H), 7.62 (t, *J* = 7.5 Hz, 1H), 7.50 (app t, *J* = 7.5 Hz, 2H), 5.32 (dd, *J* = 7.5, 6.0 Hz, 1H), 4.73 (dd, *J* = 11.5, 7.5 Hz, 1H), 4.59 (dd, *J* = 11.5, 6.0 Hz, 1H), 2.05 (s, 3H); $^{13}$**C NMR** (100 MHz, $CDCl_3$) δ (ppm) 191.4, 170.5, 134.3, 134.0, 129.1, 129.0, 63.7, 41.1, 20.8; **IR** (film, $\nu_{max}/cm^{-1}$) 3063, 1741, 1683, 1449, 1366, 1213, 1039; **HRMS** ($ESI^+$) *m*/*z* calcd. for $C_{11}H_{11}{}^{79}BrNaO_3$ $[M+Na]^+$ 292.9784, found 292.9781.

**5-Benzyl-5-bromo-1,3-dimethylpyrimidine-2,4,6(1*H*,3*H*,5*H*)-trione**

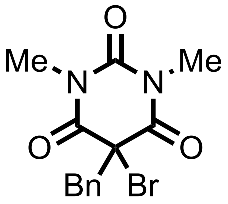


Following a procedure reported for a similar substrate,[3] a solution of bromine (1.60 g, 512 μL, 10.0 mmol) in $CH_2Cl_2$ (5 mL) was added to a solution of 5-benzyl-1,3-dimethylpyrimidine-2,4,6(1*H*,3*H*,5*H*)-trione[4] (2.46 g, 10.0 mmol) and triethylamine (1.01 g, 1.39 mL, 10.0 mmol) in $CH_2Cl_2$ (100 mL) at 0 °C, and the resulting mixture stirred for 10 min at 0 °C. The reaction mixture was washed with water (50 mL) and sat. aq. $NaHCO_3$ (50 mL) then dried (MgSO4) and concentrated under reduced pressure. The residue was purified by silica gel flash column chromatography, eluting with hexane/EtOAc (9:1) to give 5-benzyl-5-bromo-1,3-dimethylpyrimidine-2,4,6(1*H*,3*H*,5*H*)-trione as a colourless solid (2.93 g, 90% yield).

**R$_f$** 0.26 (9:1 hexane/EtOAc); **M.P.** 74–76 °C; **$^1$H NMR** (400 MHz, $CDCl_3$) δ (ppm) 7.26–7.20 (m, 3H), 7.19–7.13 (m, 2H), 3.89 (s, 2H), 3.24 (s, 6H); **$^{13}$C NMR** (100 MHz, $CDCl_3$) δ (ppm) 166.0, 149.6, 133.7, 130.7, 129.0, 128.3, 50.3, 43.2, 29.5; **IR** (film, $\nu_{max}$/cm$^{-1}$) 1671, 1432, 1375, 1286, 1104, 1032; **HRMS** (ESI$^+$) *m*/*z* calcd. for $C_{13}H_{13}{}^{79}BrN_2NaO_3$ [M+Na]$^+$ 347.0002, found 346.9997.

**(±)-Methyl 2-((1*H*-imidazole-1-carbonothioyl)oxy)-3-phenylpropanoate**

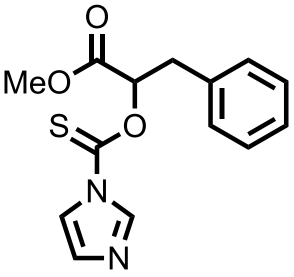


Following a reported procedure for similar substrates,[5] 1,1′-thiocarbonyldiimidazole (1.07 g, 6.00 mmol) was added to a solution of (±)-methyl 2-hydroxy-3-phenylpropanoate[6] (901 mg, 5.00 mmol) and 4-(dimethylamino)pyridine (122 mg, 1.00 mmol) in $CH_2Cl_2$ (25 mL) and the resulting mixture stirred in the dark at rt for 1.5 h, before being concentrated under reduced pressure. The residue was purified by silica gel flash column chromatography, eluting with hexane/EtOAc (1:1) to give (±)-methyl 2-((1*H*-imidazole-1-carbonothioyl)oxy)-3-phenylpropanoate as a pale orange oil (1.44 g, 99% yield).

**R$_f$** 0.34 (1:1 hexane/EtOAc); **$^1$H NMR** (400 MHz, $CDCl_3$) δ (ppm) 8.27 (s, 1H), 7.57 (s, 1H), 7.38–7.22 (m, 5H), 7.03 (s, 1H), 5.86 (dd, *J* = 8.0, 4.5 Hz, 1H), 3.79 (s, 3H), 3.40 (dd, *J* = 14.5, 4.5 Hz, 1H), 3.35 (dd, *J* = 14.5, 8.0 Hz, 1H); **$^{13}$C NMR** (100 MHz, $CDCl_3$) δ (ppm) 183.4, 168.4, 136.9, 134.9, 131.1, 129.2,

129.0, 127.7, 118.3, 80.4, 52.9, 37.6; **IR** (film, $\nu_{max}/cm^{-1}$) 3030, 2953, 1753, 1391, 1320, 1287, 1211, 1106, 1016; **HRMS** ($ESI^+$) *m*/*z* calcd. for $C_{14}H_{14}N_2NaO_3S$ $[M+Na]^+$ 313.0617, found 313.0619.

**(±)-*O*-(1-Morpholino-1-oxopropan-2-yl) 1*H*-imidazole-1-carbothioate**

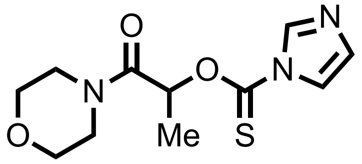


Following a reported procedure for similar substrates,[5] 1,1′-thiocarbonyldiimidazole (1.07 g, 6.00 mmol) was added to a solution of (±)-2-hydroxy-1-morpholinopropan-1-one[7] (796 mg, 5.00 mmol) and 4-(dimethylamino)pyridine (122 mg, 1.00 mmol) in $CH_2Cl_2$ (25 mL) and the resulting mixture stirred in the dark at rt for 4 h, before being concentrated under reduced pressure. The residue was purified by silica gel flash column chromatography, eluting with $CH_2Cl_2$/$Et_2O$/MeOH (19:19:2) to give (±)-*O*-(1-morpholino-1-oxopropan-2-yl) 1*H*-imidazole-1-carbothioate as an orange solid (1.21 g, 90% yield).

**$R_f$** 0.24 (19:19:2 $CH_2Cl_2$/$Et_2O$/MeOH); **M.P.** 67–69 °C; **$^1$H NMR** (400 MHz, $CDCl_3$) δ (ppm) 8.37 (s, 1H), 7.65 (s, 1H), 7.03 (s, 1H), 5.96 (q, *J* = 7.0 Hz, 1H), 3.84–3.43 (m, 8H), 1.67 (d, *J* = 7.0 Hz, 3H); **$^{13}$C NMR** (100 MHz, $CDCl_3$) δ (ppm) 183.6, 167.1, 137.1, 131.1, 118.1, 74.7, 66.9, 66.4, 46.0, 42.6, 16.9; **IR** (film, $\nu_{max}/cm^{-1}$) 3109, 2853, 1648, 1465, 1396, 1323, 1298, 1216, 1109, 1012, 966; **HRMS** ($ESI^+$) *m*/*z* calcd. for $C_{11}H_{15}N_3NaO_3S$ $[M+Na]^+$ 292.0726, found 292.0725.

***O*-((6-Bromopyridin-2-yl)methyl) 1*H*-imidazole-1-carbothioate**

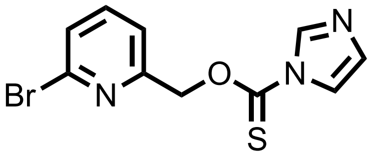


Following a reported procedure for similar substrates,[5] 1,1′-thiocarbonyldiimidazole (1.07 g, 6.00 mmol) was added to a solution of (6-bromo-pyridin-2-yl)methanol (940 mg, 5.00 mmol) and 4-(dimethylamino)pyridine (122 mg, 1.00 mmol) in $CH_2Cl_2$ (25 mL) and the resulting mixture stirred in the dark at rt for 45 min, before being concentrated under reduced pressure. The residue was purified by silica gel flash column chromatography, eluting with hexane/EtOAc (1:1) to give *O*-((6-bromopyridin-2-yl)methyl) 1*H*-imidazole-1-carbothioate as a colourless solid (1.14 g, 77% yield).

**$R_f$** 0.20 (1:1 hexane:EtOAc); **M.P.** 84–85 °C; **$^1$H NMR** (400 MHz, $CDCl_3$) δ (ppm) 8.39 (s, 1H), 7.67 (s, 1H), 7.63 (app t, *J* = 8.0, 1H), 7.51 (d, *J* = 8.0 Hz, 1H) 7.38 (d, *J* = 8.0 Hz, 1H), 7.06 (s, 1H), 5.75 (s, 2H); **$^{13}$C NMR** (100 MHz, $CDCl_3$) δ (ppm) 183.5, 155.0, 142.3, 139.4, 137.1, 131.2, 128.3, 121.2, 118.2, 73.7; **IR** (film, $\nu_{max}/cm^{-1}$) 3112, 1587, 1561, 1471, 1440, 1387, 1284, 1254,

1218, 1121, 1088, 1004; **HRMS** (ESI$^+$) *m/z* calcd. for $C_{10}H_8{}^{79}BrN_3NaOS$ $[M+Na]^+$ 319.9464, found 319.9459.

### *O*-(Pyrazin-2-ylmethyl) 1*H*-imidazole-1-carbothioate

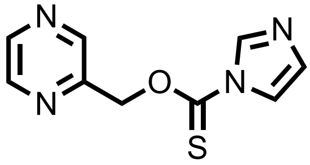

Following a reported procedure for similar substrates,[5] 1,1′-thiocarbonyldiimidazole (1.07 g, 6.00 mmol) was added to a solution of 2-pyrazinylmethanol (551 mg, 5.00 mmol) and 4-(dimethylamino)pyridine (122 mg, 1.00 mmol) in $CH_2Cl_2$ (25 mL) and the resulting mixture stirred in the dark at rt for 1 h, before being concentrated under reduced pressure. The residue was purified by silica gel flash column chromatography, eluting with $CH_2Cl_2$/*i*-PrOH (96:4) to give *O*-(pyrazin-2-ylmethyl) 1*H*-imidazole-1-carbothioate as a pale yellow solid (972 mg, 88% yield).

**R$_f$** 0.18 (96:4 $CH_2Cl_2$/*i*-PrOH); **M.P.** 96–98 °C; **$^1$H NMR** (400 MHz, $CDCl_3$) δ (ppm) 8.74 (s, 1H), 8.63–8.59 (m, 2H), 8.36 (s, 1H), 7.66–7.63 (m, 1H), 7.04 (s, 1H), 5.82 (s, 2H); **$^{13}$C NMR** (100 MHz, $CDCl_3$) δ (ppm) 183.5, 149.4, 145.0, 144.7, 144.2, 137.1, 131.2, 118.1, 72.2; **IR** (film, $\nu_{max}$/cm$^{-1}$) 3119, 1534, 1474, 1396, 1331, 1289, 1263, 1216, 1116, 997; **HRMS** (ESI$^+$) *m/z* calcd. for $C_9H_9N_4OS$ $[M+H]^+$ 221.0492, found 221.0487.

### 1,3-Dioxoisoindolin-2-yl 2-(diethoxyphosphoryl)acetate

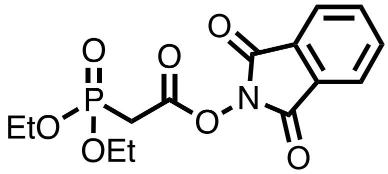

Dicyclohexylcarbodiimide (2.06 g, 12.0 mmol) was added to a solution of diethylphosphonoacetic acid (1.96 g, 10.0 mmol), *N*-hydroxyphthalimide (1.63 g, 10.0 mmol) and 4-(dimethylamino)pyridine (61 mg, 0.50 mmol) in $CH_2Cl_2$ (40 mL) and the mixture stirred at rt for 16 h. The reaction was then filtered through Celite®, washing with $CH_2Cl_2$ (50 mL) and the filtrate concentrated under reduced pressure. The residue was purified by recrystallisation from toluene to give 1,3-dioxoisoindolin-2-yl 2-(diethoxyphosphoryl)acetate as colourless needles (2.16 g, 63%).

**M.P.** 64–66 °C; **$^1$H NMR** (400 MHz, $CDCl_3$) δ (ppm) 7.87–7.82 (m, 2H), 7.79–7.74 (m, 2H), 4.24 (dq, *J* = 8.0, 7.0 Hz, 4H), 3.27 (d, *J* = 21.5 Hz, 2H), 1.36 (t, *J* = 7.0 Hz, 6H); **$^{13}$C NMR** (100 MHz, $CDCl_3$) δ (ppm) 162.4 (d, $J_{C\text{-}P}$ = 6.5 Hz), 161.5, 135.0, 128.8, 124.1, 63.7 (d, $J_{C\text{-}P}$ = 6.5 Hz), 31.2 (d, $J_{C\text{-}P}$ = 134.5 Hz), 16.3 (d, $J_{C\text{-}P}$ = 6.0 Hz); **IR** (film, $\nu_{max}$/cm$^{-1}$) 2991, 2903, 1817, 1786, 1734, 1286, 1259, 1011, 959; **HRMS** (ESI$^+$) *m/z* calcd. for $C_{14}H_{16}NNaO_7P$ $[M+Na]^+$ 364.0557, found 364.0556.

### Optimisation of reaction conditions

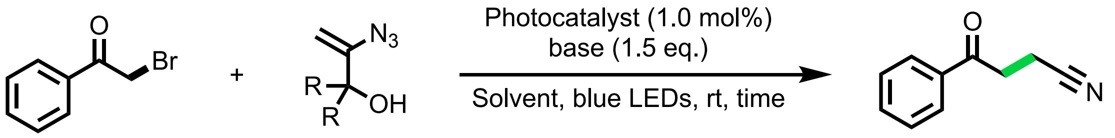


(1.0 eq.)
**8**

(1.5 eq.)
R = Me (**1**), R = Ph (**7**)

**9**

| Entry | Azide | Photocatalyst | Solvent | Time (h) | Base | Yield of 9 (%)[a] |
|---|---|---|---|---|---|---|
| 1 | **1** | *fac*-Ir(ppy)$_3$ | MeCN | 4 | 2,6-lutidine | 97 |
| 2 | **1** | Ir(ppy)$_2$dtbbpyPF$_6$ | MeCN | 4 | 2,6-lutidine | 97 |
| 3 | **1** | Ir(dF(CF$_3$)ppy)$_2$dtbbpyPF$_6$ | MeCN | 4 | 2,6-lutidine | 96 |
| 4 | **1** | Ru(bpy)$_3$Cl$_2$.6H$_2$O | MeCN | 4 | 2,6-lutidine | 93 |
| 5 | **1** | Ru(phen)$_3$(PF$_6$)$_2$ | MeCN | 4 | 2,6-lutidine | 95 |
| 6 | **1** | 4CzIPN | MeCN | 4 | 2,6-lutidine | 95 |
| 7 | **7** | Ru(bpy)$_3$Cl$_2$.6H$_2$O | MeCN | 4 | 2,6-lutidine | 92 |
| 8 | **1** | Ru(bpy)$_3$Cl$_2$.6H$_2$O | $CH_2Cl_2$ | 4 | 2,6-lutidine | 93 |
| 9 | **1** | Ru(bpy)$_3$Cl$_2$.6H$_2$O | DMF | 4 | 2,6-lutidine | 93 |
| 10 | **1** | Ru(bpy)$_3$Cl$_2$.6H$_2$O | MeCN | 1 | 2,6-lutidine | 78 |
| 11 | **1** | Ru(bpy)$_3$Cl$_2$.6H$_2$O | MeCN | 2 | 2,6-lutidine | 93 |
| 12 | **1** | Ru(bpy)$_3$Cl$_2$.6H$_2$O | MeCN | 4 | - | 7 |
| 13 | **1** | - | MeCN | 4 | 2,6-lutidine | 0 |
| 14[b] | **1** | Ru(bpy)$_3$Cl$_2$.6H$_2$O | MeCN | 4 | 2,6-lutidine | 0 |

[a] Reactions performed on a 0.2 mmol scale. Yields were determined by $^1$H NMR integration against 1,3-benzodioxole as an internal standard; [b] no light.

**General procedure A: cyanomethylation of radicals derived from bromides, chlorides and iodides**

An 8 mL vial equipped with a PTFE septum and a magnetic stirrer bar was charged with the halide radical precursor (1.00 mmol), 3-azido-2-methylbut-3-en-2-ol (**1**) (191 mg, 1.50 mmol) and photocatalyst (0.010 mmol). MeCN (5.0 mL) and then 2,6-lutidine (161 mg, 175 µL, 1.5 mmol) were added and the mixture was sparged with Ar for 10 min with stirring. After sparging was complete the septum was additionally sealed with paraffin film and the reaction was irradiated with a 60 W blue LED floodlight (5 cm away) for 4 h, with stirring and cooling from a small fan to maintain an ambient temperature. The mixture was partitioned between EtOAc (3 x 50 mL) and 1 N HCl (50 mL) and the combined organic layers were dried ($MgSO_4$) and concentrated under reduced pressure. The residue was purified by silica gel flash column chromatography, eluting with the solvents indicated to afford the cyanomethylated product.

**General procedure B: cyanomethylation of radicals derived from *O*-thiocarbamates**

An 8 mL vial equipped with a PTFE septum and a magnetic stirrer bar was charged with the *O*-thiocarbamate radical precursor (1.00 mmol), 3-azido-2-methylbut-3-en-2-ol (**1**) (191 mg, 1.50 mmol) and photocatalyst (0.010 mmol). MeCN (5.0 mL) was added and the mixture was sparged with Ar for 10 min with stirring. After sparging was complete the septum was additionally sealed with paraffin film and the reaction was irradiated with a 60 W blue LED floodlight (5 cm away) for 4 h, with stirring and cooling from a small fan to maintain an ambient temperature. The mixture was partitioned between EtOAc (3 x 50 mL) and 1 N HCl (50 mL) and the combined organic layers were dried ($MgSO_4$) and concentrated under reduced pressure. The residue was purified by silica gel flash column chromatography, eluting with the solvents indicated to afford the cyanomethylated product.

**General procedure C: cyanomethylation of radicals derived from *N*-hydroxyphthalimido esters**

An 8 mL vial equipped with a PTFE septum and a magnetic stirrer bar was charged with the *N*-hydroxyphthalimido ester (1.00 mmol), 3-azido-2-methylbut-3-en-2-ol (**1**) (191 mg, 1.50 mmol), *(+)*-sodium L-ascorbate (297 mg, 1.5 mmol) and tris[5-fluoro-2-(2-pyridinyl-kN)phenyl-kC]iridium(III) Ir(5-Fppy)$_3$ (7.1 mg, 0.010 mmol). MeCN (5.0 mL) was added and the mixture was sparged with Ar for 10 min with stirring. After sparging was complete the septum was additionally sealed with paraffin film and the reaction was irradiated with a 60 W blue LED floodlight (5 cm away) for 4 h, with stirring and cooling from a small fan to maintain an ambient temperature. The mixture was partitioned between EtOAc (3 x 50 mL) and water (50 mL) and the combined organic layers were dried ($MgSO_4$) and concentrated under reduced pressure. To aid with the removal of the phthalimide byproduct, crude products were suspended in hexanes or toluene as appropriate, filtered through a plug of cotton and the filtrate concentrated under reduced pressure.

The residue was purified by silica gel flash column chromatography, eluting with the solvents indicated to afford the cyanomethylated product.

### General procedure D: cyanomethylation of radicals derived from aryldiazonium tetrafluoroborates

An 8 mL vial equipped with a PTFE septum and a magnetic stirrer bar was charged with the aryldiazonium tetrafluoroborate (1.00 mmol) and 3-azido-2-methylbut-3-en-2-ol (**1**) (191 mg, 1.50 mmol). MeCN (5.0 mL) was added and the mixture was sparged with Ar for 10 min with stirring. After sparging was complete, 2,6-lutidine was added drop-wise and the septum was additionally sealed with paraffin film and the reaction was stirred at rt for 4 h. The mixture was partitioned between EtOAc (3 x 50 mL) and 1 N HCl (50 mL) and the combined organic layers were dried ($MgSO_4$) and concentrated under reduced pressure. The residue was purified by silica gel flash column chromatography, eluting with the solvents indicated to afford the cyanomethylated product.

### Images of LED experimental setup

### Sparging

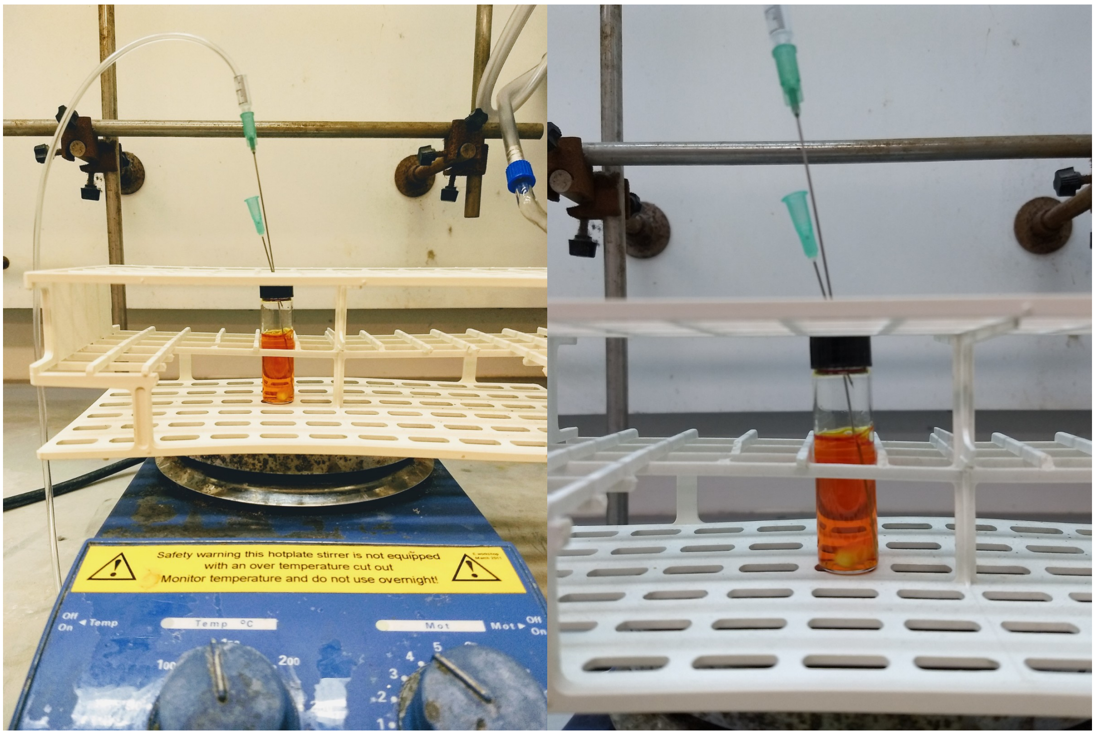

## Sealed reaction ready to run

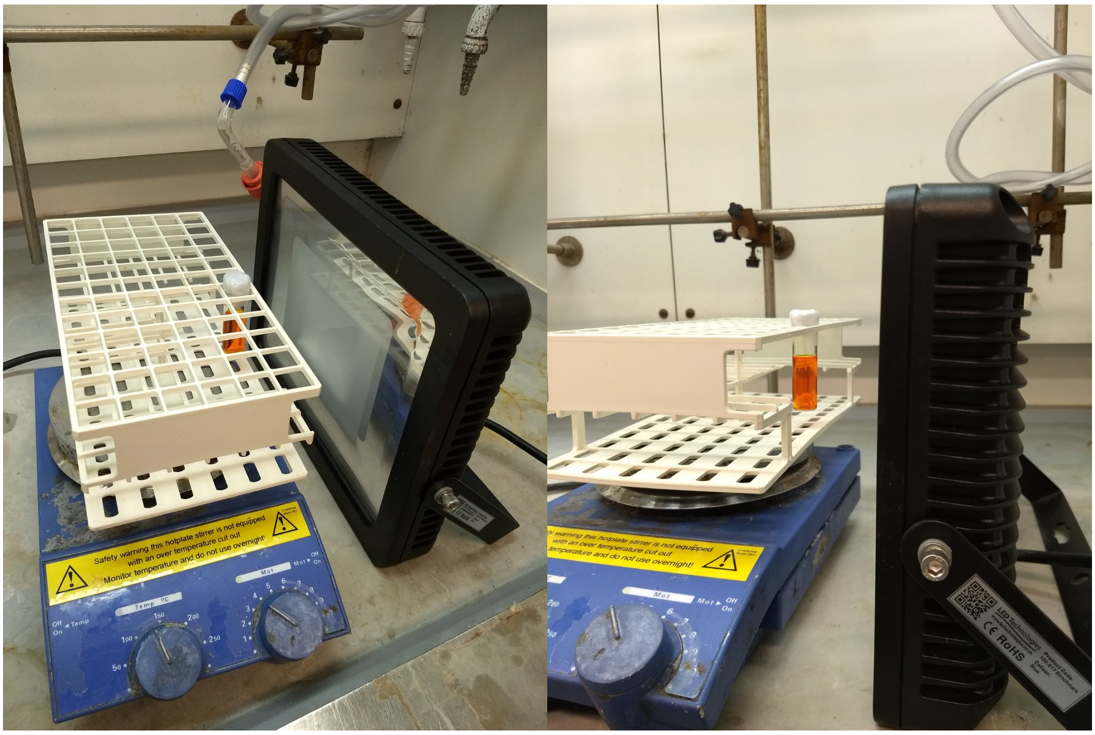


## Reaction in progress

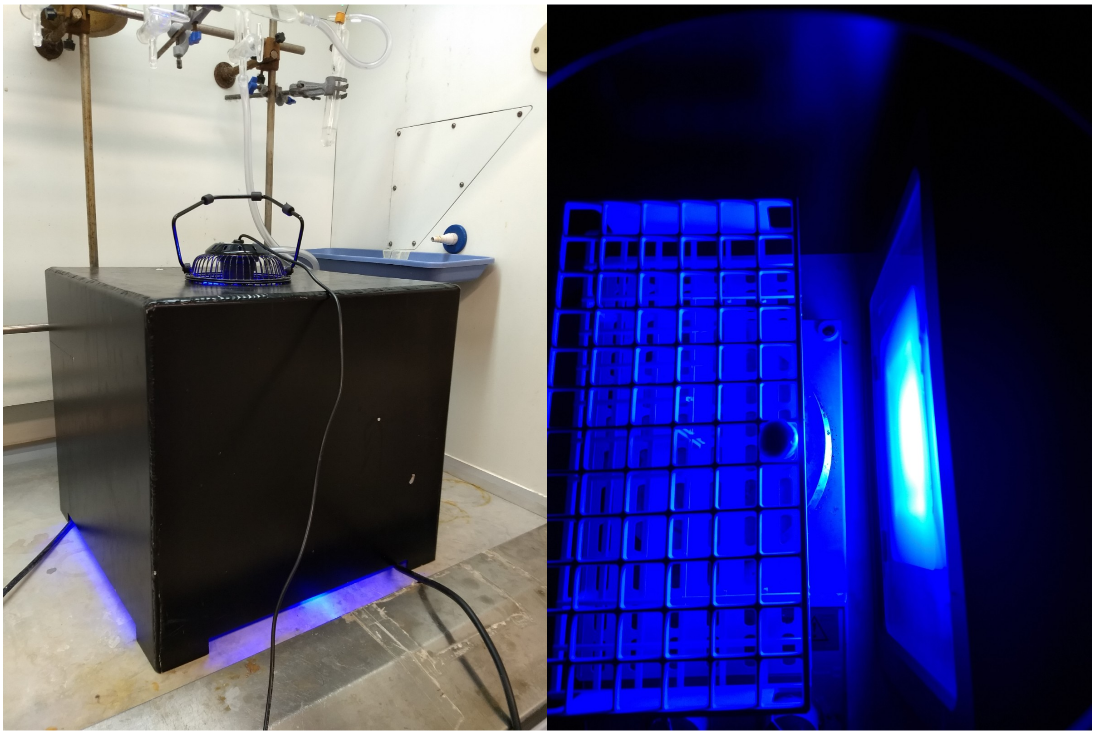

## Substrate scope

### 4-Oxo-4-phenylbutanenitrile (9)

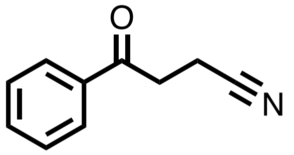

Prepared according to general procedure A using 2-bromoacetophenone (199 mg, 1.00 mmol) and tris(2,2′-bipyridyl)dichlororuthenium(II) hexahydrate [$Ru(bpy)_3Cl_2.6H_2O$] (7.5 mg, 0.010 mmol). The column was eluted with hexane/EtOAc (8:2) to give 4-oxo-4-phenylbutanenitrile (**9**) as a colourless solid (154 mg, 97%).

**$R_f$** 0.27 (7:3 hexane/EtOAc); **$^1$H NMR** (400 MHz, $CDCl_3$) δ (ppm) 7.95 (d, *J* = 8.0 Hz, 2H), 7.61 (t, *J* = 8.0 Hz, 1H), 7.50 (app t, *J* = 8.0, 2H), 3.38 (t, *J* = 7.0 Hz, 2H), 2.77 (t, *J* = 7.0 Hz, 2H); **$^{13}$C NMR** (100 MHz, $CDCl_3$) δ (ppm) 195.5, 135.7, 134.0, 129.0, 128.1, 119.4, 34.4, 11.9; **HRMS** ($ESI^+$) *m/z* calcd. for $C_{10}H_9NNaO$ $[M+Na]^+$ 182.0576, found 182.0577. All data recorded were in agreement with that previously reported.[8]

### (±)-2-(Cyanomethyl)-3-oxo-3-phenylpropyl acetate (10)

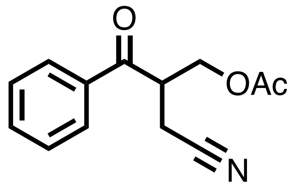

Prepared according to general procedure A using (±)-2-bromo-3-oxo-3-phenylpropyl acetate (271 mg, 1.00 mmol) and *fac*-tris[2-phenylpyridinato-$C_2$,N]iridium(III) [$Ir(ppy)_3$] (6.6 mg, 0.010 mmol). The column was eluted with hexane/EtOAc (8:2 → 7:3) to give (±)-2-(cyanomethyl)-3-oxo-3-phenylpropyl acetate (**10**) as a colourless solid (204 mg, 88%).

**$R_f$** 0.18 (7:3 hexane/EtOAc); **M.P.** 53–55 °C; **$^1$H NMR** (400 MHz, $CDCl_3$) δ (ppm) 7.96 (d, *J* = 7.5 Hz, 2H), 7.63 (t, *J* = 7.5 Hz, 1H), 7.51 (app t, *J* = 7.5, 2H), 4.52 (dd, *J* = 11.5, 5.0 Hz, 1H), 4.22 (dd, *J* = 11.5, 7.0 Hz, 1H), 4.12 (app qd, *J* = 7.0, 5.0 Hz, 1H), 2.84 (dd, *J* = 17.0, 7.0 Hz, 1H), 2.75 (dd, *J* = 17.0, 7.0 Hz, 1H), 2.02 (s, 3H); **$^{13}$C NMR** (100 MHz, $CDCl_3$) δ (ppm) 196.6, 170.1, 135.0, 134.3, 129.2, 128.6, 118.0, 63.8, 43.0, 20.7, 16.7; **IR** (film, $\nu_{max}/cm^{-1}$) 2252, 1733, 1683, 1453, 1364, 1287, 1224, 1037; **HRMS** ($ESI^+$) *m/z* calcd. for $C_{13}H_{13}NNaO_3$ $[M+Na]^+$ 254.0788, found 254.0786.

### Benzyl 3-cyanopropanoate (11)

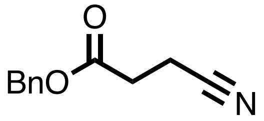

Prepared according to general procedure A using benzyl bromoacetate (229 mg, 158 μL, 1.00 mmol) and *fac*-tris[2-phenylpyridinato-$C_2$,N]iridium(III) [Ir(ppy)$_3$] (6.6 mg, 0.010 mmol). The column was eluted with hexane/EtOAc (8:2) to give benzyl 3-cyanopropanoate (**11**) as a colourless solid (174 mg, 82%).

**R$_f$** 0.14 (8:2 hexane/EtOAc); **$^1$H NMR** (400 MHz, $CDCl_3$) δ (ppm) 7.42–7.32 (m, 5H), 5.17 (s, 2H), 7.76–2.70 (m, 2H), 2.68–2.63 (m, 2H); **$^{13}$C NMR** (100 MHz, $CDCl_3$) δ (ppm) 170.0, 135.2, 128.8, 128.7, 128.6, 118.5, 67.3, 30.0, 13.0; **HRMS** (ESI$^+$) *m/z* calcd. for $C_{11}H_{11}NNaO_2$ $[M+Na]^+$ 212.0682, found 212.0678. All data recorded were in agreement with that previously reported.[9]

**Diethyl 2-(cyanomethyl)-2-methylmalonate (12)**

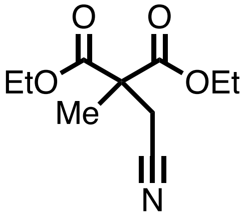


Prepared according to general procedure A using diethyl 2-bromo-2-methylmalonate (253 mg, 191 μL, 1.00 mmol) and tris(2,2′-bipyridyl)dichlororuthenium(II) hexahydrate [Ru(bpy)$_3$Cl$_2$.6H$_2$O] (7.5 mg, 0.010 mmol). The column was eluted with hexane/EtOAc (8:2) to give diethyl 2-(cyanomethyl)-2-methylmalonate (**12**) as a colourless oil (174 mg, 82%).

**R$_f$** 0.20 (8:2 hexane/EtOAc); **$^1$H NMR** (400 MHz, $CDCl_3$) δ (ppm) 4.23 (q, *J* = 7.0 Hz, 4H), 2.90 (s, 2H), 1.61 (s, 3H), 1.27 (t, *J* = 7.0 Hz, 6H); **$^{13}$C NMR** (100 MHz, $CDCl_3$) δ (ppm) 169.5, 116.5, 62.6, 51.6, 24.8, 20.2, 14.0; **IR** (film, $\nu_{max}$/cm$^{-1}$) 2986, 2253, 1731, 1293, 1248, 1200, 1108, 1017; **HRMS** (ESI$^+$) *m/z* calcd. for $C_{10}H_{15}NNaO_4$ $[M+Na]^+$ 236.0893, found 236.0889.

**2-(5-Benzyl-1,3-dimethyl-2,4,6-trioxohexahydropyrimidin-5-yl)acetonitrile (13)**

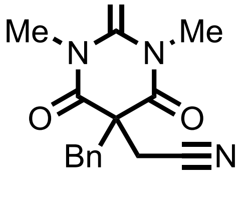


Prepared according to general procedure A using 5-benzyl-5-bromo-1,3-dimethylpyrimidine-2,4,6(1*H*,3*H*,5*H*)-trione (325 mg, 1.00 mmol) and tris(2,2′-bipyridyl)dichlororuthenium(II) hexahydrate [Ru(bpy)$_3$Cl$_2$.6H$_2$O] (7.5 mg, 0.010 mmol). The column was eluted with hexane/EtOAc (7:3) to give diethyl 2-(cyanomethyl)-2-methylmalonate (**13**) as a colourless solid (277 mg, 97%).

**R$_f$** 0.14 (8:2 hexane/EtOAc); **M.P.** 121–122 °C; **$^1$H NMR** (400 MHz, $CDCl_3$) δ (ppm) 7.30–7.22 (m, 3H), 6.93–6.88 (dd, *J* = 7.5, 1.5 Hz, 2H), 3.17 (s, 2H), 3.16 (s, 2H), 3.09 (s, 6H); **$^{13}$C NMR** (100 MHz, $CDCl_3$) δ (ppm) 168.8, 149.6, 132.7, 129.0, 128.9, 128.8, 115.9, 56.1, 47.2, 28.8, 24.2; **IR** (film, $\nu_{max}$/cm$^{-1}$)

2934, 2250, 1675, 1433, 1378, 1392, 1288, 1109, 1054; **HRMS** (ESI$^+$) *m*/*z* calcd. for $C_{15}H_{15}KN_3O_3$ [M+K]$^+$ 324.0745, found 324.0740.

**3-(Phenylsulfonyl)propanenitrile (14)**

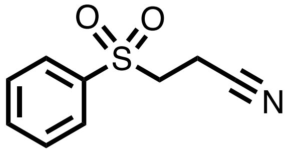

Prepared according to general procedure A using bromomethyl phenyl sulfone (235 mg, 1.00 mmol) and *fac*-tris[2-phenylpyridinato-$C_2$,N]iridium(III) [Ir(ppy)$_3$] (6.6 mg, 0.010 mmol), with the reaction run for 24 h. The column was eluted with hexane/EtOAc (7:3 → 6:4) to give 3-(phenylsulfonyl)propanenitrile (**14**) as a colourless solid (156 mg, 80%).

**R$_f$** 0.20 (6:4 hexane/EtOAc); **$^1$H NMR** (400 MHz, $CDCl_3$) δ (ppm) 7.93 (t, *J* = 8.0 Hz, 2H), 7.74 (t, *J* = 8.0 Hz, 1H), 7.63 (app t, *J* = 8.0 Hz, 2H), 3.39 (t, *J* = 7.5 Hz, 2H), 2.82 (t, *J* = 7.5 Hz, 2H); **$^{13}$C NMR** (100 MHz, $CDCl_3$) δ (ppm) 137.6, 134.9, 129.9, 128.4, 116.1, 51.2, 12.1; **HRMS** (ESI$^+$) *m*/*z* calcd. for $C_9H_9NNaO_2S$ [M+Na]$^+$ 218.0246, found 218.0242. All data recorded were in agreement with that previously reported.[10]

**(±)-Methyl 2-benzyl-3-cyanopropanoate (15)**

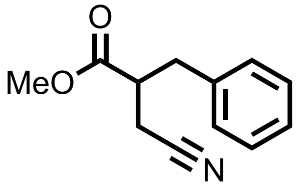

Prepared according to general procedure B using (±)-methyl 2-((1*H*-imidazole-1-carbonothioyl)oxy)-3-phenylpropanoate (290 mg, 1.00 mmol). The column was eluted with hexane/EtOAc (8:2) to give (±)-methyl 2-benzyl-3-cyanopropanoate (**15**) as a colourless oil (165 mg, 81%).

**R$_f$** 0.18 (8:2 hexane/EtOAc); **$^1$H NMR** (400 MHz, $CDCl_3$) δ (ppm) 7.32 (app t, *J* = 7.5 Hz, 2H), 7.26 (t, *J* = 7.5 Hz, 1H), 7.17 (d, *J* = 7.5 Hz, 2H), 3.74 (s, 3H), 3.17 (dd, *J* = 13.0, 5.5 Hz, 1H), 3.05–2.97 (m, 1H), 2.94 (dd, *J* = 13.0, 8.0 Hz, 1H), 2.52 (d, *J* = 6.5 Hz, 2H); **$^{13}$C NMR** (100 MHz, $CDCl_3$) δ (ppm) 172.5, 136.8, 129.1, 129.0, 127.4, 117.8, 52.6, 43.3, 36.9, 18.5; **HRMS** (ESI$^+$) *m*/*z* calcd. for $C_9H_9NNaO_2S$ [M+Na]$^+$ 218.0246, found 218.0245. All data recorded were in agreement with that previously reported.[11]

**(±)-3-Methyl-4-morpholino-4-oxobutanenitrile (16)**

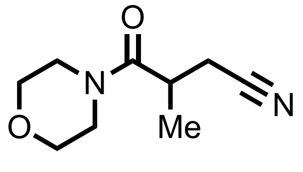

Prepared according to general procedure B using (±)-*O*-(1-morpholino-1-oxopropan-2-yl) 1*H*-imidazole-1-carbothioate (269 mg, 1.00 mmol). The column was eluted with EtOAc to give (±)-3-methyl-4-morpholino-4-oxobutanenitrile (**16**) as a pale yellow oil (92 mg, 51%).

**R$_f$** 0.28 (EtOAc); **$^1$H NMR** (400 MHz, $CDCl_3$) δ (ppm) 3.72–3.54 (m, 6H), 3.52–3.39 (m, 2H), 3.01 (app sextet, *J* = 7.0 Hz, 1H), 2.63 (dd, *J* = 17.0, 7.0 Hz, 1H), 2.50 (dd, *J* = 17.0, 7.0 Hz, 1H), 1.23 (d, *J* = 7.0 Hz, 3H); **$^{13}$C NMR** (100 MHz, $CDCl_3$) δ (ppm) 171.4, 118.7, 66.8, 66.6, 46.0, 42.3, 33.1, 21.4, 17.5; **IR** (film, $\nu_{max}$/cm$^{-1}$) 3063, 1741, 1683, 1449, 1366, 1213, 1039; **HRMS** (ESI$^+$) *m*/*z* calcd. for $C_9H_{14}N_2NaO_2$ $[M+Na]^+$ 205.0947, found 205.0948.

**3-(6-Bromopyridin-2-yl)propanenitrile (17)**

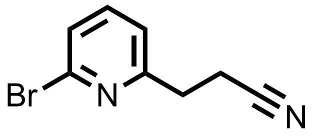


Prepared according to general procedure B using *O*-((6-bromopyridin-2-yl)methyl) 1*H*-imidazole-1-carbothioate (298 mg, 1.00 mmol). During extraction, sat. aq. $NaHCO_3$ (50 mL) was used in place of 1 N HCl. The column was eluted with hexane/EtOAc (8:2) to give 3-(6-bromopyridin-2-yl)propanenitrile (**17**) as a pale yellow oil (140 mg, 66%).

**R$_f$** 0.15 (8:2 hexane/EtOAc); **$^1$H NMR** (400 MHz, $CDCl_3$) δ (ppm) 7.52 (app t, *J* = 7.5 Hz, 1H), 7.39 (d, *J* = 7.5 Hz, 1H), 7.20 (d, *J* = 7.5 Hz, 1H), 3.09 (t, *J* = 7.0 Hz, 2H), 2.83 (t, *J* = 7.0 Hz, 2H); **$^{13}$C NMR** (100 MHz, $CDCl_3$) δ (ppm) 158.7, 142.2, 139.3, 126.9, 122.2, 119.1, 33.2, 16.9; **IR** (film, $\nu_{max}$/cm$^{-1}$) 2935, 2248, 1582, 1554, 1435, 1406, 1121; **HRMS** (ESI$^+$) *m*/*z* calcd. for $C_8H_7{}^{79}BrN_2Na$ $[M+Na]^+$ 232.9685, found 232.9683.

**3-(Pyrazin-2-yl)propanenitrile (18)**

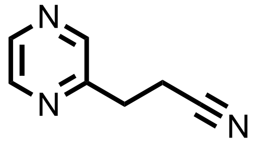


Prepared according to general procedure B using *O*-(pyrazin-2-ylmethyl) 1*H*-imidazole-1-carbothioate (220 mg, 1.00 mmol). During extraction, sat. aq. $NaHCO_3$ (50 mL) was used in place of 1 N HCl. The column was eluted with EtOAc to give 3-(pyrazin-2-yl)propanenitrile (**18**) as a pale yellow oil (80 mg, 60%).

**R$_f$** 0.28 (EtOAc); **$^1$H NMR** (400 MHz, $CDCl_3$) δ (ppm) 8.47 (s, 2H), 8.43 (s, 1H), 3.11 (t, *J* = 7.5 Hz, 2H), 2.81 (t, *J* = 7.5 Hz, 2H); **$^{13}$C NMR** (100 MHz, $CDCl_3$) δ (ppm) 152.8, 144.5, 144.2, 143.3, 119.0, 30.4, 15.8; **IR** (film, $\nu_{max}$/cm$^{-1}$) 3059, 2933, 2248, 1528, 1477, 1406, 1135, 1060, 1020; **HRMS** (APCI$^+$) *m*/*z* calcd. for $C_7H_8N_3$ $[M+H]^+$ 134.0713, found 134.0708.

**Tridecanenitrile (19)**

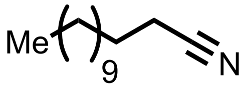

Prepared according to general procedure C using 1,3-dioxoisoindolin-2-yl dodecanoate[12] (345 mg, 1.00 mmol), with the reaction run for 24 h. The column was eluted with *n*-hexane/$Et_2O$ (9:1) to give tridecanenitrile (**19**) as a pale yellow oil (133 mg, 68%).

**R$_f$** 0.30 (9:1 *n*-hexane/$Et_2O$); **$^1$H NMR** (400 MHz, $CDCl_3$) δ (ppm) 2.33 (t, *J* = 7.0 Hz, 2H), 1.65 (app quintet, *J* = 7.0 Hz, 2H), 1.44 (app quintet, *J* = 7.0 Hz, 2H), 1.34–1.22 (m, 16H), 0.88 (t, J = 7.0 Hz, 3H); **$^{13}$C NMR** (100 MHz, $CDCl_3$) δ (ppm) 120.0, 32.0, 29.7 (2C), 29.6, 29.5, 29.4, 28.9, 28.8, 25.5, 22.8, 17.3, 14.2; **HRMS** ($ESI^+$) *m*/*z* calcd. for $C_{13}H_{25}NNa$ $[M+Na]^+$ 218.1879, found 218.1880. All data recorded were in agreement with that previously reported.[13]

**4-Phenylbutanenitrile (20)**

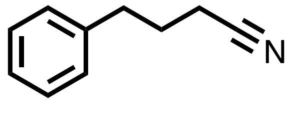

Prepared according to general procedure C using 1,3-dioxoisoindolin-2-yl 3-phenylpropanoate[14] (295 mg, 1.00 mmol). The column was eluted with *n*-pentane/$Et_2O$ (9:1) to give 4-phenylbutanenitrile (**20**) as a colourless oil (101 mg, 70%).

**R$_f$** 0.17 (9:1 *n*-pentane/$Et_2O$); **$^1$H NMR** (400 MHz, $CDCl_3$) δ (ppm) 7.32 (app t, *J* = 7.5 Hz, 2H), 7.23 (t, *J* = 7.5 Hz, 1H), 7.19 (d, *J* = 7.5 Hz, 2H), 2.79 (t, *J* = 7.0 Hz, 2H), 2.32 (t, J = 7.0 Hz, 2H), 1.99 (app quintet, *J* = 7.0 Hz, 2H); **$^{13}$C NMR** (100 MHz, $CDCl_3$) δ (ppm) 69.9, 58.9, 58.7, 56.8, 49.8, 35.4, 42.9, 53.4; **HRMS** ($APCI^+$) *m*/*z* calcd. for $C_{10}H_{12}N$ $[M+H]^+$ 146.0964, found 146.0961. All data recorded were in agreement with that previously reported.[15]

**6-Oxo-6-phenylhexanenitrile (21)**

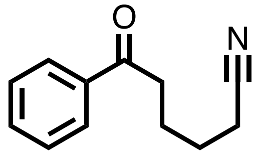

Prepared according to general procedure C using 1,3-dioxoisoindolin-2-yl 5-oxo-5-phenylpentanoate[16] (337 mg, 1.00 mmol). The column was eluted with toluene/$Et_2O$ (19:1) to give 6-oxo-6-phenylhexanenitrile (**21**) as a colourless solid (122 mg, 65%).

**R$_f$** 0.30 (19:1 toluene/$Et_2O$); **$^1$H NMR** (400 MHz, $CDCl_3$) δ (ppm) 7.95 (d, *J* = 7.5 Hz, 2H), 7.58 (t, *J* = 7.5 Hz, 1H), 7.47 (app t, *J* = 7.5 Hz, 2H), 3.05 (t, *J* = 7.0 Hz, 2H), 2.41 (t, *J* = 7.0 Hz, 2H), 1.96–1.87 (m, 2H), 1.82–1.72 (m, 2H); **$^{13}$C NMR** (100 MHz, $CDCl_3$) δ (ppm) 199.3, 136.9, 133.5, 128.9, 128.2, 119.8,

37.6, 25.3, 23.3, 17.5; **HRMS** ($APCI^+$) *m/z* calcd. for $C_{12}H_{13}NNaO$ $[M+Na]^+$ 210.0889, found 210.0890. All data recorded were in agreement with that previously reported.[17]

**(*S*)-*tert*-Butyl 2-((*tert*-butoxycarbonyl)amino)-5-cyanopentanoate (22)**

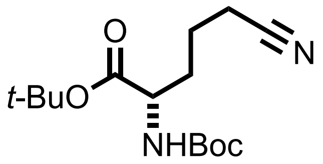


Prepared according to general procedure C using (*S*)-1-*tert*-butyl 5-(1,3-dioxoisoindolin-2-yl) 2-((*tert*-butoxycarbonyl)amino)pentanedioate[18] (345 mg, 1.00 mmol). The column was eluted with hexane/$Et_2O$ (9:1) to give (*S*)-*tert*-butyl 2-((*tert*-butoxycarbonyl)amino)-5-cyanopentanoate (**22**) as a pale yellow oil (215 mg, 72%).

**$R_f$** 0.10 (7:3 hexane/$Et_2O$); $[\alpha]_D^{22}$ +13.4 (c 1.00, $CHCl_3$); **$^1$H NMR** (400 MHz, $CDCl_3$) δ (ppm) (rotamers) 5.11 (br d, *J* = 7.5 Hz, 0.88H, major rotamer), 4.92 (br s, 0.12 H, minor rotamer), 4.25–4.08 (m, 0.88H, major rotamer), 4.00 (br s, 0.12H, minor rotamer), 2.46–2.30 (m, 2H), 2.00–1.85 (m, 1H), 1.80–1.59 (m, 3H), 1.44 (s, 9H), 1.41 (s, 9H); **$^{13}$C NMR** (100 MHz, $CDCl_3$) δ (ppm) 171.3, 155.5, 119.3, 82.5, 80.0, 53.1, 32.1, 28.4, 28.0, 21.6, 16.9; **HRMS** ($ESI^+$) *m/z* calcd. for $C_{15}H_{27}N_2O_4$ $[M+H]^+$ 299.1965, found 299.1962. All data recorded were in agreement with that previously reported.[19]

***tert*-Butyl 3-(2-cyanoethyl)azetidine-1-carboxylate (23)**

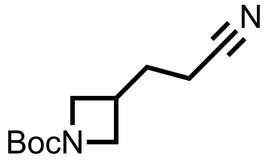


Prepared according to general procedure C using *tert*-butyl 3-(2-((1,3-dioxoisoindolin-2-yl)oxy)-2-oxoethyl)azetidine-1-carboxylate[20] (360 mg, 1.00 mmol), with the reaction run for 24 h. The column was eluted with hexane/EtOAc (3:2) to give *tert*-Butyl 3-(2-cyanoethyl)azetidine-1-carboxylate (**23**) as a pale yellow oil (155 mg, 74%).

**$R_f$** 0.13 (3:2 hexane/EtOAc); **$^1$H NMR** (400 MHz, $CDCl_3$) δ (ppm) 4.07 (app t, *J* = 8.0 Hz, 2H), 3.58 (dd, *J* = 8.0, 5.5 Hz, 2H), 2.64 (app quintet of t, *J* = 8.0, 5.5 Hz, 1H), 2.34 (t, *J* = 7.0 Hz, 2H), 1.97 (dt, *J* = 7.5, 7.0 Hz, 2H), 1.43 (s, 9H); **$^{13}$C NMR** (100 MHz, $CDCl_3$) δ (ppm) 156.4, 119.0, 79.8, 53.7, 29.9, 28.5, 28.1, 15.2; **HRMS** (ESI) *m/z* calcd. for $C_{11}H_{18}N_2O_2Na$ $[M+Na]^+$ 223.1266, found 233.1259. All data recorded were in agreement with that previously reported.[21]

**3-(1,3-Dioxoisoindolin-2-yl)propanenitrile (24)**

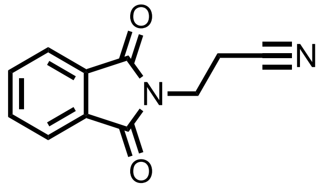

Prepared according to general procedure C using 1,3-dioxoisoindolin-2-yl 2-(1,3-dioxoisoindolin-2-yl)acetate[22] (350 mg, 1.00 mmol). The column was eluted with hexane/$Et_2O$ (1:1) to give 3-(1,3-dioxoisoindolin-2-yl)propanenitrile (**24**) as a colourless solid (97 mg, 48%).

$R_f$ 0.20 (1:1 hexane/$Et_2O$); **$^1$H NMR** (400 MHz, $CDCl_3$) δ (ppm) 7.91–7.85 (m, 2H), 7.79–7.73 (m, 2H), 4.01 (t, *J* = 7.0 Hz, 2H), 2.81 (t, *J* = 7.0 Hz, 2H); **$^{13}$C NMR** (100 MHz, $CDCl_3$) δ (ppm) 167.7, 134.6, 131.8, 123.8, 116.9, 33.6, 17.3; **HRMS** ($ESI^+$) *m*/*z* calcd. for $C_{11}H_9N_2O_2$ $[M+H]^+$ 201.0659, found 201.0657. All data recorded were in agreement with that previously reported.[23]

**Diethyl (2-cyanoethyl)phosphonate (25)**

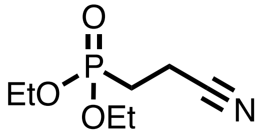

Prepared according to general procedure C using 1,3-dioxoisoindolin-2-yl 2-(diethoxyphosphoryl)acetate (341 mg, 1.00 mmol) without the addition of *(+)*-sodium L-ascorbate. The column was eluted with EtOAc to give diethyl (2-cyanoethyl)phosphonate (**25**) as a yellow oil (107 mg, 56%).

$R_f$ 0.20 (EtOAc); **$^1$H NMR** (400 MHz, $CDCl_3$) δ (ppm) 4.21–4.06 (m, 4H), 2.62 (dt, *J* = 15.5, 8.0 Hz, 2H), 2.07 (dt, *J* = 18.0, 8.0 Hz, 2H), 1.34 (t, *J* = 7.0 Hz, 6H); **$^{13}$C NMR** (100 MHz, $CDCl_3$) δ (ppm) 118.4 (d, $J_{C\text{-}P}$ = 18.0 Hz), 62.5 (d, $J_{C\text{-}P}$ = 6.5 Hz), 22.2 (d, $J_{C\text{-}P}$ = 146.0 Hz), 16.5 (d, $J_{C\text{-}P}$ = 6.0 Hz), 11.7 (d, $J_{C\text{-}P}$ = 4.0 Hz); **HRMS** ($ESI^+$) *m*/*z* calcd. for $C_7H_{14}NNaO_3P$ $[M+Na]^+$ 214.0604, found 214.0599. All data recorded were in agreement with that previously reported.[24]

***tert*-Butyl 4-(cyanomethyl)piperidine-1-carboxylate (26)**

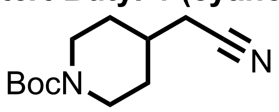

Prepared according to general procedure C using 1-(*tert*-butyl) 4-(1,3-dioxoisoindolin-2-yl) piperidine-1,4-dicarboxylate[25] (374 mg, 1.00 mmol). The column was eluted with hexane/$Et_2O$ (3:2) to give *tert*-butyl 4-(cyanomethyl)piperidine-1-carboxylate **26**) as a pale yellow solid (133 mg, 59%).

$R_f$ 0.16 (3:2 hexane/$Et_2O$); **$^1$H NMR** (400 MHz, $CDCl_3$) δ (ppm) 4.16 (m, 2H), 2.78–2.61 (m, 2H), 2.31 (d, *J* = 6.0 Hz, 2H), 1.89–1.75 (m, 3H), 1.45 (s, 9H), 1.29–1.20 (m, 2H); **$^{13}$C NMR** (100 MHz, $CDCl_3$) δ (ppm) 154.8, 118.3, 79.9,

43.7, 33.5, 31.4, 28.6, 24.3; **HRMS** (ESI) *m/z* calcd. for $C_{12}H_{20}N_2O_2Na$ $[M+Na]^+$ 247.1422, found 247.1414. All data recorded were in agreement with that previously reported.[26]

**2-(tetrahydro-2*H*-pyran-4-yl)acetonitrile (27)**

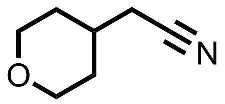

Prepared according to general procedure C using 1,3-dioxoisoindolin-2-yl tetrahydro-2*H*-pyran-4-carboxylate[27] (275 mg, 1.00 mmol). The column was eluted with hexane/$Et_2O$ (4:1) to give 2-(tetrahydro-2*H*-pyran-4-yl)acetonitrile (**27**) as a pale yellow oil (69 mg, 55%).

**$R_f$** 0.17 (3:2 $Et_2O$/hexane); **$^1$H NMR** (400 MHz, $CDCl_3$) δ (ppm) 4.03–3.97 (m, 2H), 3.39 (app td, *J* = 12.0, 2.0 Hz, 2H), 2.31 (d, *J* = 7.0 Hz, 2H), 1.98–1.86 (m, 1H), 1.78–1.71 (m, 2H), 1.43 (app qd, *J* = 12.0, 4.5 Hz, 2H); **$^{13}$C NMR** (100 MHz, $CDCl_3$) δ (ppm) 118.2, 67.5, 32.4, 32.2, 24.5; **HRMS** (ESI) *m/z* calcd. for $C_7H_{12}NO$ $[M+H]^+$ 126.0913, found 126.0908. All data recorded were in agreement with that previously reported.[28]

**2-(2,3-Dihydro-1*H*-inden-2-yl)acetonitrile (28)**

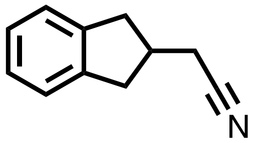

Prepared according to general procedure C using 1,3-dioxoisoindolin-2-yl 2,3-dihydro-1H-indene-2-carboxylate[29] (307 mg, 1.00 mmol). The column was eluted with *n*-pentane/$Et_2O$ (9:1) to give 2-(2,3-dihydro-1*H*-inden-2-yl)acetonitrile (**28**) as a colourless solid (57 mg, 36%).

**$R_f$** 0.20 (9:1 *n*-pentane/$Et_2O$); **M.P.** 49–51 °C; **$^1$H NMR** (400 MHz, $CDCl_3$) δ (ppm) 7.34–7.22 (m, 4H), 3.27 (dd, *J* = 15.0, 7.0 Hz, 2H), 2.97–2.81 (m, 3H), 2.56 (d, *J* = 7.0 Hz, 2H); **$^{13}$C NMR** (100 MHz, $CDCl_3$) δ (ppm) 141.5, 126.9, 124.8, 119.1, 38.6, 36.2, 22.7; **IR** (film, $\nu_{max}/cm^{-1}$) 2955, 2908, 2840, 2240, 1477, 1462, 1416, 1244, 1016, 776, 750; **HRMS** ($ESI^+$) *m*/*z* calcd. for $C_{11}H_{11}NNa$ $[M+Na]^+$ 180.0784, found 180.0777.

**(±)-*tert*-Butyl 2-(cyanomethyl)pyrrolidine-1-carboxylate (29)**

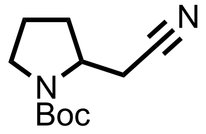

Prepared according to general procedure C using (±)-1-*tert*-butyl 2-(1,3-dioxoisoindolin-2-yl) pyrrolidine-1,2-dicarboxylate[30] (374 mg, 1.00 mmol). The column was eluted with hexane/$Et_2O$ (7:3) to give (±)-*tert*-Butyl 2-(cyanomethyl)pyrrolidine-1-carboxylate (**29**) as a colourless oil (73 mg, 35%).

**$R_f$** 0.10 (7:3 hexane/$Et_2O$); **$^1$H NMR** (400 MHz, $CDCl_3$) δ (ppm) 4.05–3.95 (m, 1H), 3.50–3.34 (m, 2H), 2.89–2.50 (m, 2H), 2.22–2.10 (m, 1H), 2.08–2.18 (m, 3H), 1.50–1.42 (m, 9H); **$^{13}$C NMR** (100 MHz, $CDCl_3$) δ (ppm) (rotamers) 154.5 (major rotamer), 153.9 (minor rotamer), 118.1 (major rotamer), 117.8 (minor rotamer), 80.4 (minor rotamer), 80.0 (major rotamer), 53.7 (both rotamers), 47.2 (major rotamer), 46.7 (minor rotamer), 31.3 (minor rotamer), 30.4 (major rotamer), 28.5 (major rotamer), 28.3 (minor rotamer), 23.7 (major rotamer), 23.1 (minor rotamer), 22.9 (minor rotamer), 22.3 (major rotamer); **HRMS** ($ESI^+$) *m/z* calcd. for $C_{11}H_{18}N_2NaO_2$ $[M+Na]^+$ 233.1260, found 233.1257. All data recorded were in agreement with that previously reported.[31]

**2-(Cyclohexylsulfonyl)acetonitrile (30)**

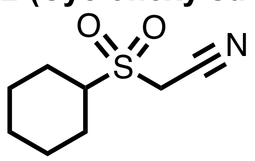


Prepared according to general procedure A using cyclohexanesulfonyl chloride (251 mg, 145 µL, 1.00 mmol) and *fac*-tris[2-phenylpyridinato-$C_2$,N]iridium(III) [Ir(ppy)$_3$] (6.6 mg, 0.010 mmol). The column was eluted with hexane/EtOAc (7:3) to give 2-(cyclohexylsulfonyl)acetonitrile (**30**) as a colourless solid (187 mg, 99%).

**$R_f$** 0.25 (7:3 hexane/EtOAc); **$^1$H NMR** (400 MHz, $CDCl_3$) δ (ppm) 3.93 (s, 2H), 3.28 (tt, J = 12.0, 3.5 Hz, 1H), 2.26–2.18 (m, 2H), 2.03–1.95 (m, 2H), 1.82–1.73 (m, 1H), 1.64 (app qd, J = 12.5, 3.0 Hz, 2H), 1.37 (app qt, J = 12.5, 3.0 Hz, 2H), 1.25 (app qt 12.5, 3.0 Hz, 1H); **$^{13}$C NMR** (100 MHz, $CDCl_3$) δ (ppm) 110.6, 62.4, 39.6, 25.2, 24.9 (2C); **HRMS** ($ESI^+$) *m/z* calcd. for $C_8H_{13}NNaO_2S$ $[M+Na]^+$ 210.0559, found 210.0556. All data recorded were in agreement with that previously reported.[32]

**2-((((1*R/S*,4*S/R*)-7,7-dimethyl-2-oxobicyclo[2.2.1]heptan-1-yl)methyl) sulfonyl) acetonitrile (31)**

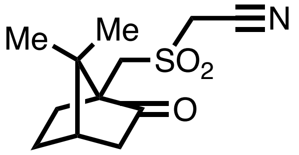


Prepared according to general procedure A using (±)-10-camphorsulfonyl chloride (251 mg, 1.00 mmol) and *fac*-tris[2-phenylpyridinato-$C_2$,N]iridium(III) [Ir(ppy)$_3$] (6.6 mg, 0.010 mmol). The column was eluted with hexane/EtOAc (7:3) to give 2-((((1*R/S*,4*S/R*)-7,7-dimethyl-2-oxobicyclo[2.2.1]heptan-1-yl)methyl)sulfonyl) acetonitrile (**31**) as a colourless solid (181 mg, 71%).

**$R_f$** 0.28 (6:4 hexane/EtOAc); **M.P.** 92–94 °C; **$^1$H NMR** (400 MHz, $CDCl_3$) δ (ppm) 4.74 (d, *J* = 16.5 Hz, 1H), 4.17 (dd, *J* = 16.5, 1.5 Hz, 1H), 3.70 (d, *J* = 15.5 Hz, 1H), 2.97 (dd, *J* = 15.5, 1.5 Hz, 1H), 2.43 (ddd, *J* = 18.5, 4.5, 3.0 Hz, 1H), 2.24–2.15 (m, 2H), 2.12–1.92 (m, 3H), 1.51 (ddd, J = 12.5, 9.0, 3.0 Hz, 1H), 1.03 (s, 3H), 0.94 (s, 3H); **$^{13}$C NMR** (100 MHz, $CDCl_3$) δ (ppm) 216.4,

111.2, 59.6, 52.9, 49.5, 46.3, 42.9, 42.7, 27.2, 26.2, 19.9, 19.4; **IR** (film, $\nu_{max}/cm^{-1}$) 2973, 2927, 2257, 1739, 1318, 1141, 1049, 1068; **HRMS** ($ESI^+$) *m*/*z* calcd. for $C_{12}H_{17}NNaO_3S$ $[M+Na]^+$ 278.0821, found 278.0823.

**2-Tosylacetonitrile (32)**

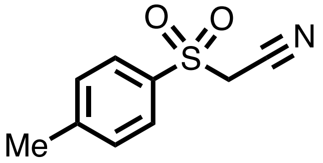

Prepared according to general procedure A using tosyl chloride (191 mg, 1.00 mmol) and tris(2,2′-bipyridyl)dichlororuthenium(II) hexahydrate [$Ru(bpy)_3Cl_2.6H_2O$] (7.5 mg, 0.010 mmol). The column was eluted with hexane/EtOAc (7:3) to give 2-tosylacetonitrile (**32**) as a colourless solid (181 mg, Quant.).

**$R_f$** 0.22 (7:3 hexane/EtOAc); **$^1$H NMR** (400 MHz, $CDCl_3$) δ (ppm) 7.91 (d, *J* = 8.5 Hz, 2H), 7.45 (d, *J* = 8.5 Hz, 2H), 4.04 (s, 2H), 2.50 (s, 3H); **$^{13}$C NMR** (100 MHz, $CDCl_3$) δ (ppm) 147.0, 133.8, 130.6, 129.1, 110.7, 46.0, 22.0; **HRMS** ($ESI^+$) *m*/*z* calcd. for $C_9H_9NNaO_2S$ $[M+Na]^+$ 218.0246, found 218.0242. All data recorded were in agreement with that previously reported.[33]

**2-((2,4,6-Triisopropylphenyl)sulfonyl)acetonitrile (33)**

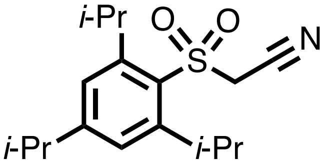

Prepared according to general procedure A using 2,4,6-triisopropylbenzenesulfonyl chloride (303 mg, 1.00 mmol) and tris(2,2′-bipyridyl)dichlororuthenium(II) hexahydrate [$Ru(bpy)_3Cl_2.6H_2O$] (7.5 mg, 0.010 mmol). The column was eluted with hexane/$Et_2O$ (8:2) to give 2-((2,4,6-triisopropylphenyl)sulfonyl)acetonitrile (**33**) as a colourless solid (234 mg, 76%).

**$R_f$** 0.21 (8:2 hexane/$Et_2O$); **M.P.** 104–105 °C; **$^1$H NMR** (400 MHz, $CDCl_3$) δ (ppm) 7.25 (s, 2H), 4.12 (s, 2H), 4.07 (septet, *J* = 7.0 Hz, 2H), 2.94 (septet, *J* = 7.0 Hz, 1H), 1.32 (d, *J* = 7.0 Hz, 12H), 1.27 (d, *J* = 7.0 Hz, 6H); **$^{13}$C NMR** (100 MHz, $CDCl_3$) δ (ppm) 155.7, 152.4, 130.2, 124.8, 110.6, 47.1, 34.5, 30.3, 25.2, 23.6; **IR** (film, $\nu_{max}/cm^{-1}$) 2966 2940, 2256, 1602, 1459, 1430, 1364, 1321, 1148, 1106, 1074; **HRMS** ($ESI^+$) *m*/*z* calcd. for $C_{17}H_{25}NNaO_2S$ $[M+Na]^+$ 330.1498, found 330.1498.

**2-((2-Iodophenyl)sulfonyl)acetonitrile (34)**

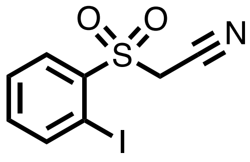

Prepared according to general procedure A using 2-iodobenzenesulfonyl chloride (302 mg, 1.00 mmol) and tris(2,2′-bipyridyl)dichlororuthenium(II) hexahydrate [$Ru(bpy)_3Cl_2.6H_2O$] (7.5 mg, 0.010 mmol). The column was eluted with hexane/EtOAc (8:2) to give 2-((2-Iodophenyl)sulfonyl)acetonitrile (**34**) as a colourless solid (270 mg, 88%).

**$R_f$** 0.20 (8:2 hexane/EtOAc); **M.P.** 97–98 °C; **$^1$H NMR** (400 MHz, $CDCl_3$) δ (ppm) 8.32 (dd, *J* = 8.0, 1.5 Hz, 1H), 8.18 (d, *J* = 8.0 Hz, 1H), 7.66 (app t, *J* = 8.0 Hz, 1H), 7.40 (app td, *J* = 8.0, 1.5 Hz, 1H), 4.48 (s, 2H); **$^{13}$C NMR** (100 MHz, $CDCl_3$) δ (ppm) 143.3, 138.9, 136.2, 133.4, 129.4, 109.9, 92.8, 42.7; **IR** (film, $\nu_{max}/cm^{-1}$) 2982, 2928, 2254, 1568, 1418, 1317, 1155, 1013; **HRMS** ($ESI^+$) *m*/*z* calcd. for $C_8H_6INNaO_2S$ $[M+Na]^+$ 329.9056, found 329.9058.

**2-((2-(Cyanomethyl)phenyl)sulfonyl)acetonitrile (35)**

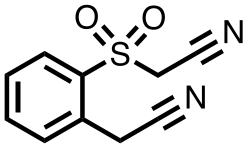

Prepared according to general procedure A using 2-((2-iodophenyl)sulfonyl)acetonitrile (**26**) (307 mg, 1.00 mmol), tris[5-fluoro-2-(2-pyridinyl-kN)phenyl-kC]iridium(III) $Ir(5\text{-}Fppy)_3$ (7.1 mg, 0.010 mmol) and the addition of *(+)*-sodium L-ascorbate (297 mg, 1.5 mmol), with the reaction run for 8 h. The column was eluted with hexane/$Et_2O$ (3:7) to give 2-((2-(cyanomethyl)phenyl)sulfonyl) acetonitrile (**35**) as a colourless solid (101 mg, 46%).

**$R_f$** 0.24 (3:7 hexane/$Et_2O$); **M.P.** 108–109 °C; **$^1$H NMR** (400 MHz, $CDCl_3$) δ (ppm) 8.19 (dd, *J* = 7.5, 1.0 Hz, 1H), 7.83 (app t, *J* = 7.5, 1.0 Hz, 1H), 7.73 (d, *J* = 7.5 Hz, 1H), 7.69 (app t, *J* = 7.5 Hz, 1H), 4.35 (s, 2H), 4.24 (s, 2H); **$^{13}$C NMR** (100 MHz, $CDCl_3$) δ (ppm) 136.6, 134.7, 132.9, 132.4, 131.0, 130.2, 117.1, 110.2, 46.4, 22.6; **IR** (film, $\nu_{max}/cm^{-1}$) 2983, 2930, 2257, 1575, 1479, 1423, 1381, 1319, 1154, 1120, 719, 525; **HRMS** ($ESI^+$) *m*/*z* calcd. for $C_{10}H_8N_2NaO_2S$ $[M+Na]^+$ 243.0199, found 243.0198.

**2-(Quinolin-3-yl)acetonitrile (36)**

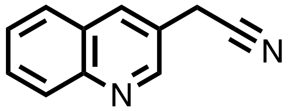

Prepared according to general procedure D using quinoline-3-diazonium tetrafluoroborate[34] (243 mg, 1.00 mmol). During extraction, sat. aq. $NaHCO_3$ (50 mL) was used in place of 1 N HCl. The column was eluted with hexane/EtOAc (1:1) to give 2-(quinolin-3-yl)acetonitrile (**36**) as an orange solid (130 mg, 80%).

**$R_f$** 0.10 (1:1 hexane/EtOAc); **$^1$H NMR** (400 MHz, $CDCl_3$) δ (ppm) 8.81 (brs, 1H), 8.19 (brs, 1H), 8.11 (d, *J* = 8.0 Hz, 1H), 7.83 (d, *J* = 8.0 Hz, 1H), 7.75 (app t, *J* = 8.0 Hz, 1H), 7.60 (app t, *J* = 8.0 Hz, 1H), 3.96 (s, 2H); **$^{13}$C NMR**

(100 MHz, $CDCl_3$) δ (ppm) 149.7, 147.7, 134.9, 130.3, 129.5, 127.8, 127.7 (2C), 123.1, 117.0, 21.6; **HRMS** ($ESI^+$) *m/z* calcd. for $C_{11}H_9N_2$ $[M+H]^+$ 169.0760, found 169.0758. All data recorded were in agreement with that previously reported.[35]

**2-(4-Nitrophenyl)acetonitrile (37)**

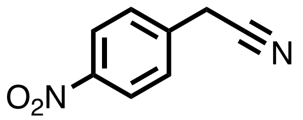

Prepared according to general procedure D using 4-nitrobenzenediazonium tetrafluoroborate[36] (237 mg, 1.00 mmol). The column was eluted with hexane/EtOAc (7:3) to give 2-(4-nitrophenyl)acetonitrile (**37**) as an orange solid (130 mg, 80%).

**$R_f$** 0.30 (6:4 hexane/EtOAc); **$^1$H NMR** (400 MHz, $CDCl_3$) δ (ppm) 8.26 (d, *J* = 8.5 Hz, 2H), 7.54 (d, *J* = 8.5 Hz, 2H), 3.89 (s, 2H); **$^{13}$C NMR** (100 MHz, $CDCl_3$) δ (ppm) 147.9, 137.1, 129.1, 124.5, 116.6, 23.7; **HRMS** ($ESI^-$) *m/z* calcd. for $C_8H_5N_2O_2$ $[M–H]^-$ 161.0357, found 161.0355. All data recorded were in agreement with that previously reported.[37]

**2-(3-Bromophenyl)acetonitrile (38)**

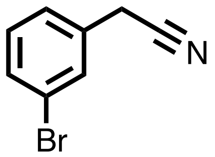

Prepared according to general procedure D using 3-bromobenzenediazonium tetrafluoroborate[38] (271 mg, 1.00 mmol). The column was eluted with hexane/EtOAc (9:1) to give diethyl 2-(3-bromophenyl)acetonitrile (**38**) as a pale orange solid (143 mg, 73%).

**$R_f$** 0.20 (9:1 hexane/EtOAc); **$^1$H NMR** (400 MHz, $CDCl_3$) δ (ppm) 7.53–7.43 (m, 2H), 7.34–7.22 (m, 2H), 3.72 (s, 2H); **$^{13}$C NMR** (100 MHz, $CDCl_3$) δ (ppm) 132.1, 131.5, 131.1, 130.8, 126.7, 123.2, 117.3, 23.4; **HRMS** ($APCI^+$) *m/z* calcd. for $C_8H_7{}^{79}BrN$ $[M+H]^+$ 195.9756, found 195.9751. All data recorded were in agreement with that previously reported.[39]

**Methyl 3-(cyanomethyl)benzoate (39)**

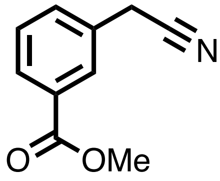

Prepared according to general procedure D using 3-(methoxycarbonyl) benzenediazonium tetrafluoroborate[40] (250 mg, 1.00 mmol). The column was eluted with hexane/EtOAc (8:2) to give methyl 3-(cyanomethyl)benzoate (**39**) as a yellow oil (113 mg, 65%).

**R$_f$** 0.18 (8:2 hexane/EtOAc); **$^1$H NMR** (400 MHz, $CDCl_3$) δ (ppm) 8.03–7.98 (m, 2H), 7.55 (d, *J* = 8.0 Hz, 1H), 7.47 (app t, *J* = 8.0 Hz, 1H), 3.93 (s, 3H), 3.81 (s, 2H); **$^{13}$C NMR** (100 MHz, $CDCl_3$) δ (ppm) 166.5, 132.4, 131.2, 130.5, 129.5, 129.2 (2C), 117.5, 52.5, 23.6; **HRMS** (ESI$^+$) *m*/*z* calcd. for $C_{10}H_9NNaO_2$ $[M+Na]^+$ 198.0525, found 198.0524. All data recorded were in agreement with that previously reported.[41]

### Cyanomethylation of pharmaceuticals

### 1,3-Dioxoisoindolin-2-yl 3-(4,5-diphenyloxazol-2-yl)propanoate

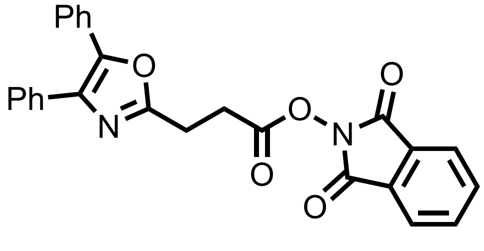


N,N´-Dicyclohexylcarbodiimide (2.06 g, 12.0 mmol) was added to a solution of oxaprozin (**40**) (3.52 g, 12.0 mmol), *N*-hydroxyphthalimide (1.63 g, 10.0 mmol) and 4-(dimethylamino)pyridine (61 mg, 0.50 mmol) in $CH_2Cl_2$ (40 mL) and the reaction stirred at rt for 16 h. The resulting mixture was filtered through Celite® washing with $CH_2Cl_2$ (100 mL), the filtrate was washed with 1 N HCl (50 mL), dried ($MgSO_4$) and concentrated under reduced pressure. The residue was purified by silica gel flash column chromatography, eluting with hexane/EtOAc (7:3) to give 1,3-dioxoisoindolin-2-yl 3-(4,5-diphenyloxazol-2-yl)propanoate as a colourless solid (3.30 g, 75%).

**R$_f$** 0.20 (7:3 hexane/EtOAc); **M.P.** 111–113 °C; **$^1$H NMR** (400 MHz, $CDCl_3$) δ (ppm) 7.93–7.87 (m, 2H), 7.82–7.77 (m, 2H), 7.67 (dd, *J* = 8.0, 1.5 Hz, 2H), 7.60 (dd, *J* = 8.0, 1.5 Hz, 2H), 7.41–7.30 (m, 6H), 3.33 (s, 4H); **$^{13}$C NMR** (100 MHz, $CDCl_3$) δ (ppm) 168.6, 161.9, 160.6, 145.9, 135.4, 135.0, 132.4, 129.0 (2C), 128.8, 128.7 (2C), 128.3, 128.1, 126.7, 124.2, 28.2, 23.2; **IR** (film, $\nu_{max}/cm^{-1}$) 3025, 1816, 1789, 1747, 1407, 1365, 1188, 1134, 1091, 963; **HRMS** (ESI$^+$) *m*/*z* calcd. for $C_{26}H_{18}N_2NaO_5$ $[M+Na]^+$ 461.1108, found 461.1115.

### 4-(4,5-Diphenyloxazol-2-yl)butanenitrile (41)

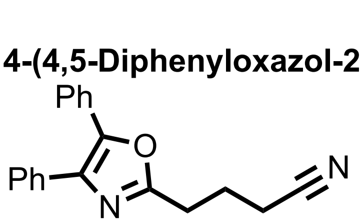


Prepared according to general procedure C using 1,3-dioxoisoindolin-2-yl 3-(4,5-diphenyloxazol-2-yl)propanoate (438 mg, 1.00 mmol) without the addition of *(+)*-sodium L-ascorbate, in anhydrous DMSO (5 mL). The column was eluted with $CH_2Cl_2/Et_2O$ (96:4) to give 4-(4,5-diphenyloxazol-2-yl)butanenitrile (**41**) as a colourless solid (182 mg, 63%).

**$R_f$** 0.26 (96:4 $CH_2Cl_2/Et_2O$); **M.P.** 67–69 °C; **$^1$H NMR** (400 MHz, $CDCl_3$) δ (ppm) 7.63 (d, *J* = 8.0 Hz, 2H), 7.58 (d, *J* = 8.0 Hz, 2H), 7.41–7.30 (m, 6H), 3.03 (t, *J* = 7.0 Hz, 2H), 2.61 (t, *J* = 7.0 Hz, 2H), 2.25 (app quintet, J = 7.0 Hz, 2H); **$^{13}$C NMR** (100 MHz, $CDCl_3$) δ (ppm) 161.3, 145.7, 135.3, 132.4, 128.9, 128.8 (3C), 128.3, 128.0, 126.6, 119.2, 26.8, 22.9, 16.8; **IR** (film, $\nu_{max}/cm^{-1}$) 3028, 2957, 2244, 1587, 1500, 1442, 1432, 1214, 1057, 757, 695; **HRMS** ($ESI^+$) *m/z* calcd. for $C_{19}H_{16}N_2NaO$ $[M+Na]^+$ 311.1155, found 311.1159.

**6-Methylthiochroman-7-sulfonyl chloride 1,1-dioxide**

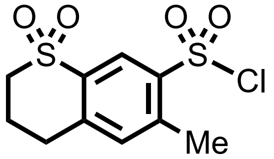

Chlorination procedure:[42] meticrane (**42**) (1.10 g, 4.00 mmol) was added to chlorosulfonic acid (6.00 mL) at 0 °C and stirred for 5 min. The reaction was then heated at 100 °C for 2 h, before cooling to rt and careful drop-wise addition to ice/water. The resulting mixture was filtered, washed with ice-cold water and the residue dried under high vacuum. The residue was purified by recyrstallisation from toluene to give 6-methylthiochroman-7-sulfonyl chloride 1,1-dioxide as colourless plates (960 mg, 81%).

**M.P.** 161–163 °C; **$^1$H NMR** (400 MHz, $CDCl_3$) δ (ppm) 8.59 (s, 1H), 7.33 (s, 1H), 3.43–3.38 (m, 2H), 3.09 (t, *J* = 6.5 Hz, 2H), 2.80 (s, 3H), 2.59–2.51 (m, 2H); **$^{13}$C NMR** (100 MHz, $CDCl_3$) δ (ppm) 144.0, 142.3, 141.9, 138.0, 134.7, 125.4, 50.6, 28.6, 20.7, 20.4; **IR** (film, $\nu_{max}/cm^{-1}$) 2941, 1597, 1391, 1288, 1171, 1140, 1126, 1087; **HRMS** ($ESI^+$) *m/z* calcd. for $C_{10}H_{12}{}^{35}ClO_4S_2$ $[M+H]^+$ 294.9860, found 294.9861.

**2-((6-Methyl-1,1-dioxidothiochroman-7-yl)sulfonyl)acetonitrile (43)**

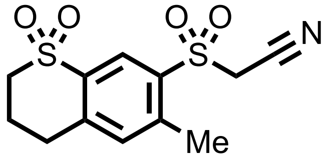

Prepared according to general procedure A using 6-methylthiochroman-7-sulfonyl chloride 1,1-dioxide (295 mg, 1.00 mmol) and tris(2,2′-bipyridyl)dichlororuthenium(II) hexahydrate [Ru(bpy)$_3$Cl$_2$.6H$_2$O] (7.5 mg, 0.010 mmol). The column was eluted with $CH_2Cl_2$/MeOH (98:2) to give 2-((6-methyl-1,1-dioxidothiochroman-7-yl)sulfonyl)acetonitrile (**43**) as a colourless solid (240 mg, 80%).

**$R_f$** 0.23 (98:2 $CH_2Cl_2$/MeOH); **M.P.** 204–206 °C; **$^1$H NMR** (400 MHz, DMSO-$d_6$) δ (ppm) 8.22 (s, 1H), 7.60 (s, 1H), 5.35 (s, 2H), 3.64–3.58 (m, 2H), 3.09 (t, *J* = 6.0 Hz, 2H), 2.68 (s, 3H), 2.39–2.31 (m, 2H); **$^{13}$C NMR** (100 MHz, DMSO-$d_6$) δ (ppm) 145.0, 142.3, 137.7, 134.9, 134.6, 125.5, 111.9, 49.4, 44.7, 27.5, 20.3, 19.9; **IR** (film, $\nu_{max}/cm^{-1}$) 2962, 2912, 2256, 1597, 1334, 1293, 1175,

1123, 979; **HRMS** (ESI$^+$) *m*/*z* calcd. for $C_{12}H_{13}NNaO_4S_2$ $[M+Na]^+$ 322.0178, found 322.0176.

**2-(4-(3-Ethyl-2,6-dioxopiperidin-3-yl)phenyl)acetonitrile (45)**

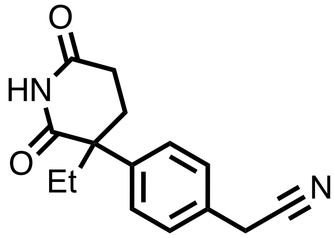


Prepared according to general procedure D using 4-(3-ethyl-2,6-dioxopiperidin-3-yl)benzenediazonium tetrafluoroborate[43] (331 mg, 1.00 mmol). The column was eluted with hexane/EtOAc (1:1) to give 2-(4-(3-ethyl-2,6-dioxopiperidin-3-yl)phenyl)acetonitrile (**45**) as a pale yellow solid (174 mg, 68%).

$\mathbf{R_f}$ 0.20 (1:1 hexane/EtOAc); **M.P.** 143–145 °C; **$^1$H NMR** (400 MHz, $CDCl_3$) δ (ppm) 8.38 (brs, 1H), 7.34 (d, *J* = 8.5 Hz, 2H), 7.29 (d, *J* = 8.5 Hz, 2 H), 3.74 (s, 2H), 2.66–2.55 (m, 1H), 2.42–2.31 (m, 2H), 2.10–1.98 (m, 1H), 0.86 (t, *J* = 7.5 Hz, 3H); **$^{13}$C NMR** (100 MHz, $CDCl_3$) δ (ppm) 175.1, 172.4, 139.0, 129.5, 128.7, 127.2, 117.7, 50.9, 32.9, 29.3, 27.0, 23.3, 9.1; **IR** (film, $\nu_{max}/cm^{-1}$) 3179, 3088, 2882, 2250, 1695, 1513, 1454, 1421, 1354, 1269, 1204; **HRMS** (ESI$^+$) *m*/*z* calcd. for $C_{15}H_{17}N_2O_2$ $[M+H]^+$ 257.1285, found 257.1283.

## Quantum Yield Measurements

The photon flux of the LED set up was determined by standard ferrioxalate actinometry,[28] following the procedure outlined previously by Yoon[44] and the calculations at λ = 455 nm by Xia.[45] Cuvettes were placed 10 cm from the LED light source.

Sample calculations:

$$mol\ Fe^{2+} = \frac{V\cdot\Delta A}{l\cdot\varepsilon} = \frac{0.0038\cdot 3.256}{1\cdot 11{,}000} = 1.115 \times 10^{-6}\ mol$$

$$photon\ flux = \frac{mol\ Fe^{2+}}{\Phi\cdot t\cdot f} = \frac{1.115 \times 10^{-6}}{0.85\cdot 30\cdot 0.9537} = 4.583 \times 10^{-8}\ \text{einstein s}^{-1}$$

Average photon flux from three experiments = $4.71 \times 10^{-8}$ einstein $s^{-1}$

A cuvette equipped with a PTFE septum and a magnetic stirrer bar was charged with 2-bromoacetophenone (40 mg, 0.20 mmol), 3-azido-2-methylbut-3-en-2-ol (**1**) (38 mg, 0.30 mmol) and tris(2,2′-bipyridyl)dichlororuthenium(II) hexahydrate [$Ru(bpy)_3Cl_2.6H_2O$] (1.5 mg, 0.002 mmol). MeCN (1.0 mL) and then 2,6-lutidine (32 mg, 35 µL, 0.30 mmol) were added and the mixture was sparged with Ar for 10 min with stirring. After sparging was complete the septum was additionally sealed with paraffin film and the reaction was irradiated with a 60 W blue LED floodlight (10 cm away) for 7 min ($1.97 \times 10^{-5}$ einstein, 0.1 eq. photons) with stirring and cooling from a small fan to maintain an ambient temperature.

Sample calculations:

$^1$H NMR yield = 18%

$$\boldsymbol{\Phi} = \frac{mol\ product}{photon\ flux\cdot t\cdot f} = \frac{3.6 \times 10^{-5}}{4.71 \times 10^{-8}\cdot 420\cdot 1.0000} = 1.8$$

Average quantum yield from three experiments = 1.8

## Cyclic voltammetry

### General procedure

Cyclic voltammetry was performed at rt on an IKA ElectraSyn 2.0 using a glassy carbon working electrode, platinum counter electrode and an Ag/AgCl (3.0 M KCl) reference electrode. A solution of substrate (0.01 M) and tetrabutylammonium hexafluorophosphate (0.10 M) in MeCN was sparged with Ar for 10 min and then analysed. The blank control was run with no analyte. Scan rates were: 100 mV/s for the blank and 2-iodobenzenesulfonyl chloride, and 200 mV/s for iodo-α-sulfonyl acetonitrile **34**. Peak potentials were measured and converted to vs SCE by subtracting 34 mV ($Ag/Ag^+ \rightarrow NHE = + 210$ mV[46], $NHE \rightarrow SCE = - 244$ mV[47]).

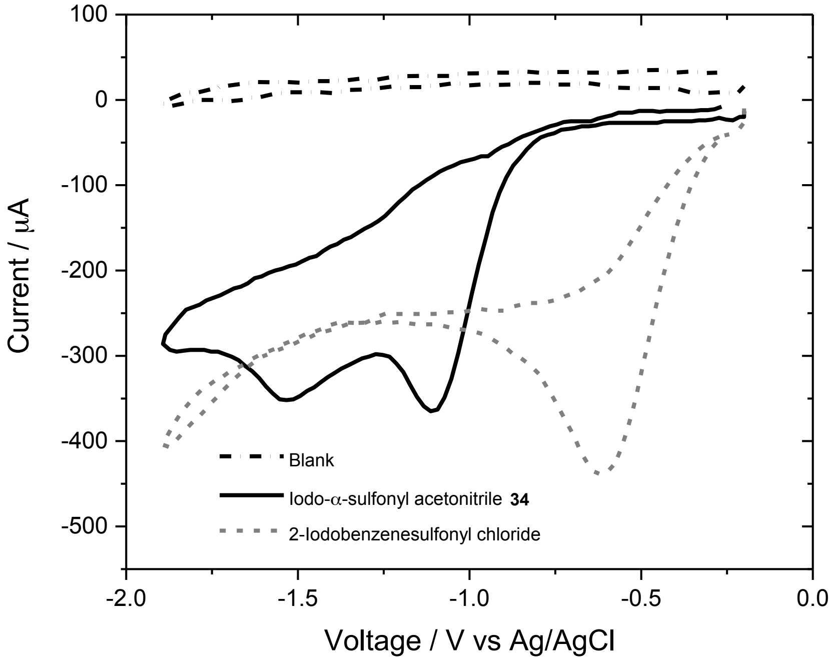

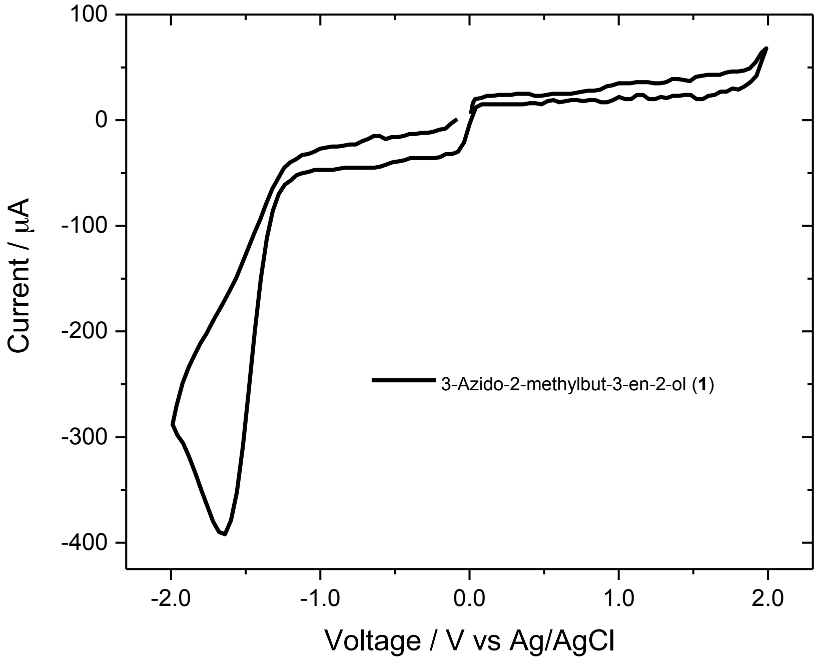
100
0
-100
-200
-300
-400
Current / μA
-2.0
-1.0
0.0
1.0
2.0
Voltage / V vs Ag/AgCl
3-Azido-2-methylbut-3-en-2-ol (1)

## References


1. Z. Liu, J. Liu, L. Zhang, P. Liao, J. Song and X. Bi, *Angew. Chem. Int. Ed.*, 2014, **53**, 5305–5309.
2. D. Dolenc and M. Harej, *J. Org. Chem.*, 2002, **67**, 312–313.
3. D. A. Guthrie, WO 2013 059194, A1, Johns Hopkins University, USA.
4. D. B. Ramachary, C. Venkaiah, Y. V. Reddy and M. Kishor, *Org. Biomol. Chem.*, 2009, **7**, 2053–2062.
5. L. Chenneberg, A. Baralle, M. Daniel, L. Fensterbank, J.-P. Goddard and C. Ollivier, *Adv. Synth. Catal.*, 2014, **356**, 2756–2762.
6. Y. Wei, G. Qiu, S. Zheng, B. Lei, Y. Yu and Y. Chen, US 20170029423, A1, Sichuan Haisco Pharmaceutical Co. Ltd. China.
7. W. P. Ratchford and C. H. Fisher, *J. Org. Chem.*, 1950, **15**, 317–325.
8. M. Lesieur, C. Genicot and P. Pasau, *Org. Lett.*, 2018, **20**, 1987–1990.
9. G. Wang, L. Chen, X. Cai, Z. Li and M. Luo, *Synlett*, 2016, **27**, 309–312.
10. X. Marset, G. Guillena and D. J. Ramón, *Chem. Eur. J.*, 2017, **23**, 10522–10526.
11. P. A. Reddy, B. C. H. Hsaing, T. N. Latifi, M. W. Hill, K. E. Woodward, S. M. Rothman, J. A. Ferrendelli and D. F. Covey, *J. Med. Chem.*, 1996, **39**, 1898–1906.
12. J. Schwarz and B. König, *Green Chem.*, 2016, **18**, 4743–4749.
13. I. Dutta, S. Yadav, A. Sarbajna, S. De, M. Hölscher, W. Leitner and J. K. Bera, *J. Am. Chem. Soc.*, 2018, **140**, 8662–8666.
14. M. C. Sheikh, S. Takagi, M Sakai, T. Mori, N. Hayashi, T. Fujie, S. Ono, T. Yoshimura and H. Morita, *Org. Biomol. Chem.*, 2011, **9**, 1244–1254.
15. T. Shen, T. Wang, C. Qin and N. Jiao, *Angew. Chem. Int. Ed.*, 2013, **52**, 6677–6680.
16. G.-Z. Wang, R. Shang and Y. Fu, *Org. Lett.*, 2018, **20**, 888–891.
17. T. Wang and N. Jiao, *J. Am. Chem. Soc.*, 2013, **135**, 11692–11695.
18. H. Li, C. P. Breen, H. Seo, T. F. Jamison, Y.-Q. Fang and M. M. Bio, *Org. Lett.*, 2018, **20**, 1338–1341.
19. A. H. G. Siebum, R. K. F. Tsang, R. van der Steen, J. Raap and J. Lugtenburg, *Eur. J. Org. Chem.*, 2004, 4391–4396.
20. J. Wang, M. Shang, H. Lundberg, K. S. Feu, S. J. Hecker, T. Qin, D. G. Blackmond and P. S. Baran, *ACS Catal.*, 2018, **8**, 9537–9542.
21. L. Bärfacker, T. Müller, A. Mengel, M. Hitchcock, A. Cleve, H. Briem, G. Siemeister, W. Bone, A. E. Fernandez-Montalvan, J. Schröder and U. Mönning, WO 2016041925 A1, Bayer Pharma Aktiengesellschaft, Germany.
22. A. Arrieta, T. García, J. M. Lago and C. Palomo, *Synth. Commun.*, 1983, **13**, 471–487.
23. P. B. S. Dawadi and J. Lugtenburg, *Eur. J. Org. Chem.*, 2008, 2288–2292.
24. G. Strappaveccia, L. Bianchi, S. Ziarelli, S. Santoro, D. Lanari, F. Pizzo and L. Vaccaro, *Org. Biomol. Chem.*, 2016, **14**, 3521–3525.
25. L. Yu, M.-L. Tang, C.-M. Si, Z. Meng, Y. Liang, J. Han and X. Sun, *Org. Lett.*, 2018, **20**, 4579–4583.

26. E. Forcellini, S. Boutin, C.-A. Lefebvre, E. E. Shayhidin, M.-C. Boulanger, G. Rhéaume, X. Barbeau, P. Lagüe, P. Mathieu and J.-F. Paquin, *Eur. J. Med. Chem.*, 2018, **147**, 130–149.
27. F. Toriyama, J. Cornella, L. Wimmer, T.-G. Chen, D. D. Dixon, G. Creech and P. S. Baran, *J. Am. Chem. Soc.*, 2016, **138**, 11132–11135.
28. T. J. Fyfe, B. Zarzycka, H. D. Lim, B. Kellam, S. N. Mistry, V. Katrich, P. J. Scammells, J. R. Lane and Ben Capuano, *J. Med. Chem.,* 2019*,* **62***,* 174–206.17.
29. A. Fawcett, J. Pradeilles, Y. Wang, T. Mutsuga, E. L. Myers and V. K. Aggarwal, *Science*, 2017, **357**, 283–286.
30. W. Xue and M. Oestreich, *Angew. Chem. Int. Ed.*, 2017, **56**, 11649–11652.
31. C. K. Mahato, S. Mukherjee, M. Kundu and A. Pramanik, *J. Org. Chem.*, 2019, **84**, 1053–1063.
32. G. Barker, R. Davenport, R. Downham, W. Farnaby, A. Goldby, D. Hannah, D. Harrisonn and H. Willems, WO 2015198045 A1, Takeda Pharmaceutical Company Ltd, Japan.
33. L. Ma, L. Yuan, C. Xu, G. Li, M. Tao and W. Zhang, *Synthesis*, 2013, **45**, 45–52.
34. G. Danoun, B. Bayarmagnai, M. F. Grünberg and L. J. Gooßen, *Angew. Chem. Int. Ed.*, 2013, **52**, 7972–7975.
35. Z.-Z. Ma, Y. Hano, T. Nomura and Y.-J. Chen, *Heterocycles*, 1999, **51**, 1883–1889.
36. H. Bonin, D. Delbrayelle, P. Demonchaux and E. Gras, *Chem. Commun.*, 2010, **46**, 2677–2679.
37. M. Nambo, M. Yar, J. D. Smith and C. M. Crudden, *Org. Lett.*, 2015, **17**, 50–53.
38. G. Wu, Y. Deng, C. Wu, Y. Zhang and J. Wang, *Angew. Chem. Int. Ed.*, 2014, **53**, 10510–10514.
39. C. Hejesen, L. K. Petersen, N. J. V. Hansenb and K. V. Gothelf, *Org. Biomol. Chem.*, 2013, **11**, 2493–2497.
40. N. Fontán, P. García-Domínguez, R. Álvarez and Á. R. de Lera, *Bioorg. Med. Chem. Lett.*, 2013, **21**, 2056–2067.
41. F. C. Novello (Merck & Co., Inc.), US3951967, 1976, A.
42. L. Chen, D. R. Sanchez, B. Zhang and B. P. Carrow, *J. Am. Chem. Soc.*, 2017, **139**, 12418-12421.
43. Montalti, M. Credi, A.; Prodi, L.; Gandolfi, M. *Handbook of Photochemistry*, 3rd ed.; CRC/Taylor & Francis: Boca Raton, FL, 2006.
44. M. A. Cismesia and T. P. Yoon, *Chem. Sci.*, 2015, **6**, 5426–5434
45. K. Liang, N. Li, Y. Zhang, T. Li, and C. Xia, *Chem. Sci.*, 2019, **10**, 3049–3053.
46. E. P. Friis, J. E. T. Andersen, L. L. Madsen, N. Bonander, P. Møller and J. Ulstrup, *Electrochim. Acta*, 1998, **43**, 1114-1122.
47. D. T. Sawyer, A. Sobkowiak and J. L. Roberts Jr. in *Electrochemistry for Chemists*, *Second Edition*, John Wiley and Sons, Inc. New York, 1995, pp. 184-204.

## NMR Spectra

## 3-Azido-2-methylbut-3-en-2-ol (1)

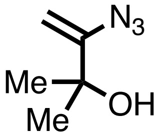


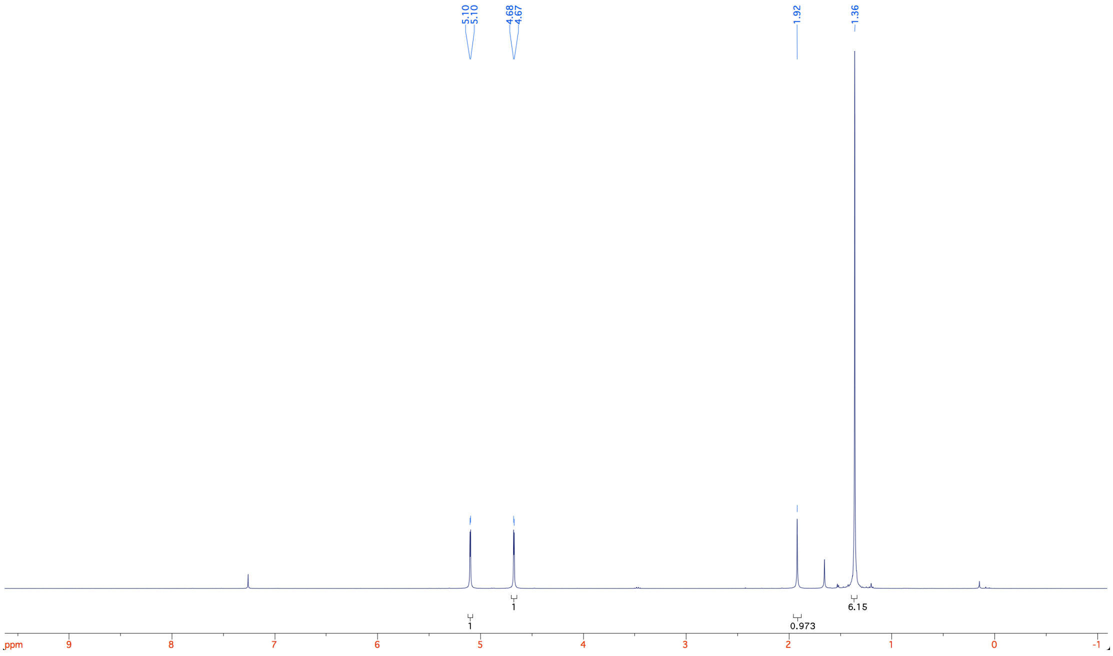


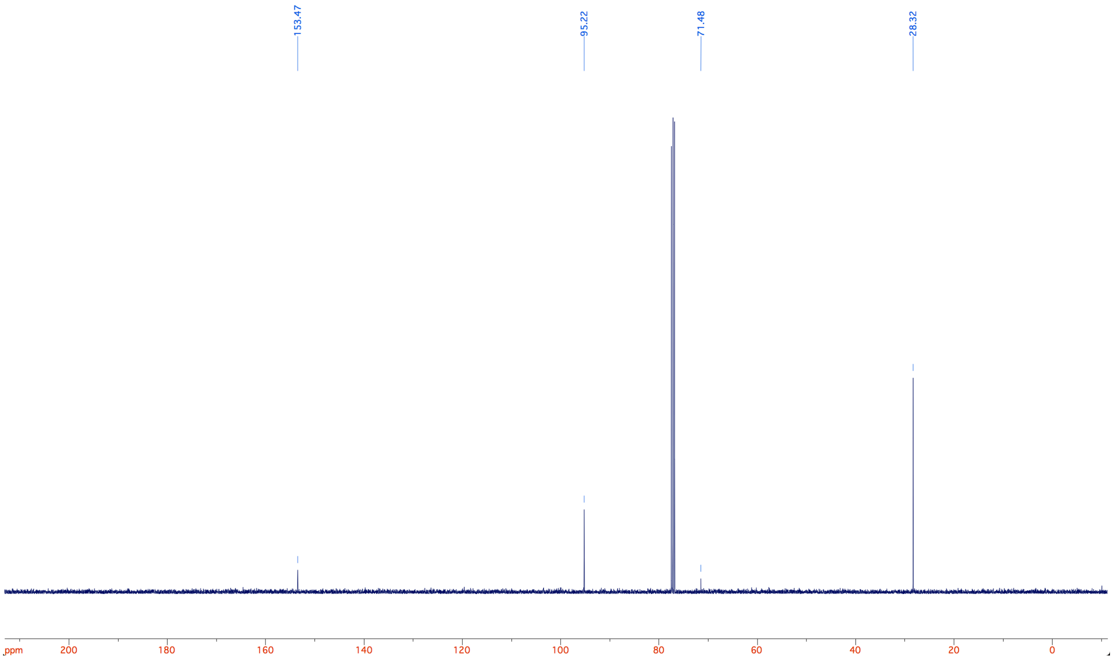

## 2-Azido-1,1-diphenylprop-2-en-1-ol (7)

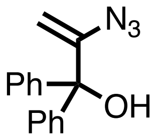


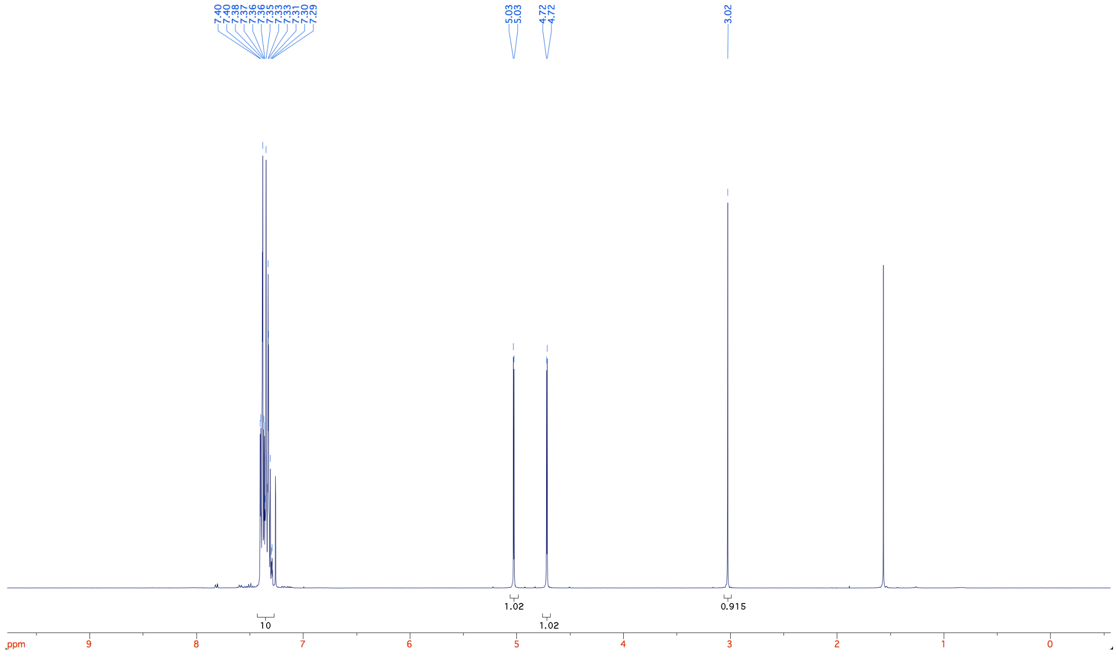


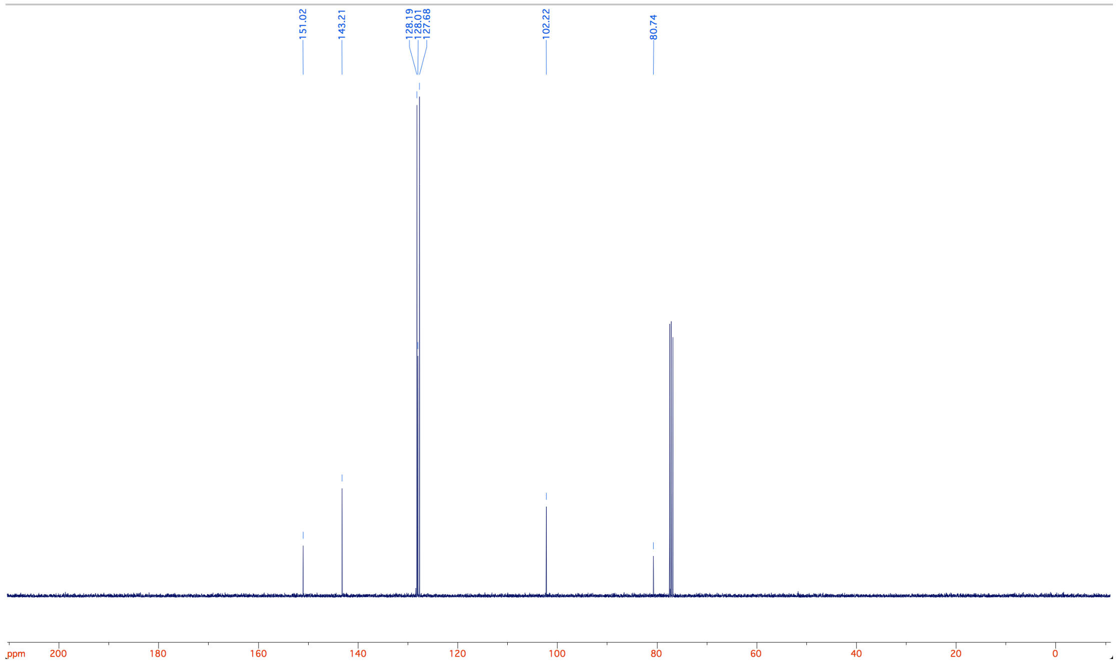

**(±)-2-Bromo-3-oxo-3-phenylpropyl acetate**

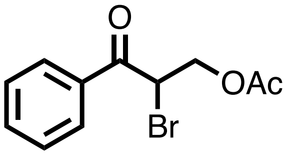


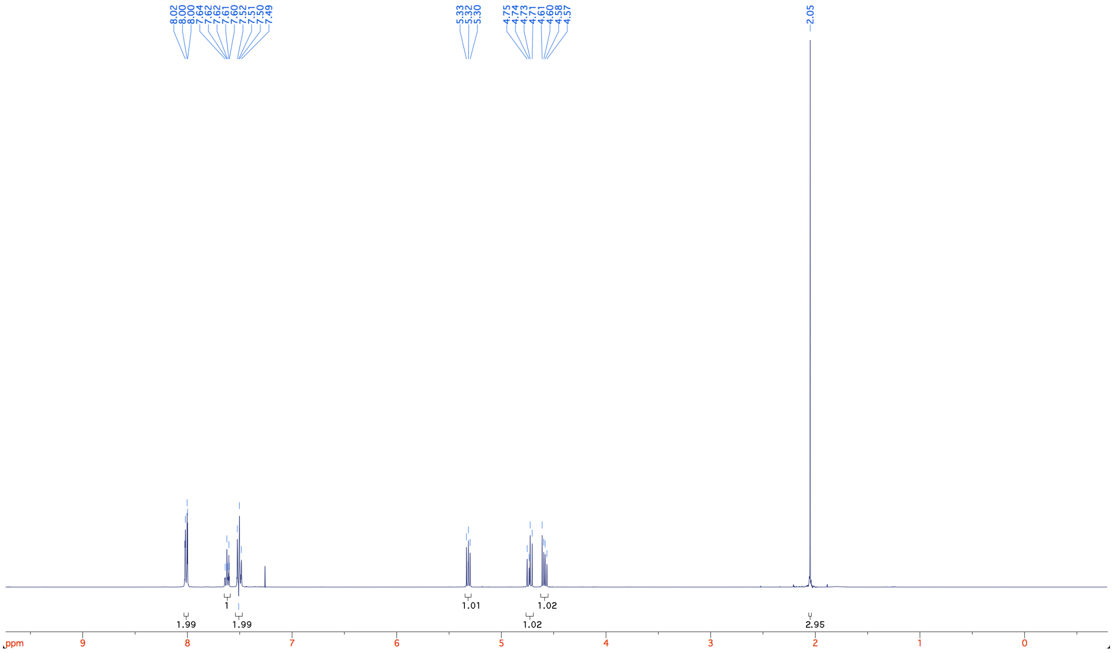


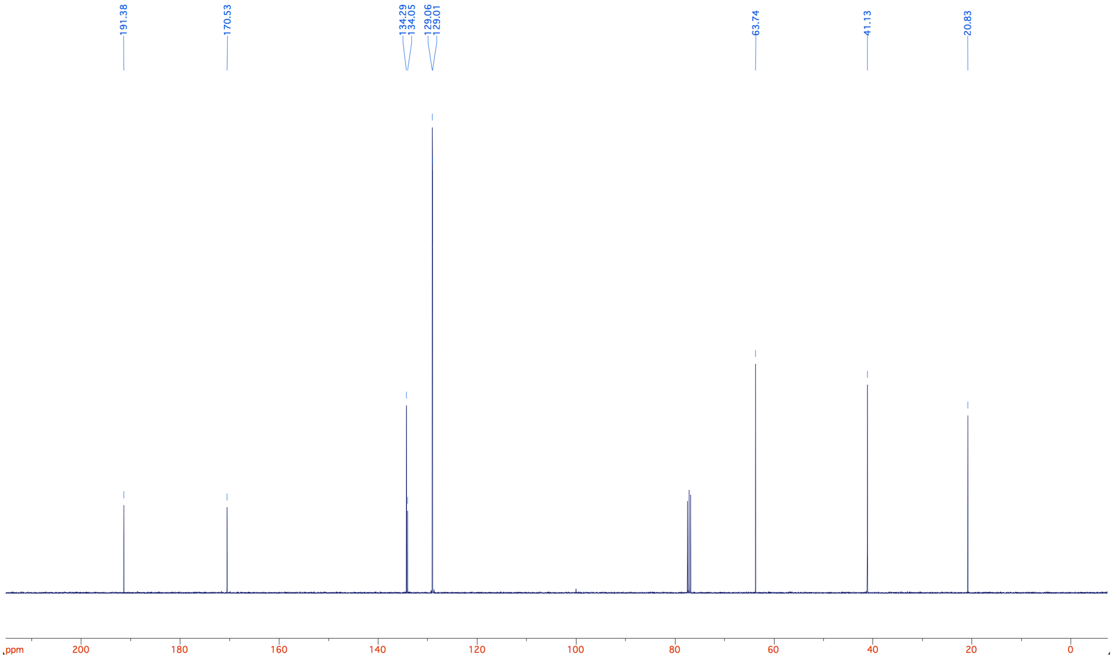

## 5-Benzyl-5-bromo-1,3-dimethylpyrimidine-2,4,6(1*H*,3*H*,5*H*)-trione

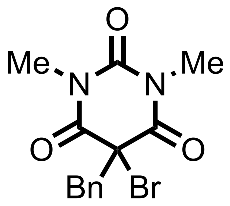


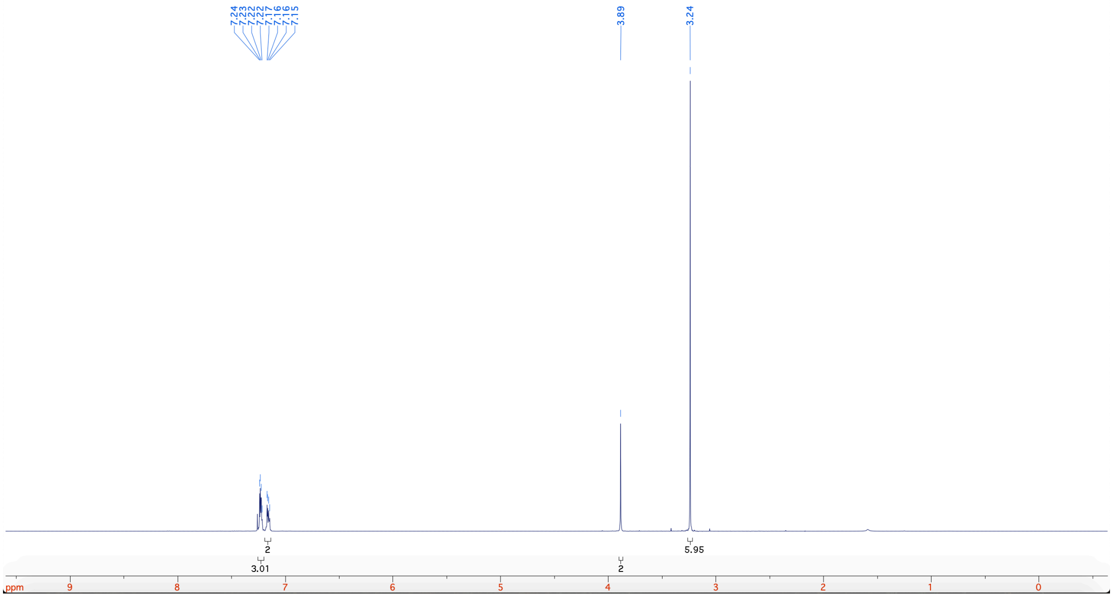


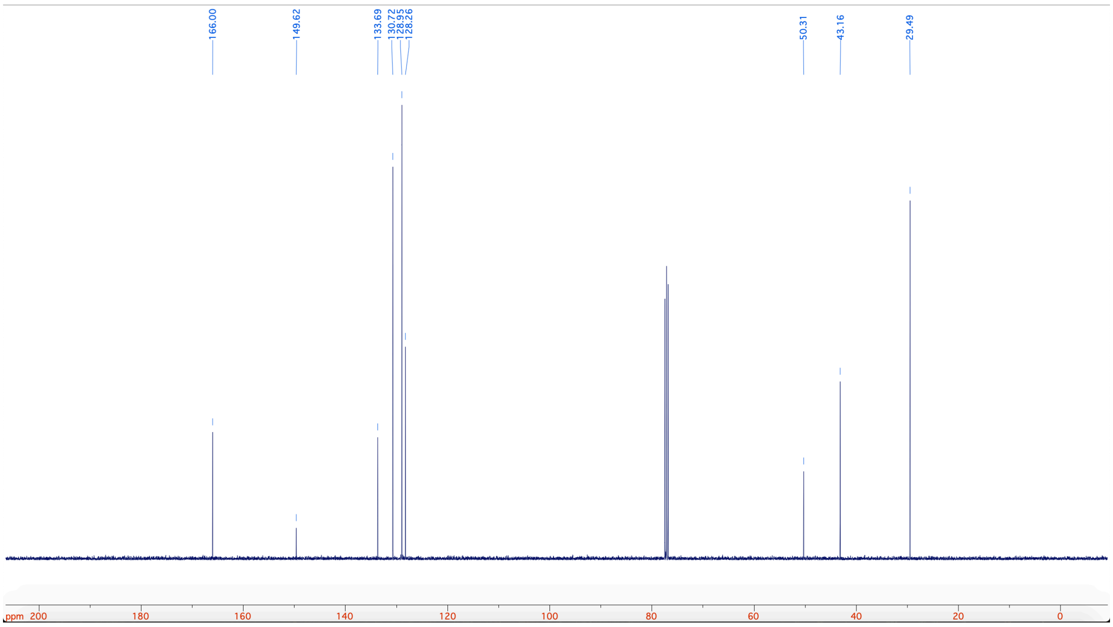

## (±)-Methyl 2-((1*H*-imidazole-1-carbonothioyl)oxy)-3-phenylpropanoate

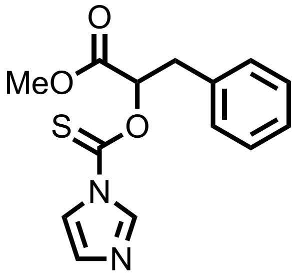


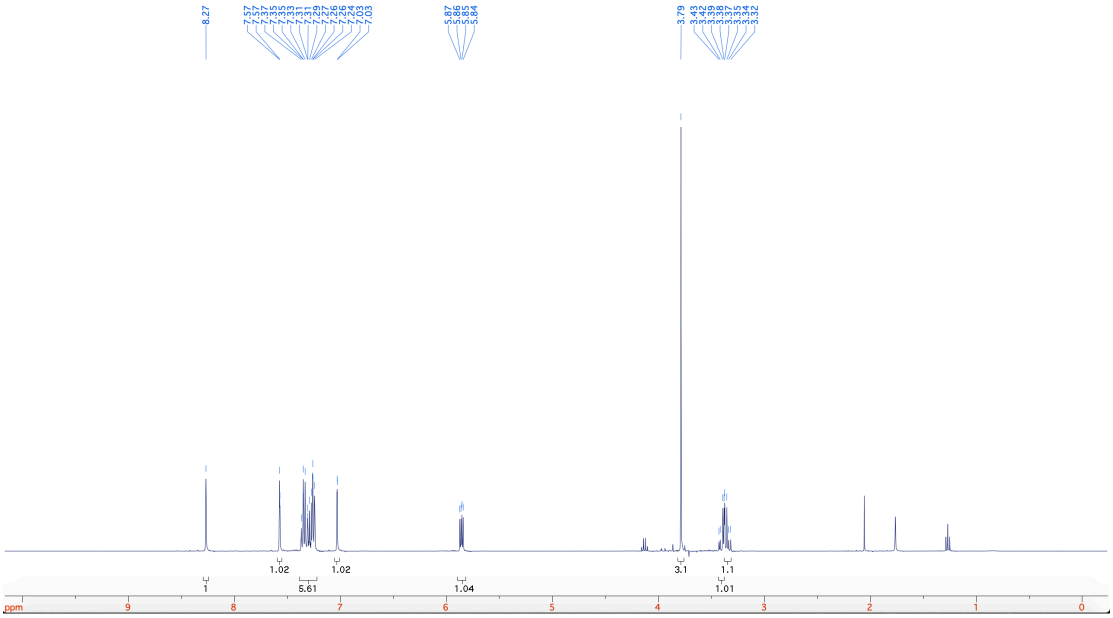


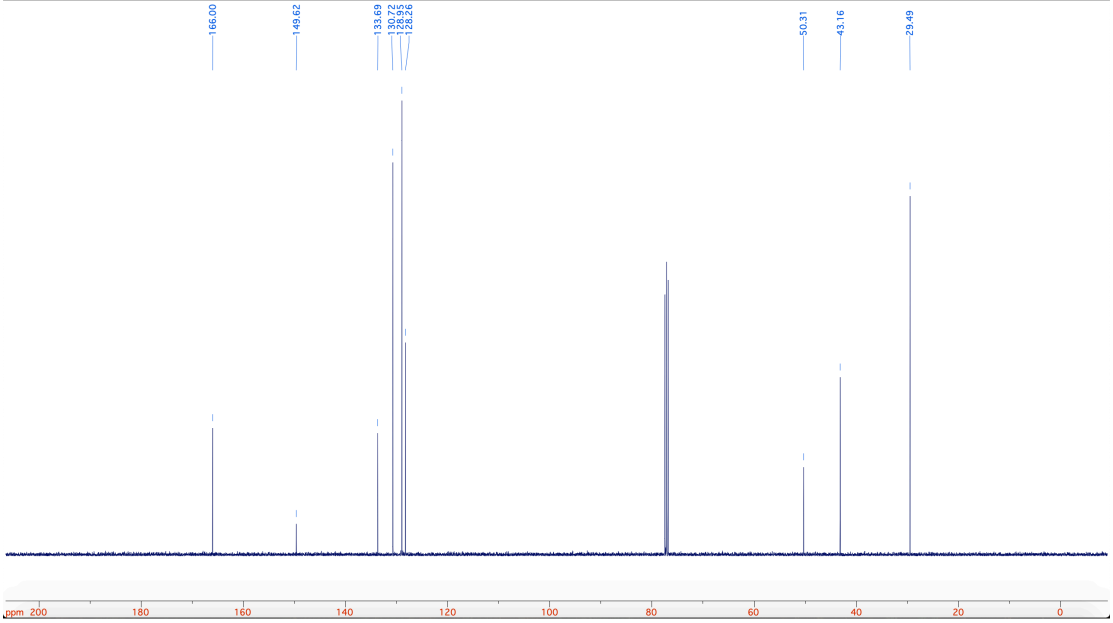

### (±)-*O*-(1-Morpholino-1-oxopropan-2-yl) 1*H*-imidazole-1-carbothioate

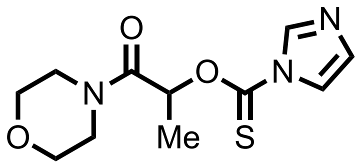


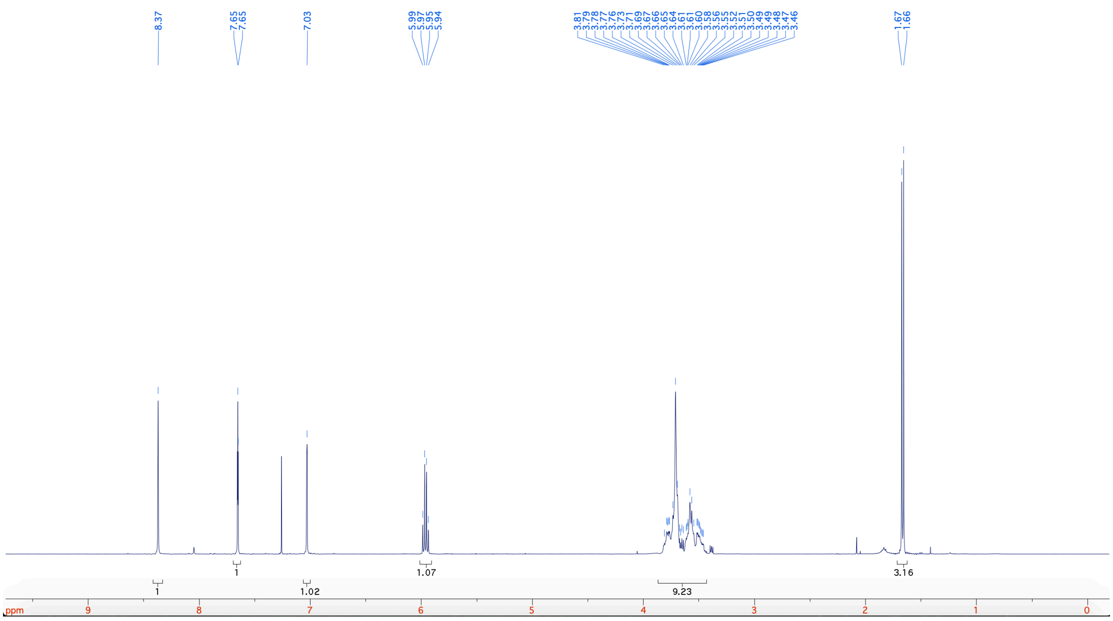


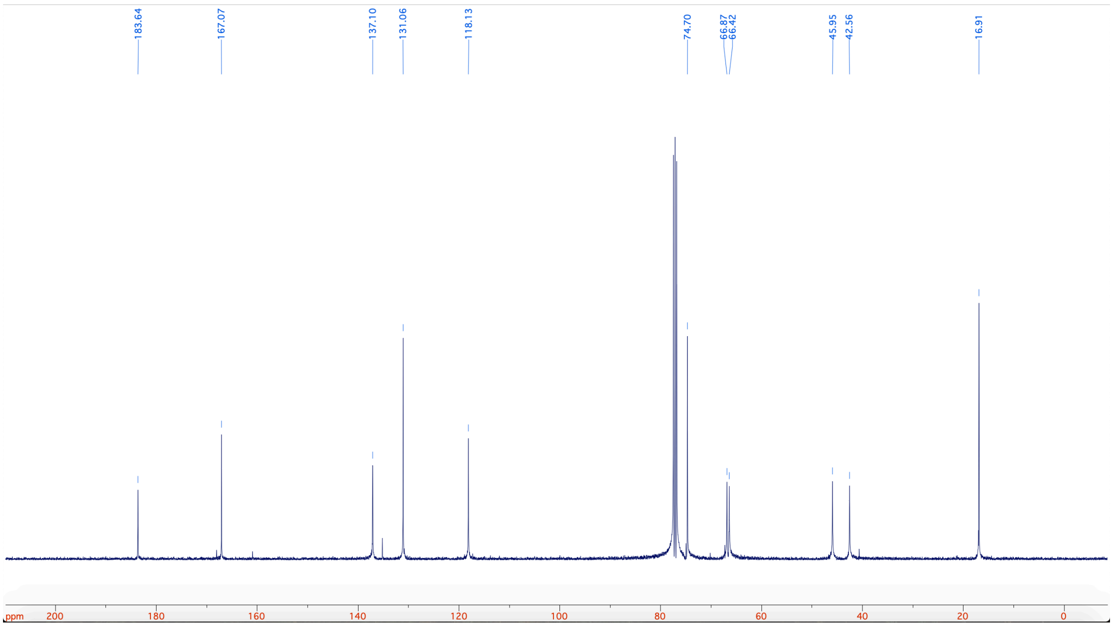

**_O_-((6-Bromopyridin-2-yl)methyl) 1_H_-imidazole-1-carbothioate**

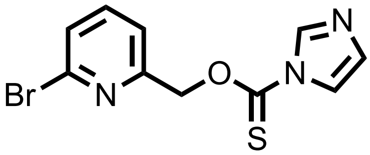


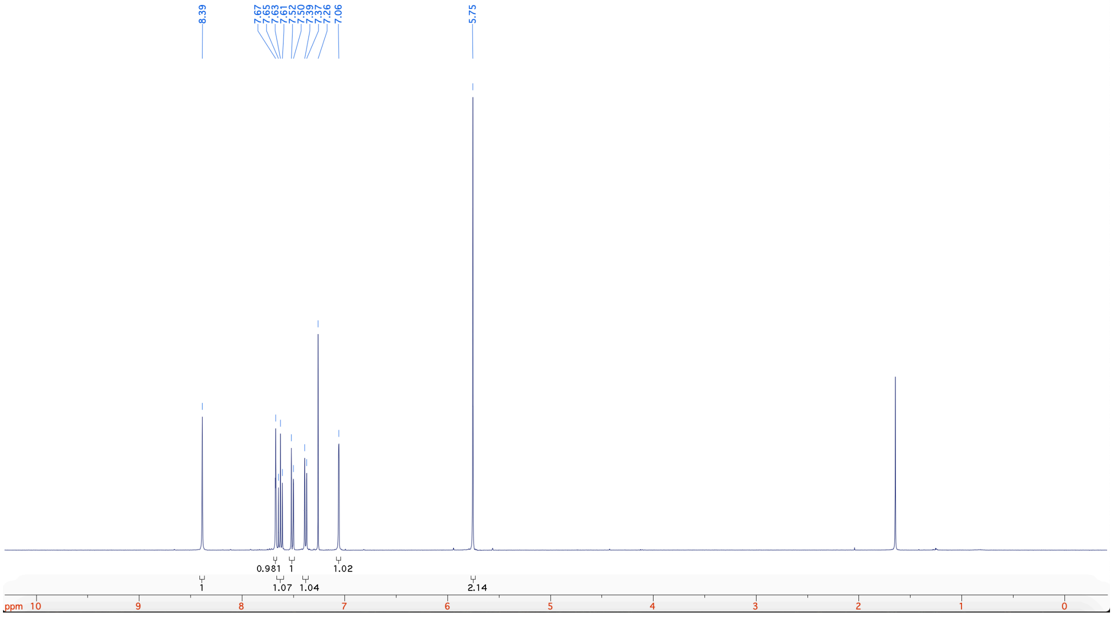


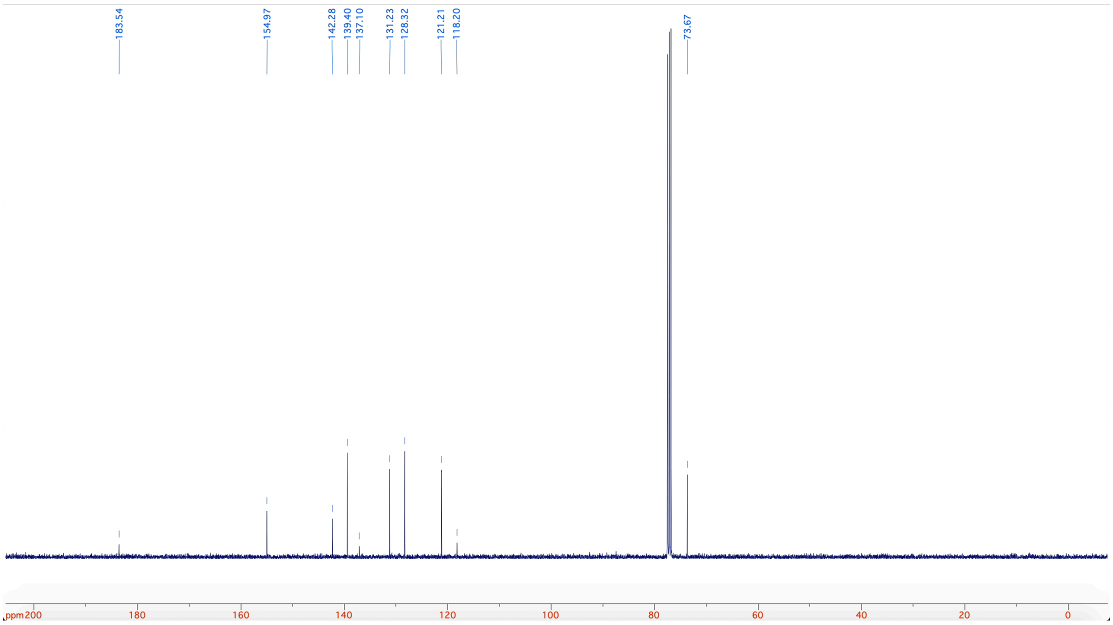

## *O*-(Pyrazin-2-ylmethyl) 1*H*-imidazole-1-carbothioate

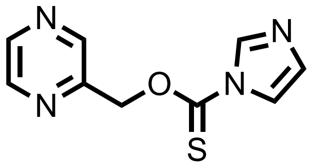

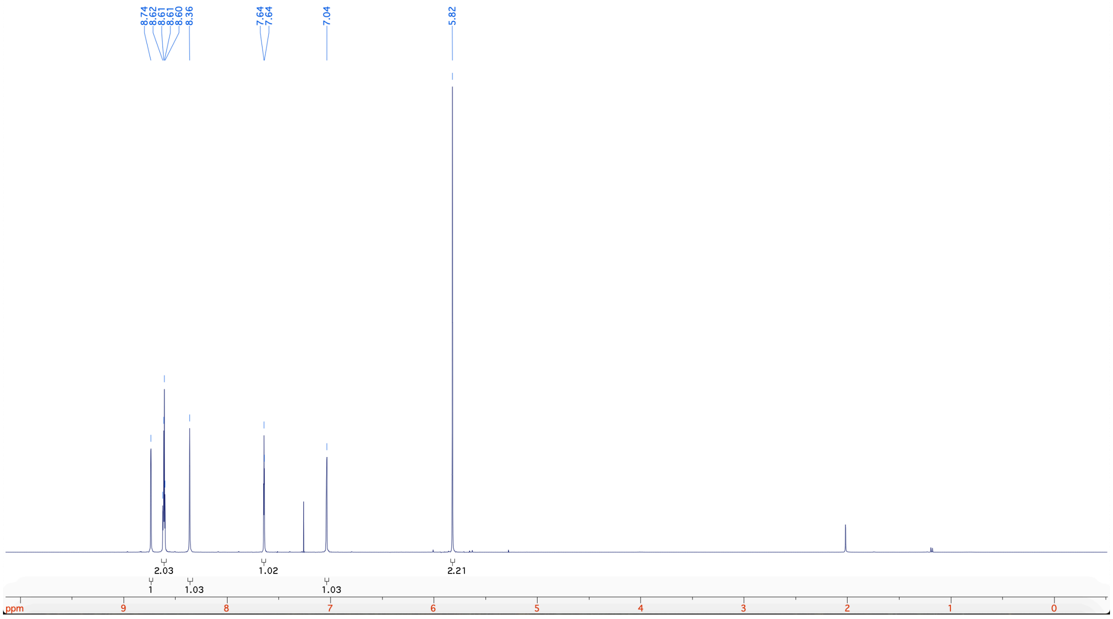


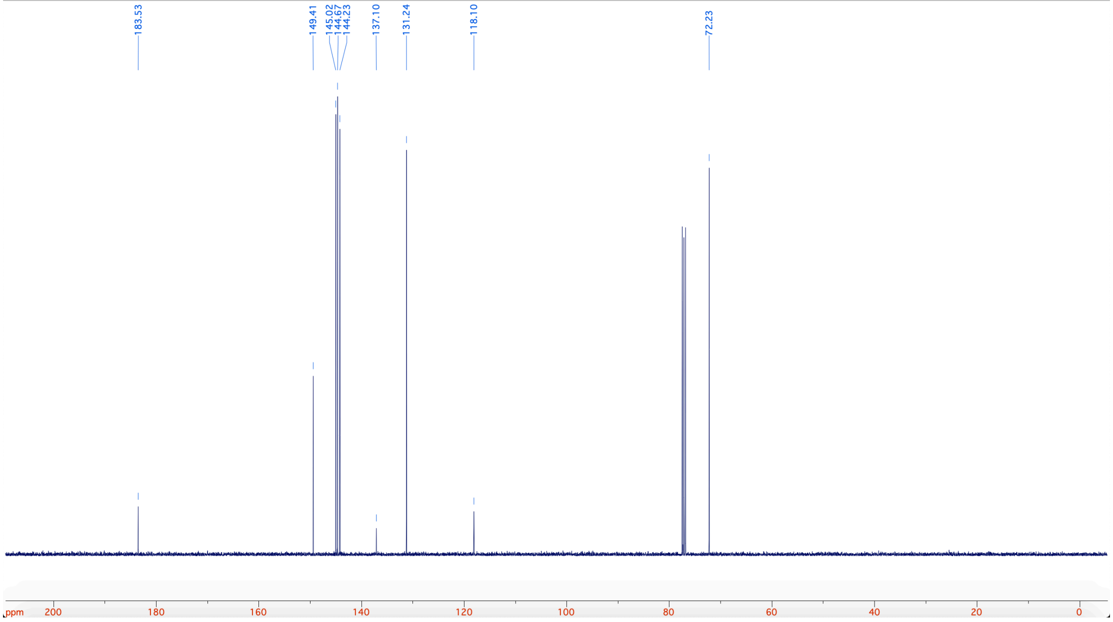

**1,3-Dioxoisoindolin-2-yl 2-(diethoxyphosphoryl)acetate**

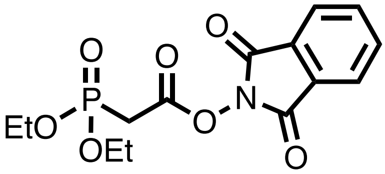


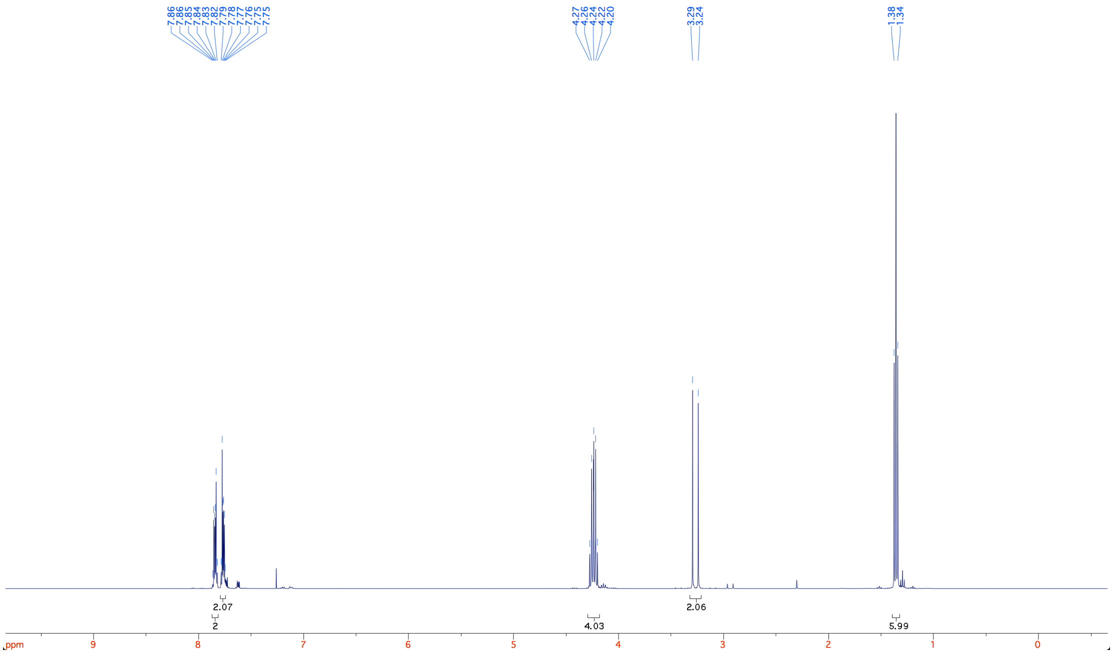


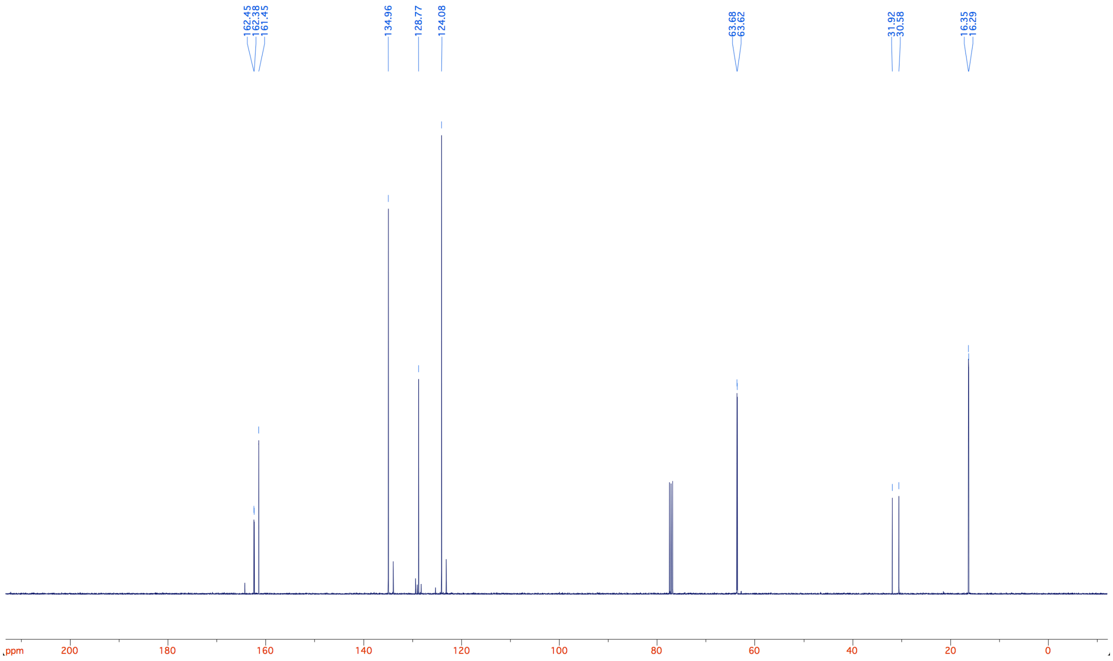

## 4-Oxo-4-phenylbutanenitrile (9)

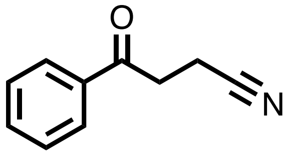


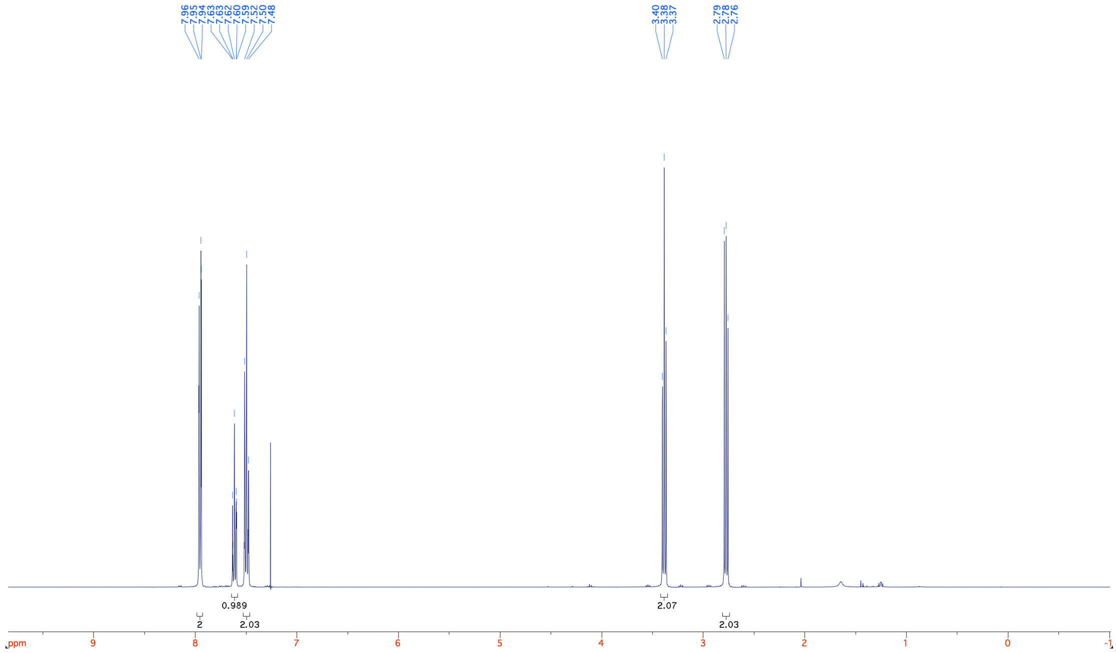


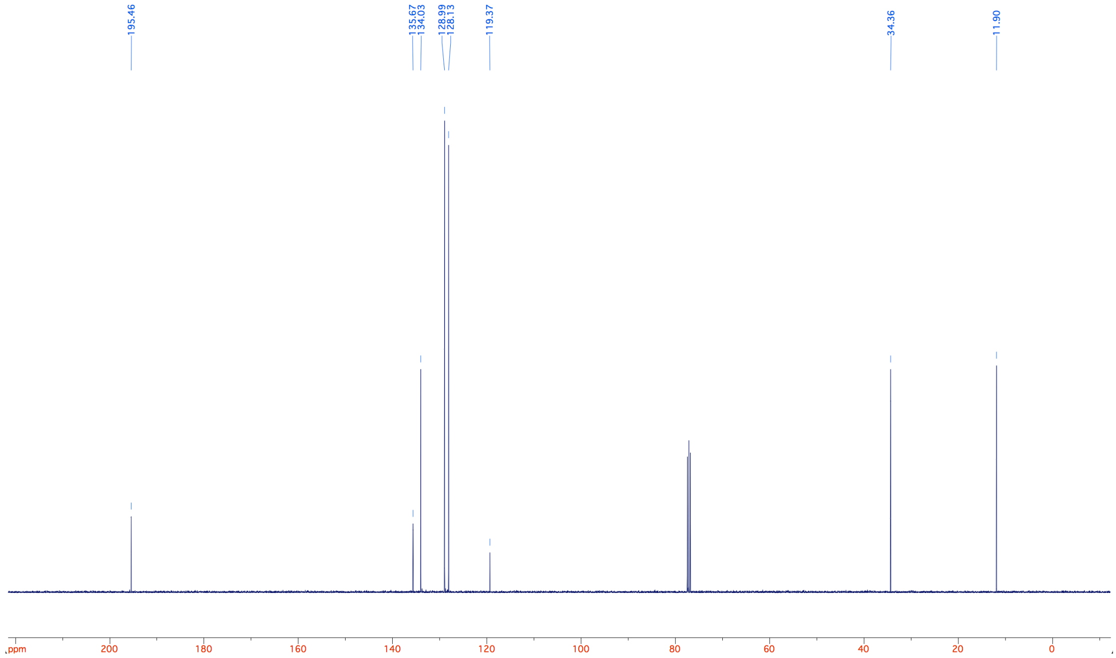

## (±)-2-(Cyanomethyl)-3-oxo-3-phenylpropyl acetate (10)

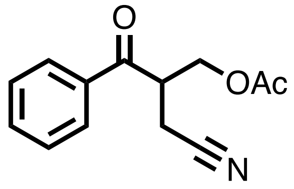


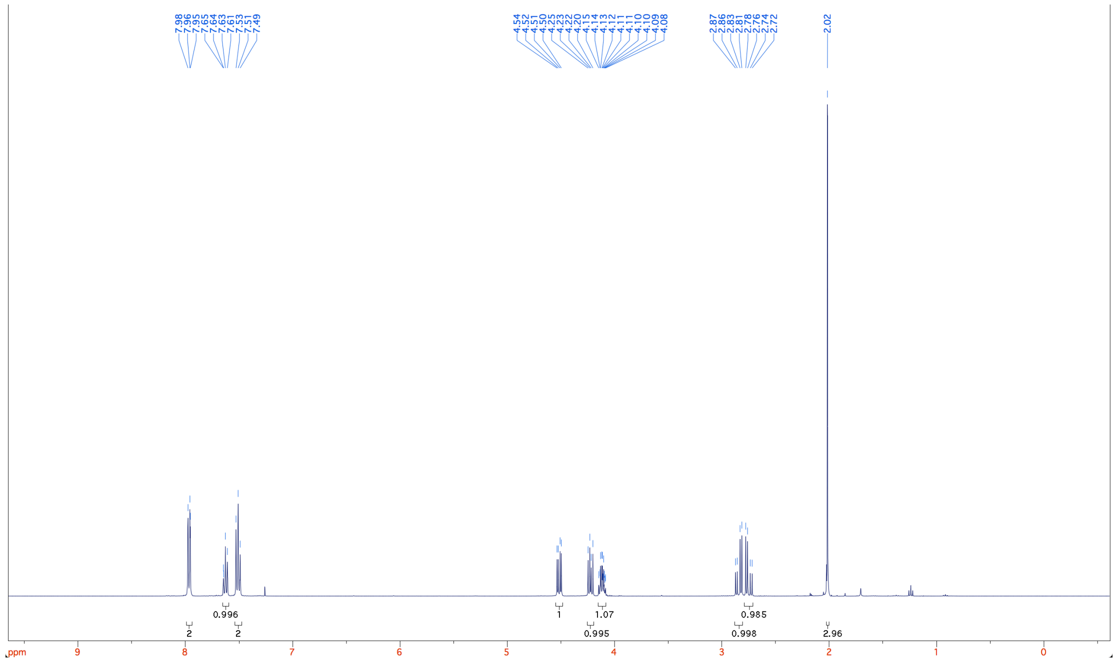


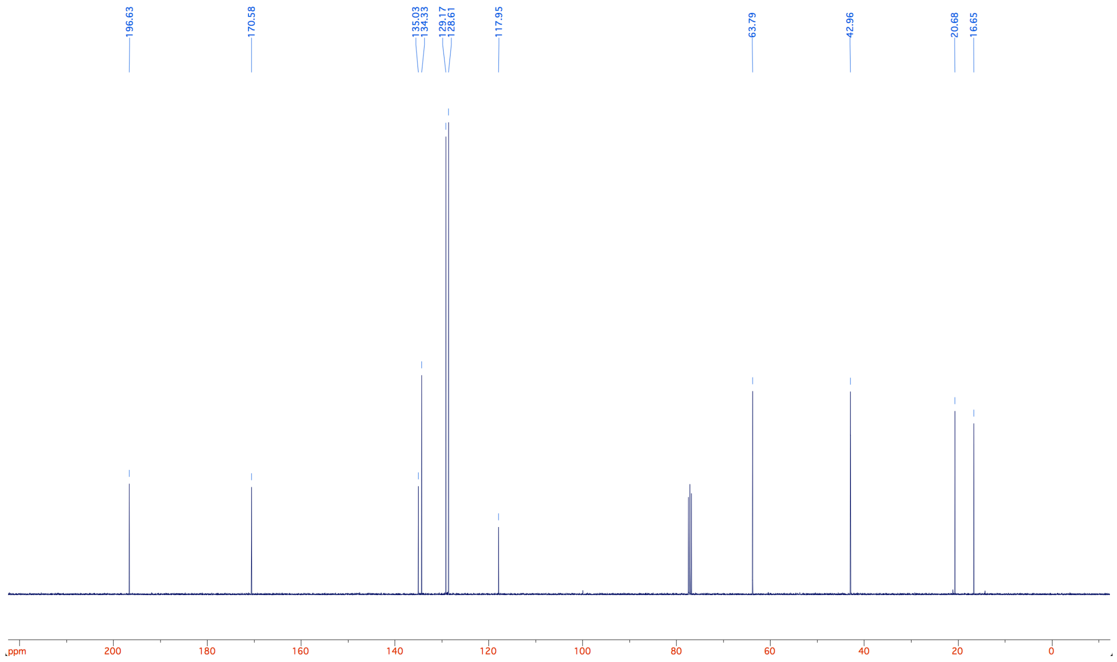

## Benzyl 3-cyanopropanoate (11)

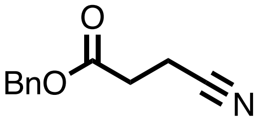


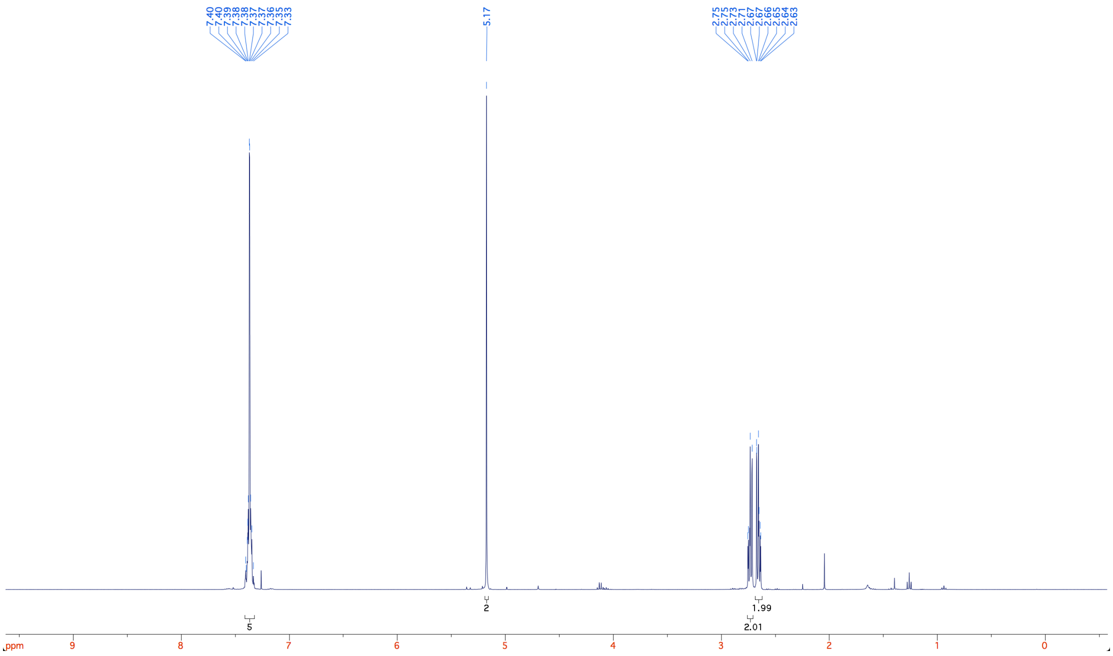


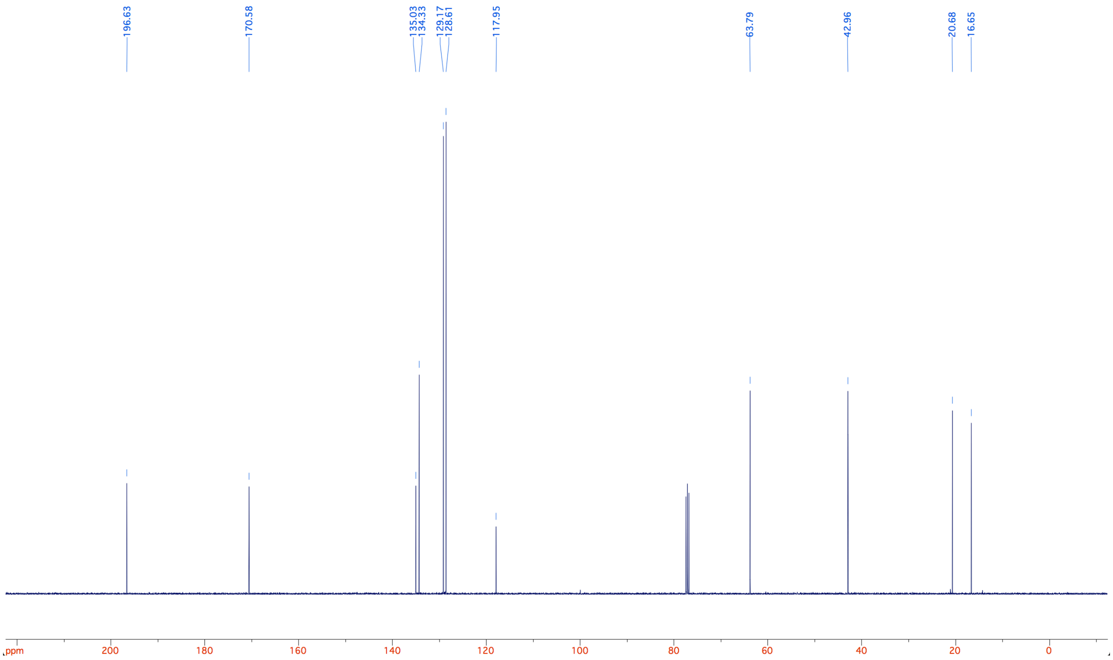

## Diethyl 2-(cyanomethyl)-2-methylmalonate (12)

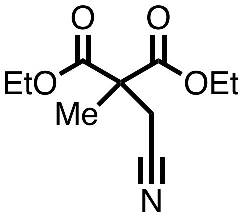


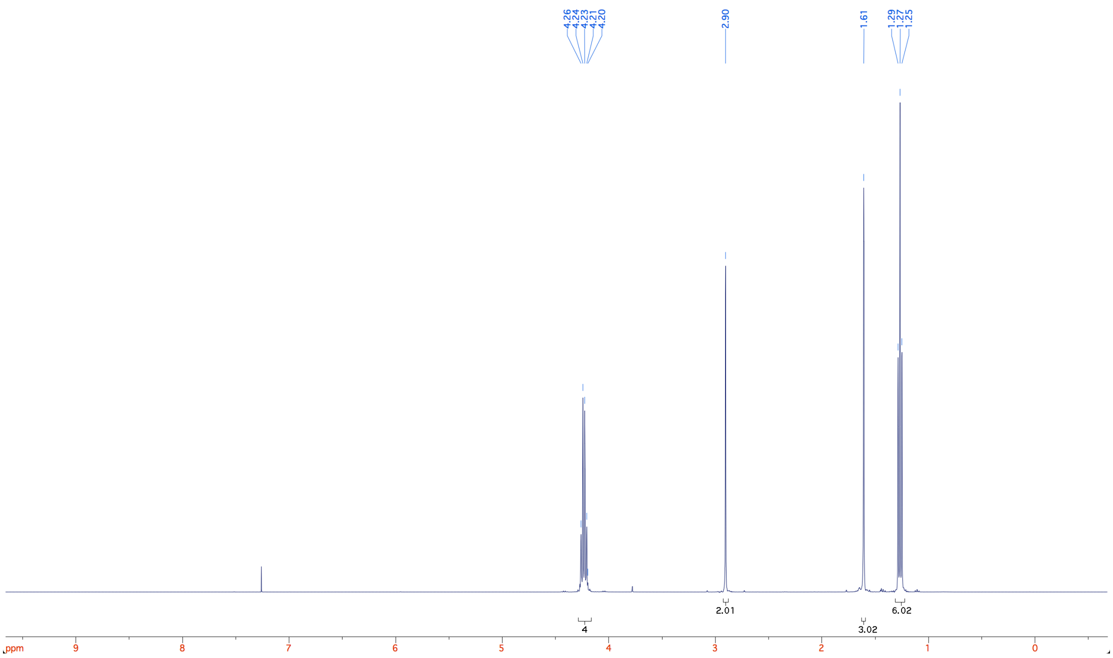


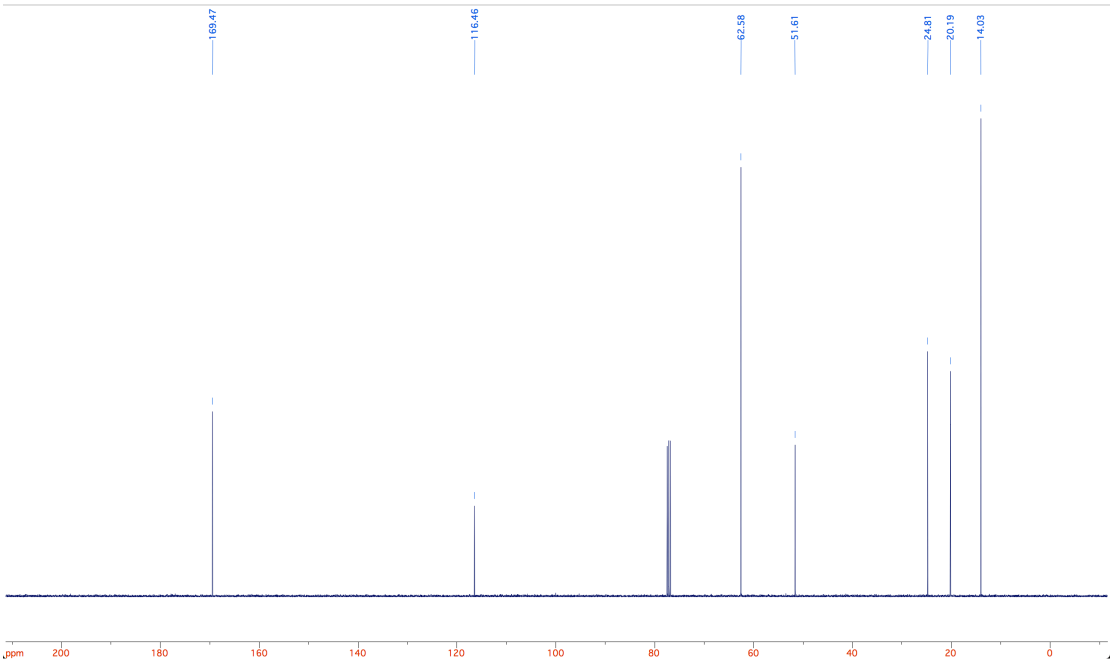

## 2-(5-Benzyl-1,3-dimethyl-2,4,6-trioxohexahydropyrimidin-5-yl)acetonitrile (13)

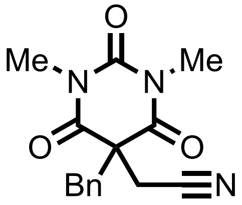


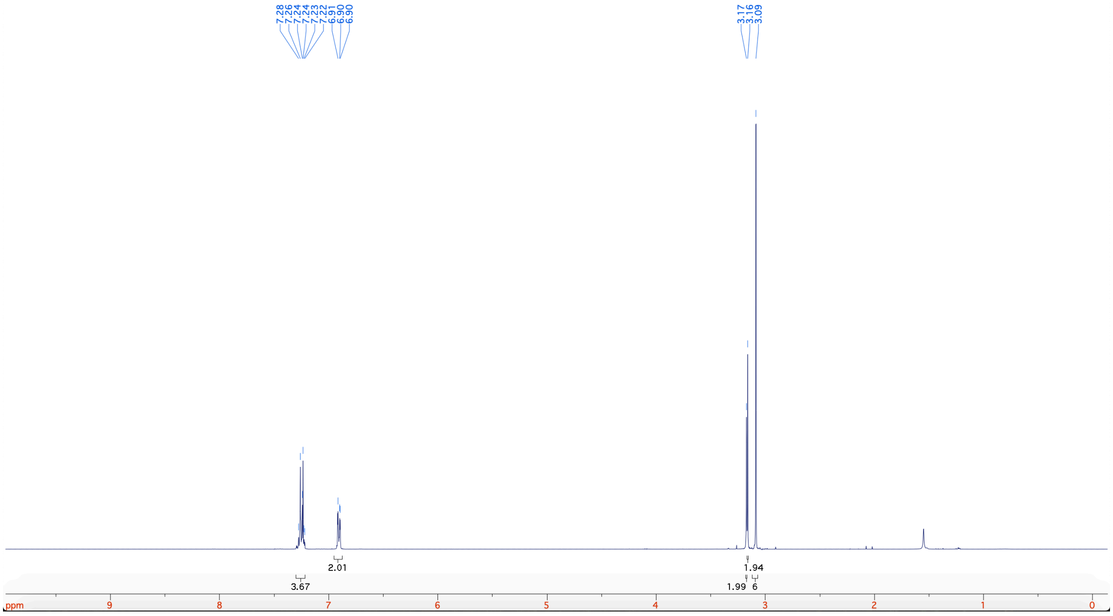


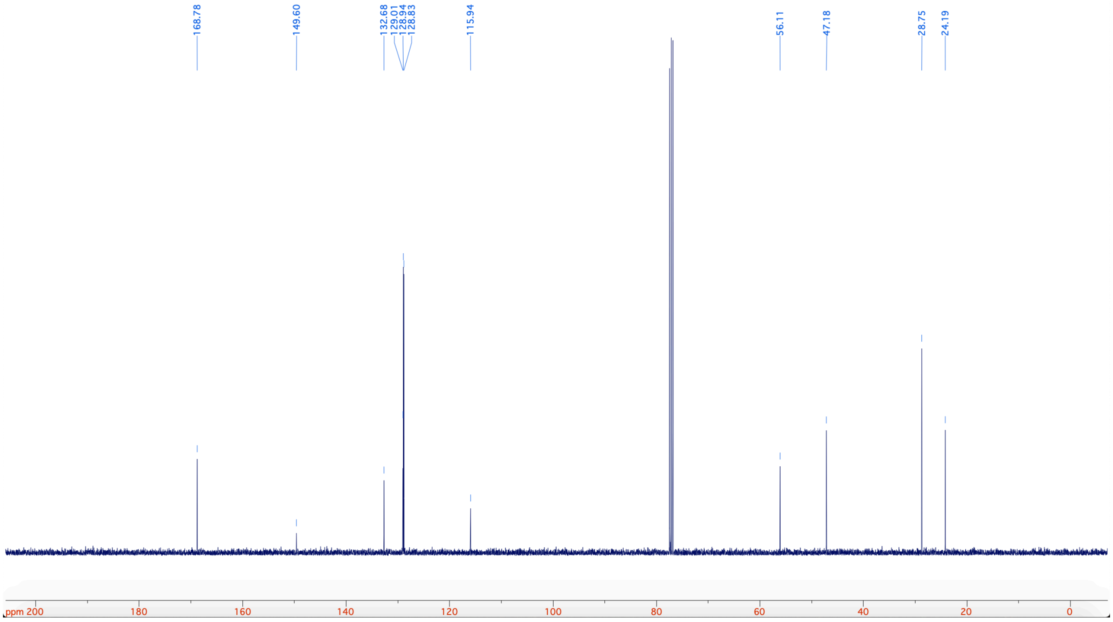

## 3-(Phenylsulfonyl)propanenitrile (14)

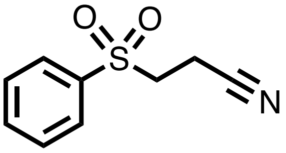


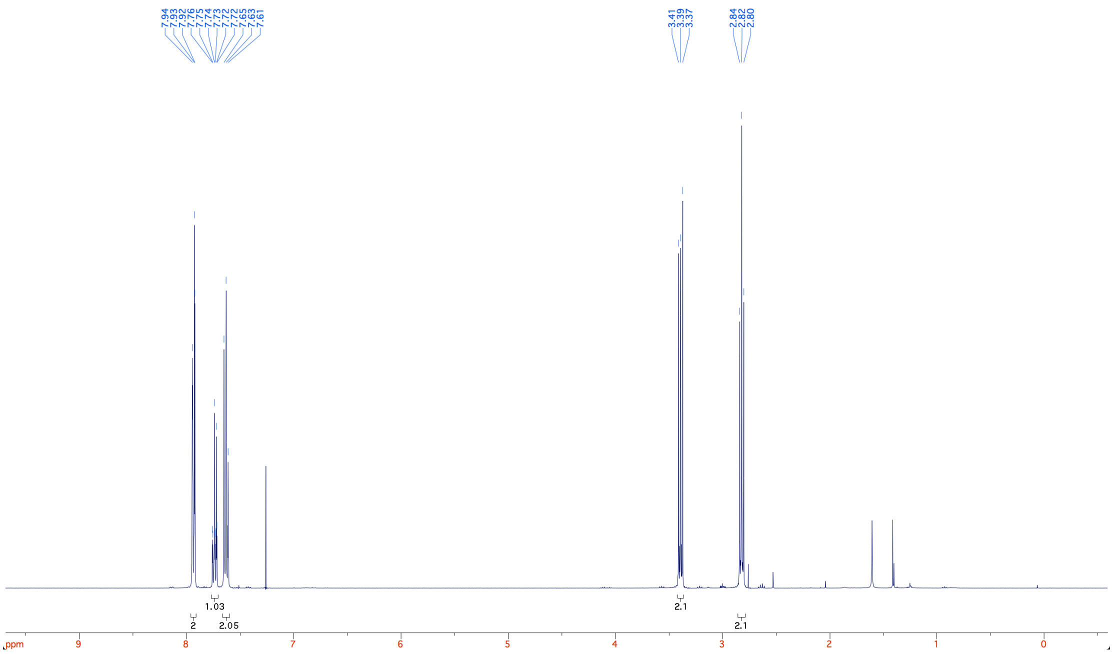


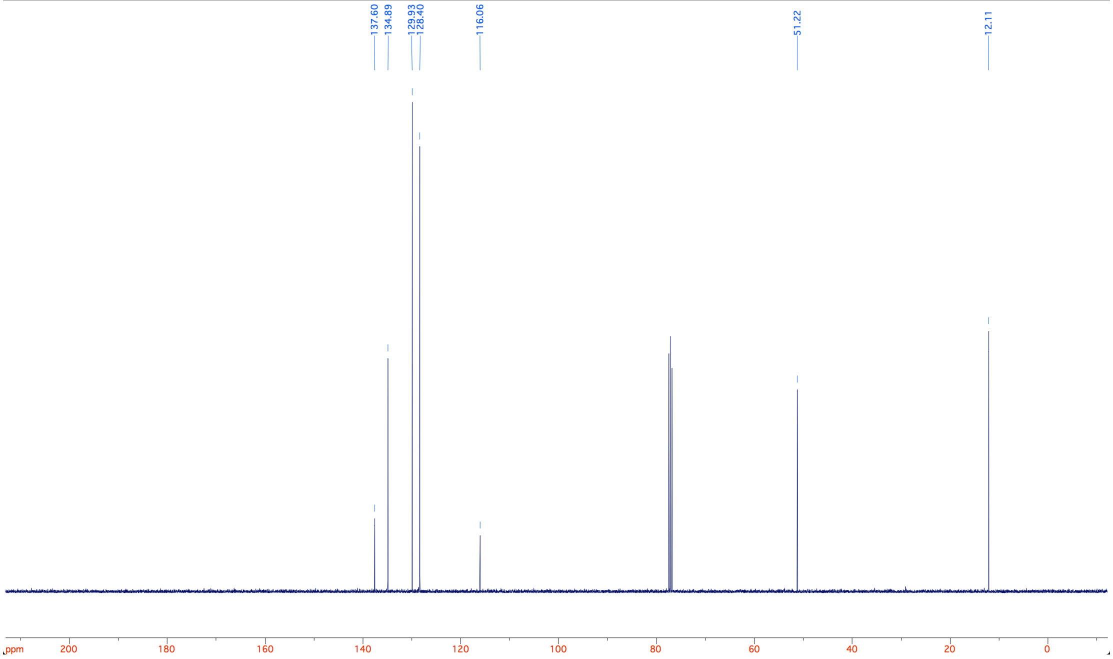

## (±)-Methyl 2-benzyl-3-cyanopropanoate (15)

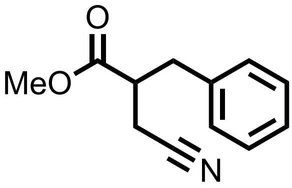


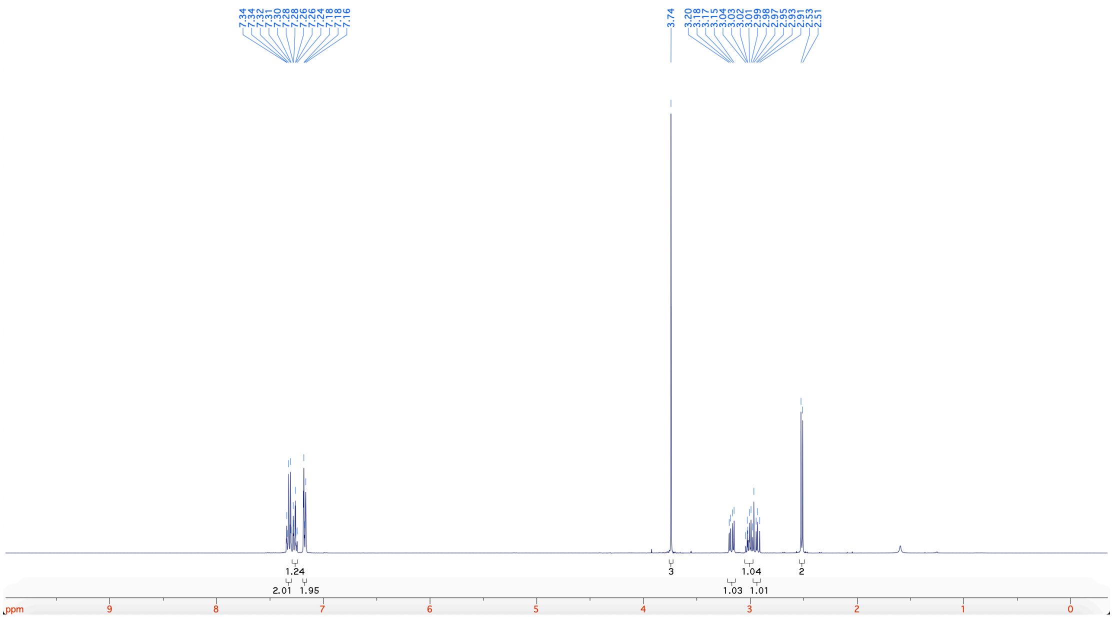


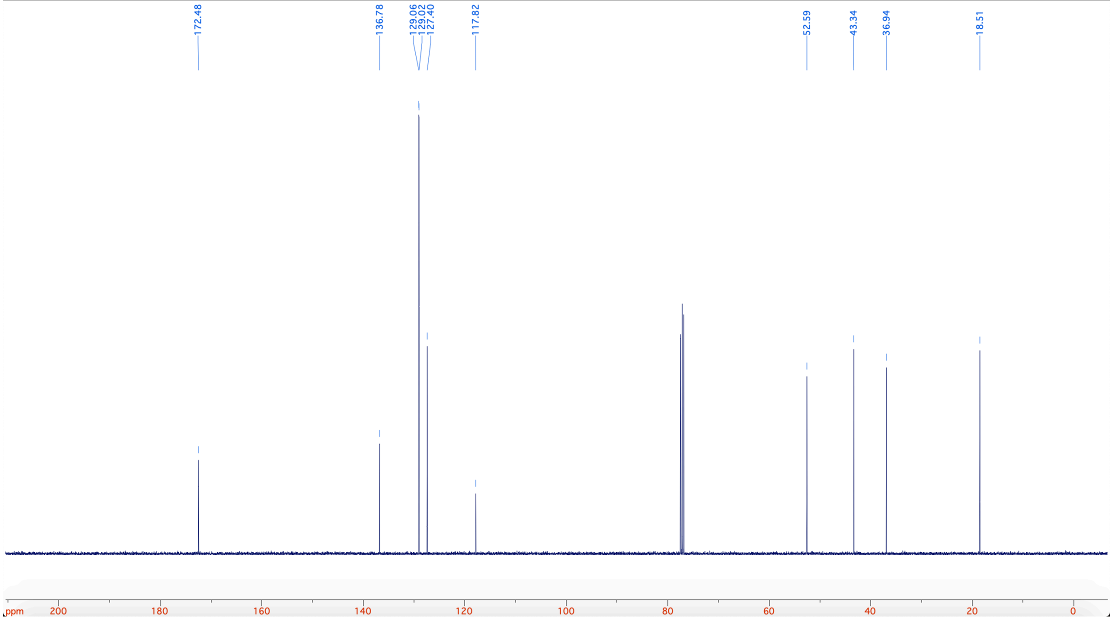

## (±)-3-Methyl-4-morpholino-4-oxobutanenitrile (16)

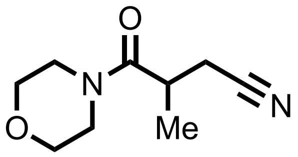


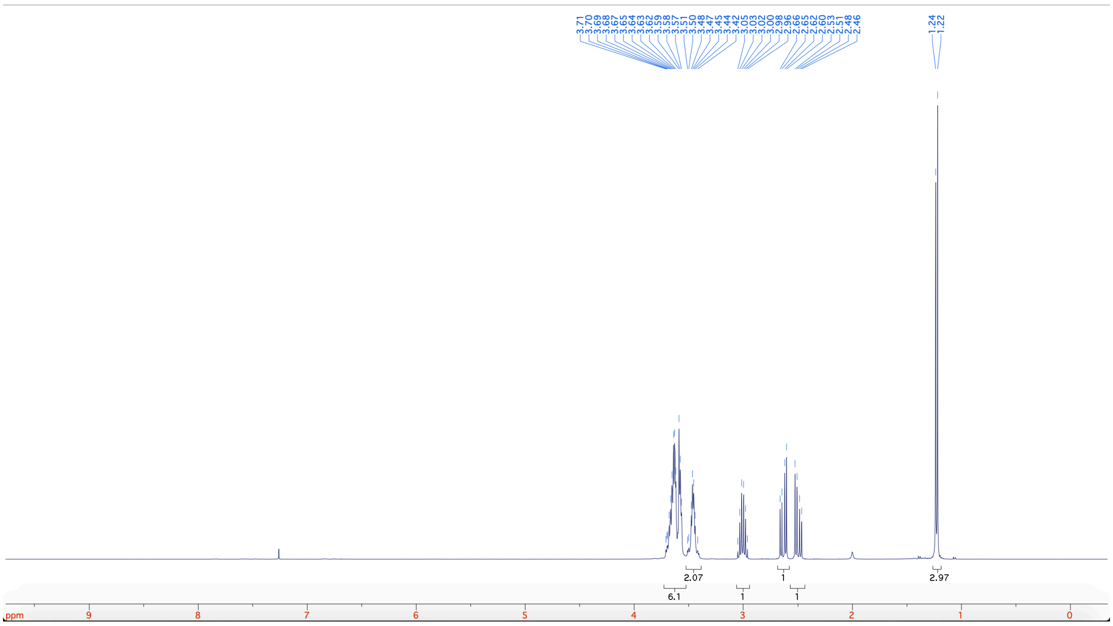


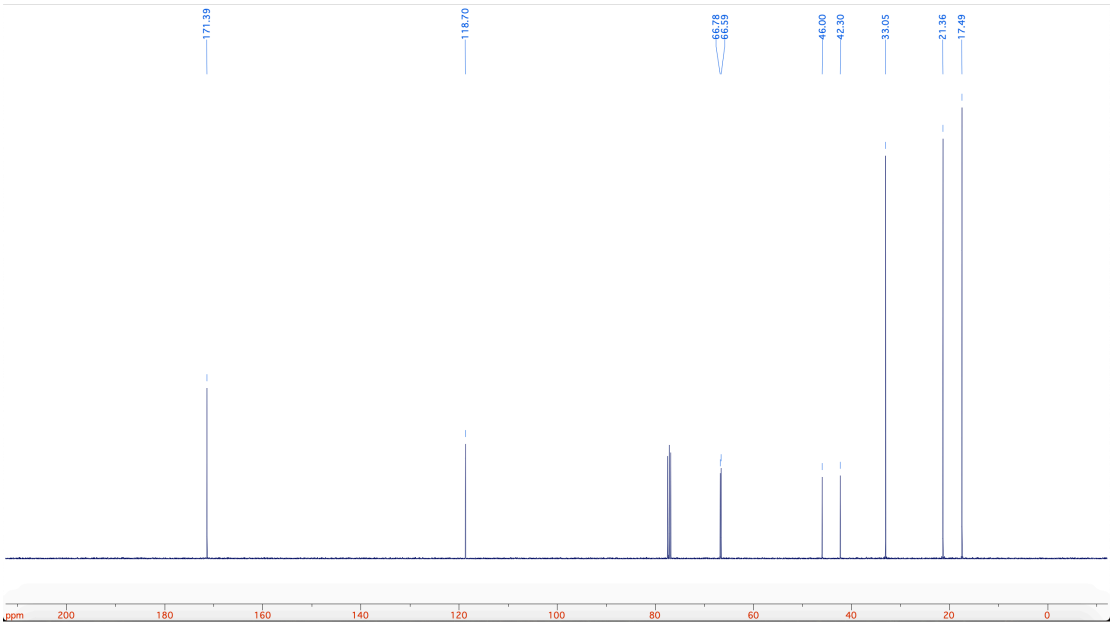

**3-(6-Bromopyridin-2-yl)propanenitrile (17)**

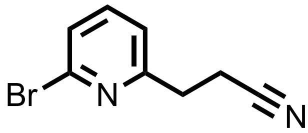


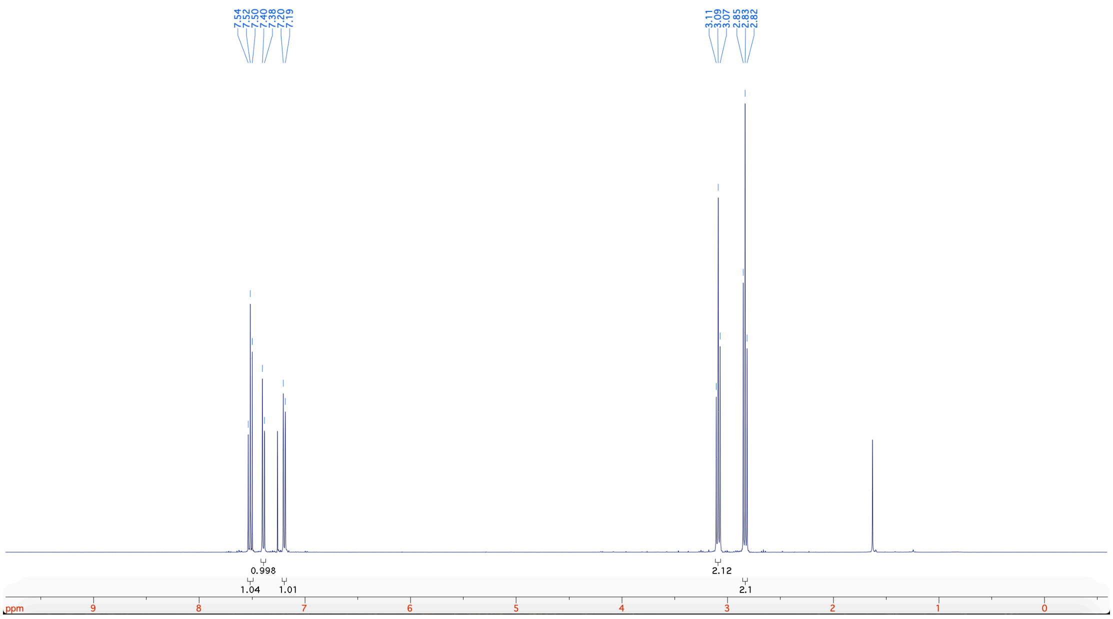


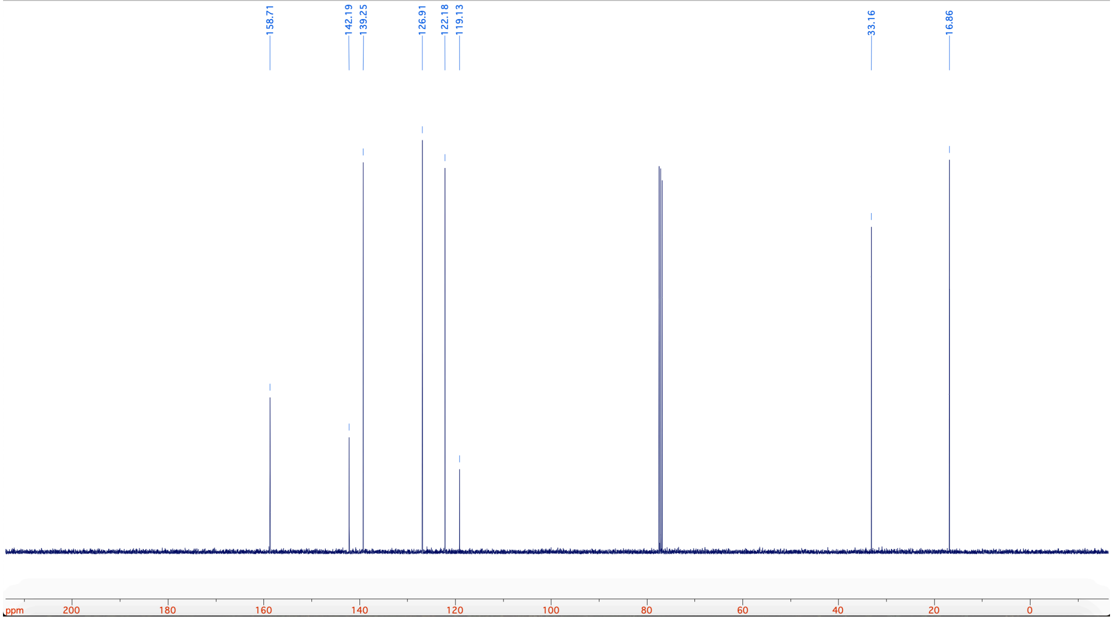

**3-(Pyrazin-2-yl)propanenitrile (18)**

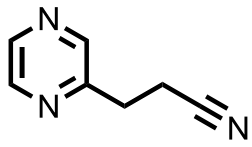

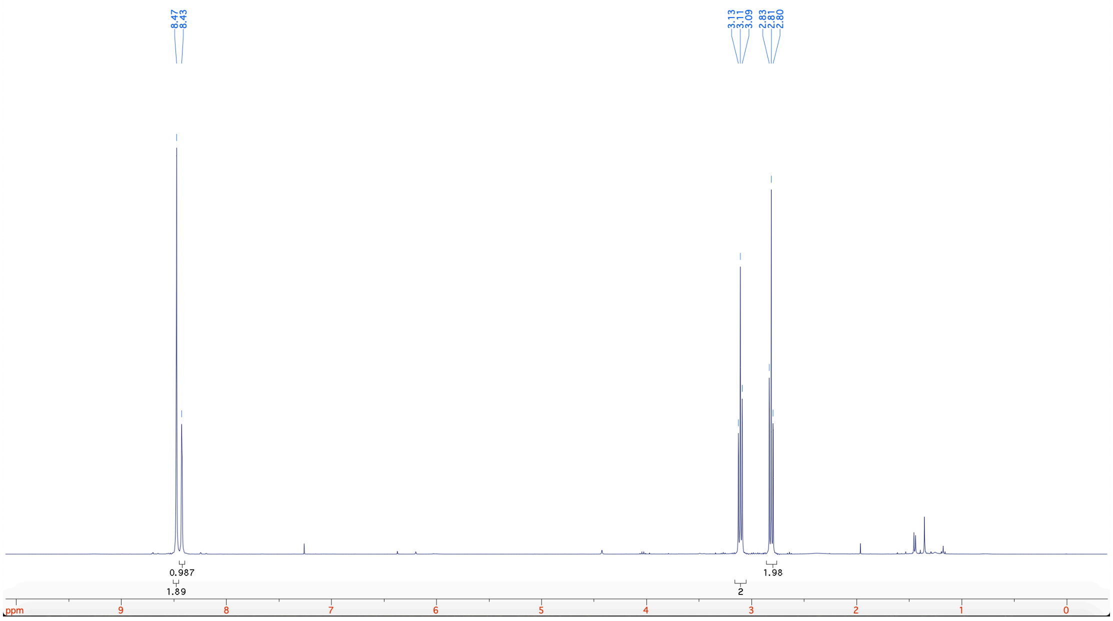


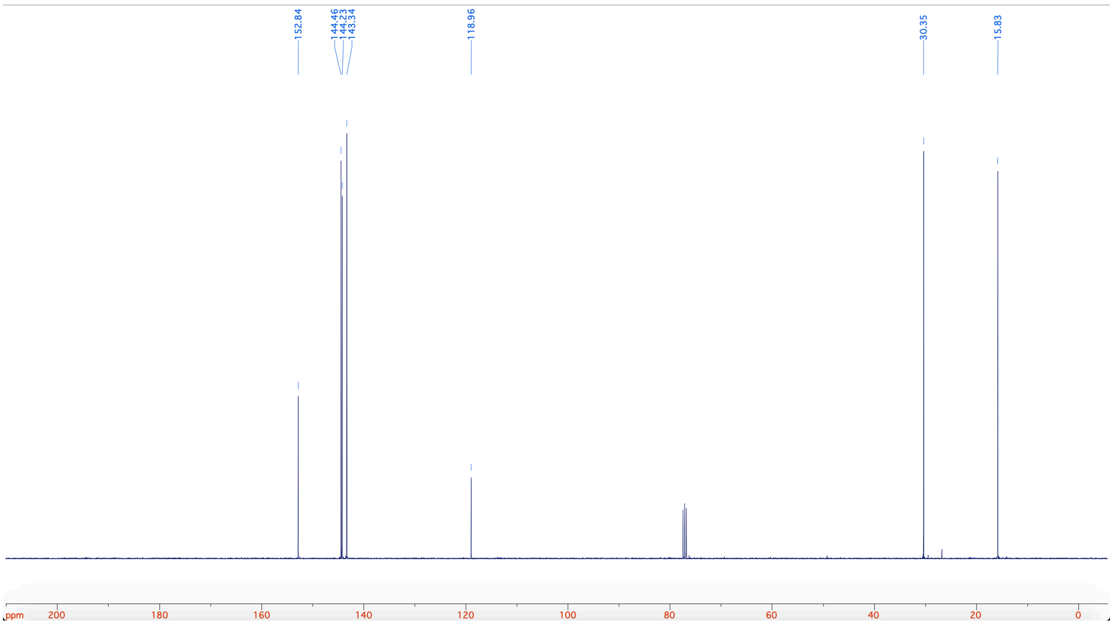

**Tridecanenitrile (19)**

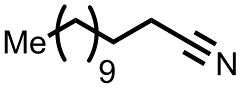


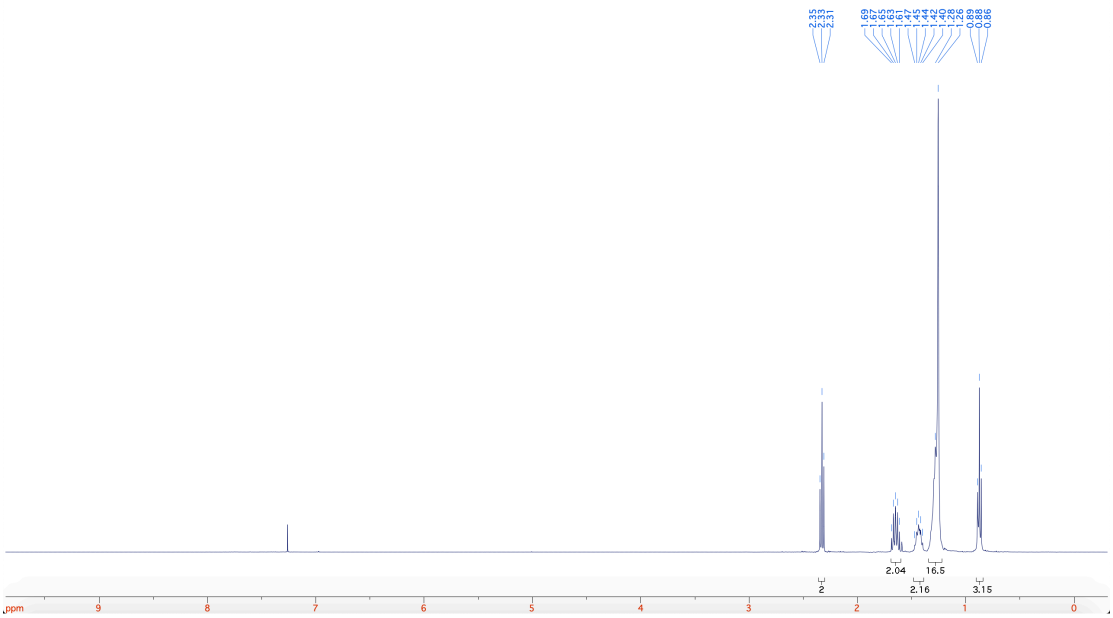


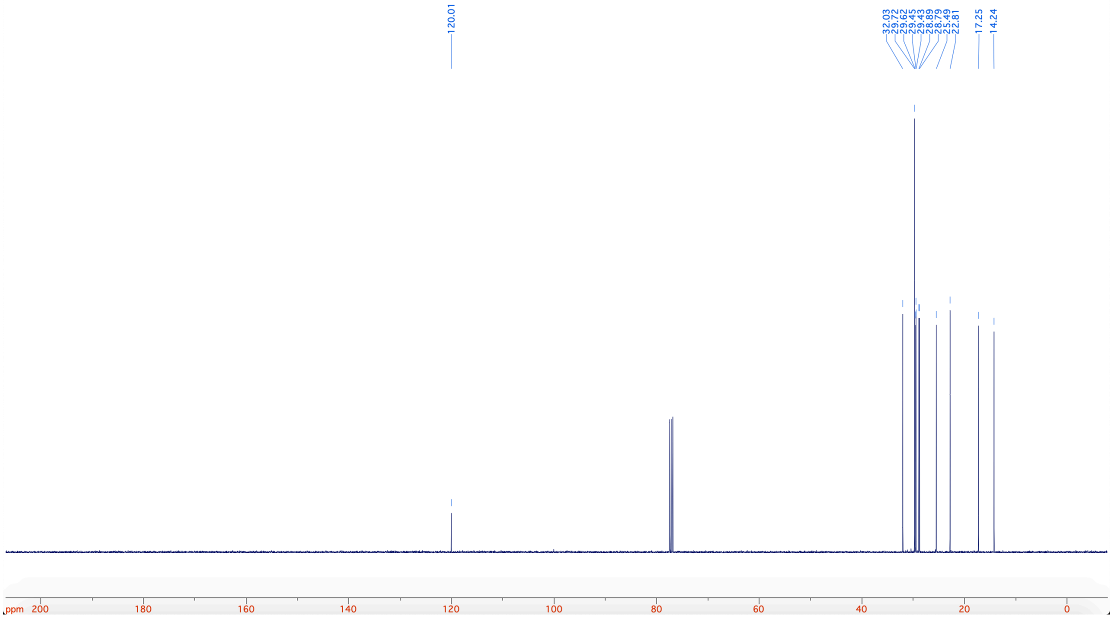

# 4-Phenylbutanenitrile (20)

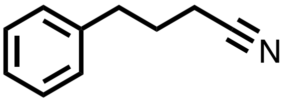


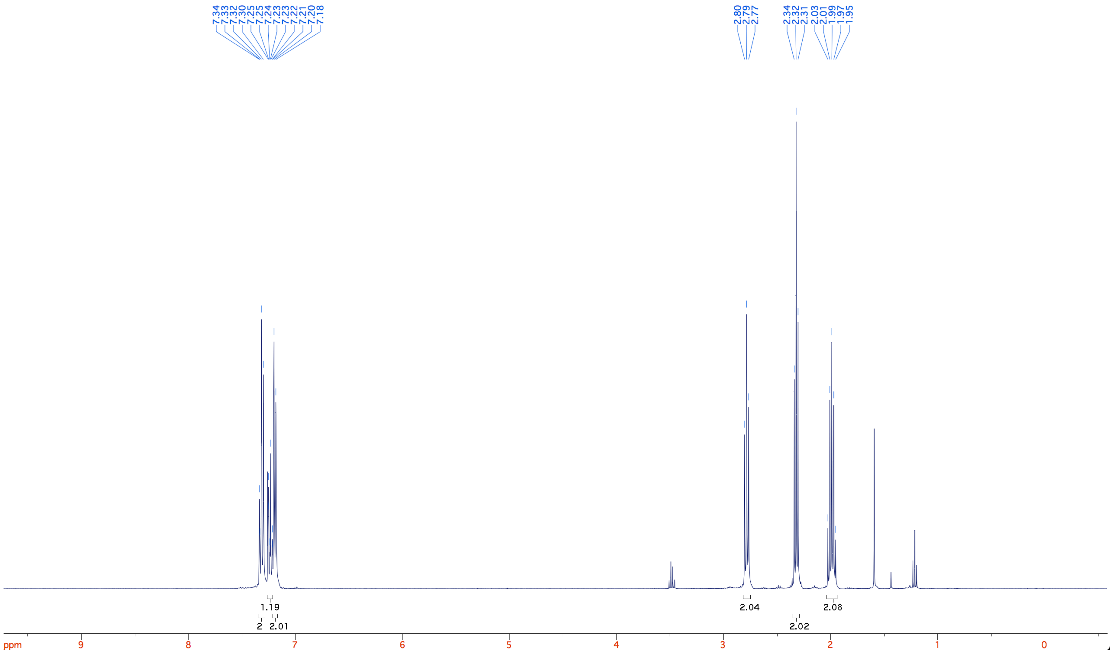


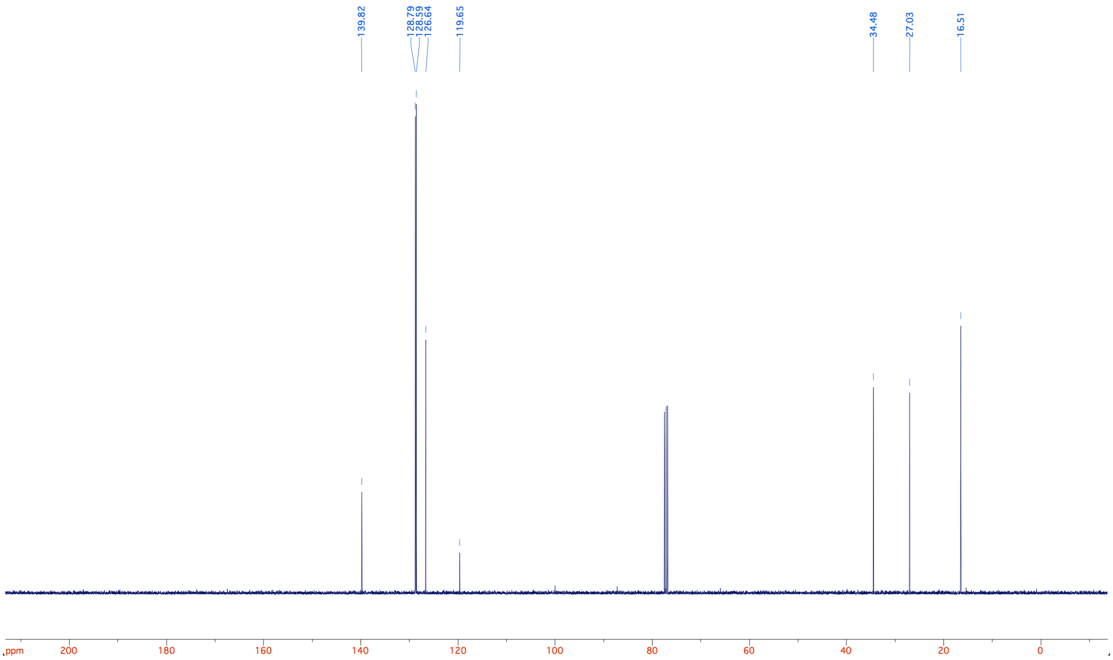

## 6-Oxo-6-phenylhexanenitrile (21)

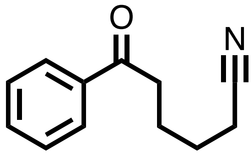


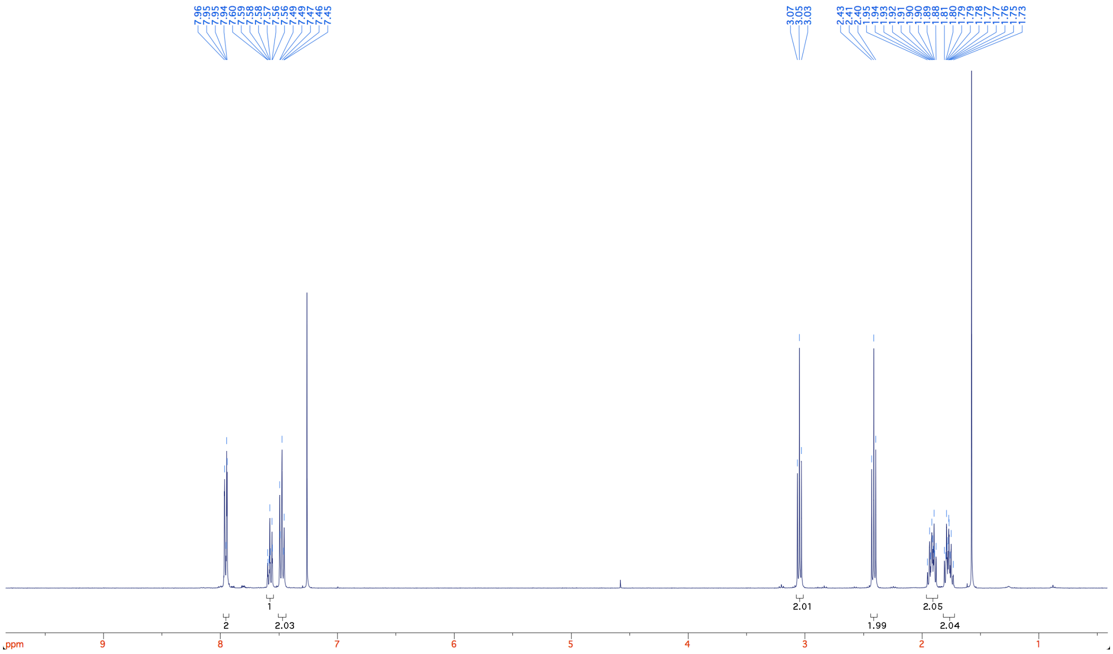


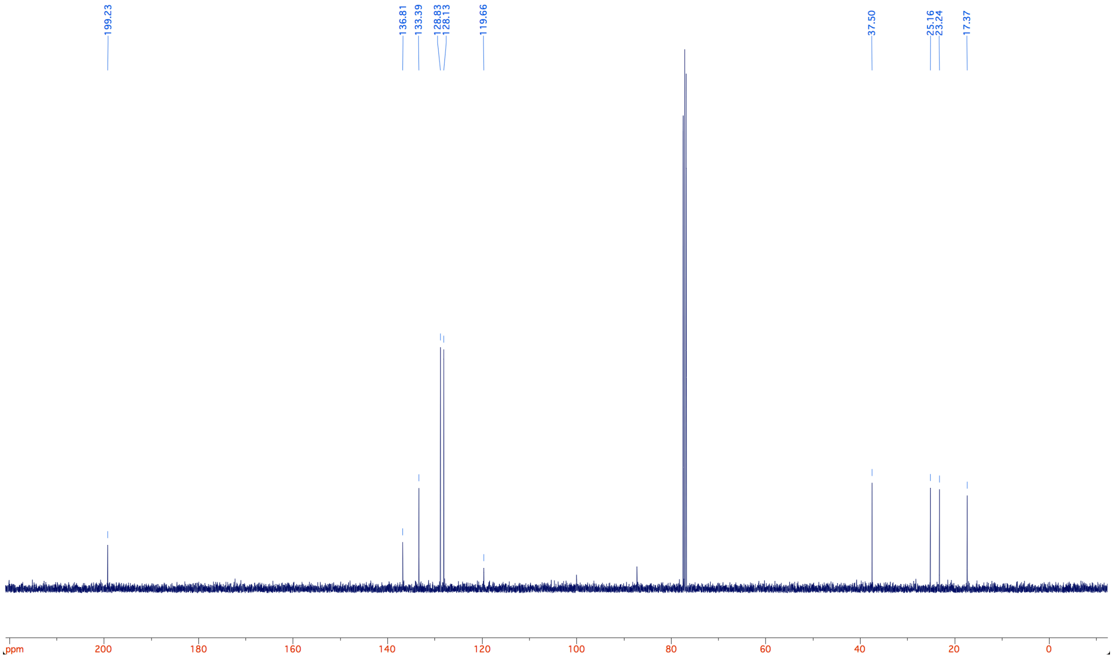

## (*S*)-*tert*-Butyl 2-((*tert*-butoxycarbonyl)amino)-5-cyanopentanoate (22)

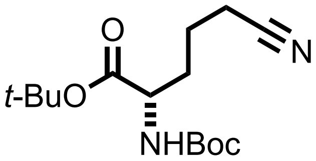


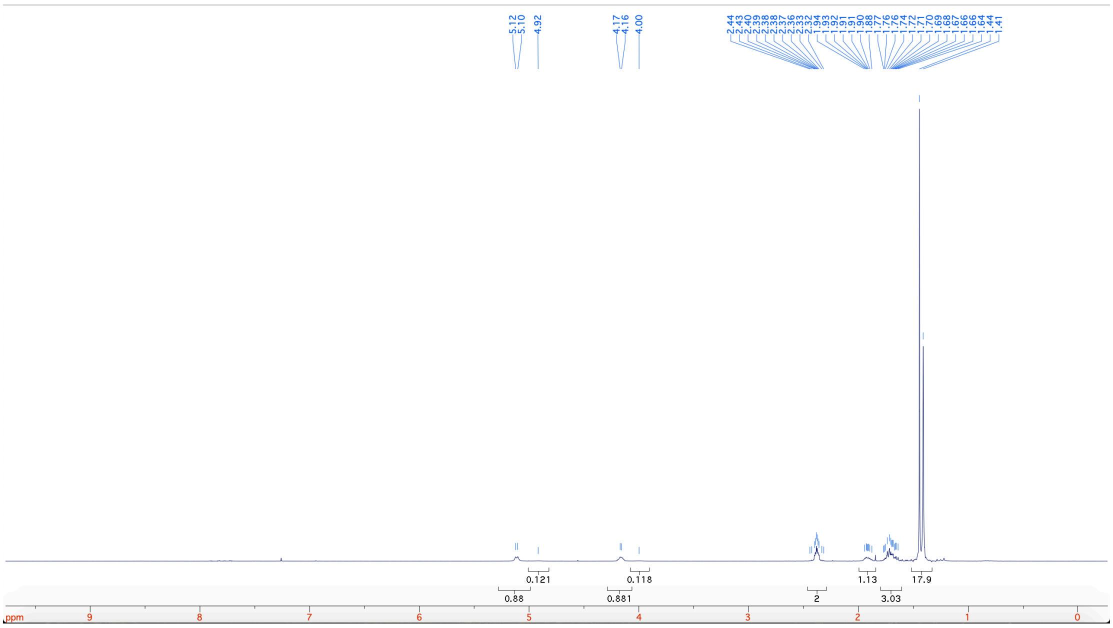


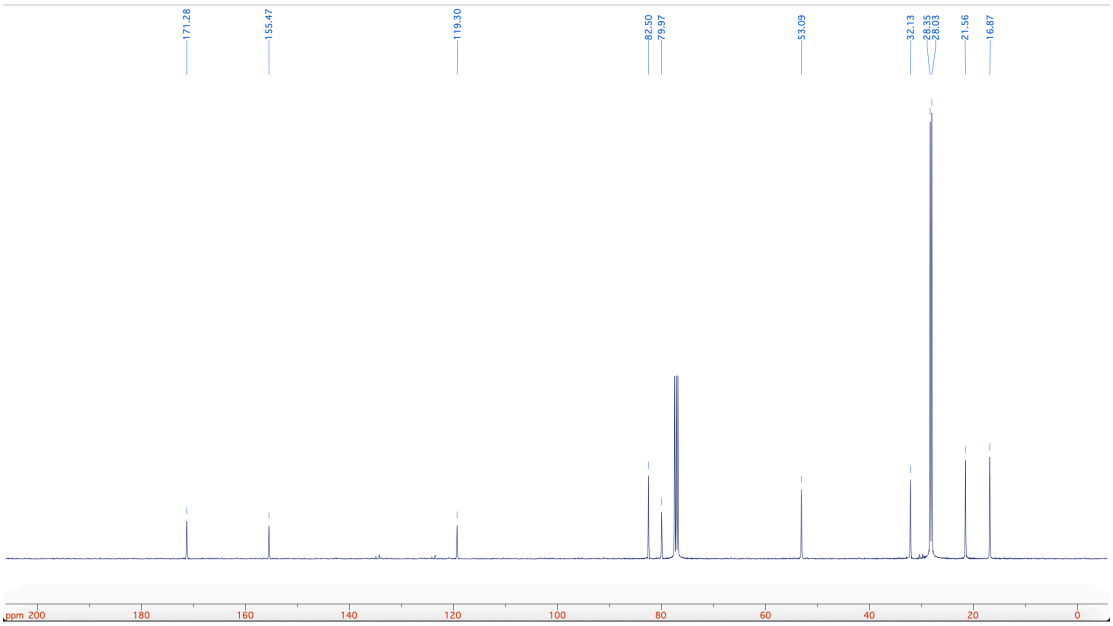

## ***tert*-Butyl 3-(2-cyanoethyl)azetidine-1-carboxylate (23)**

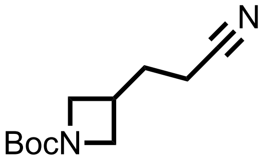


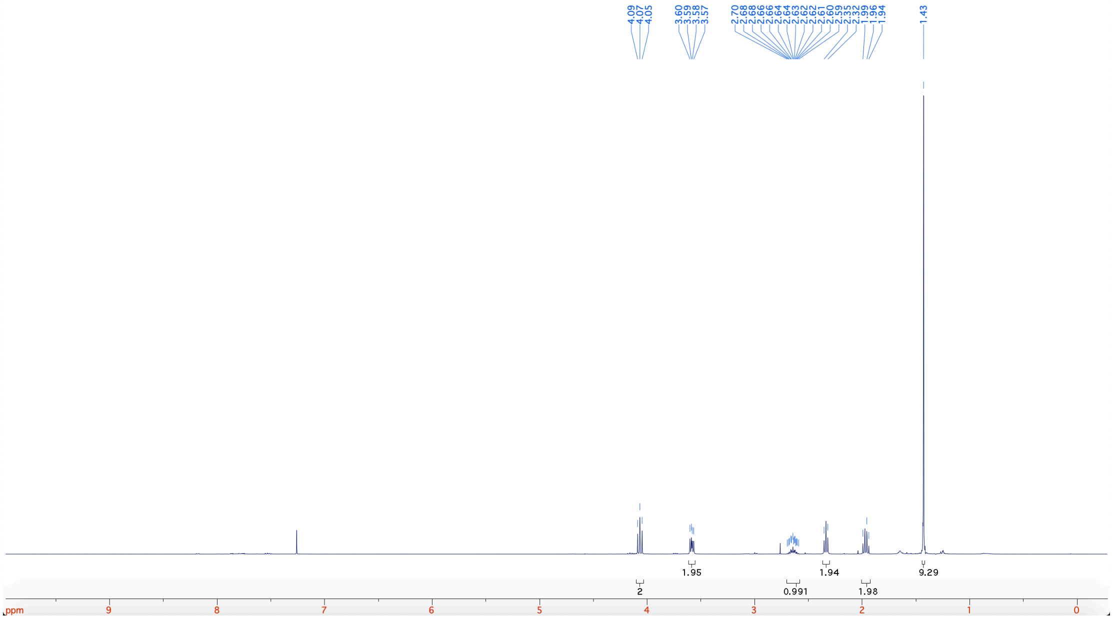


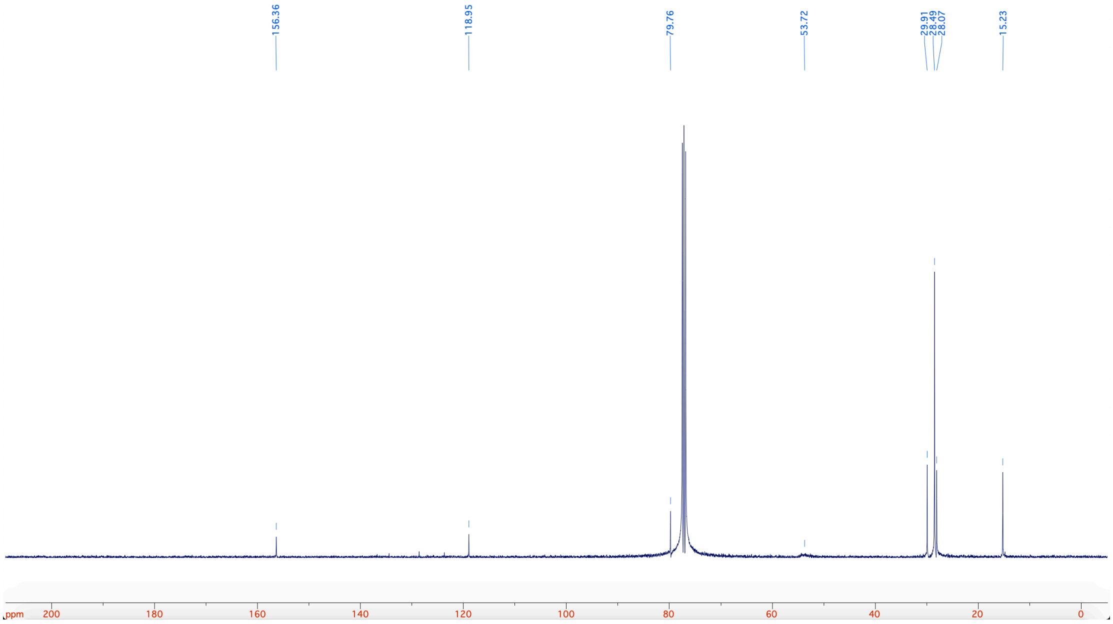

## 3-(1,3-Dioxoisoindolin-2-yl)propanenitrile (24)

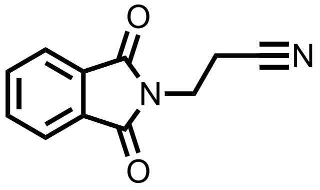


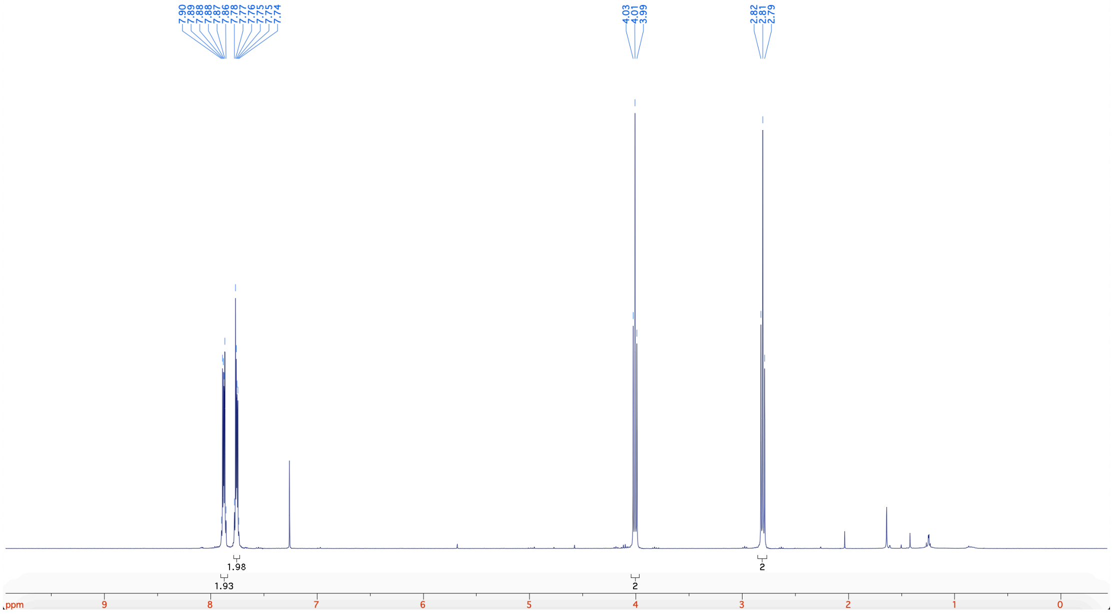


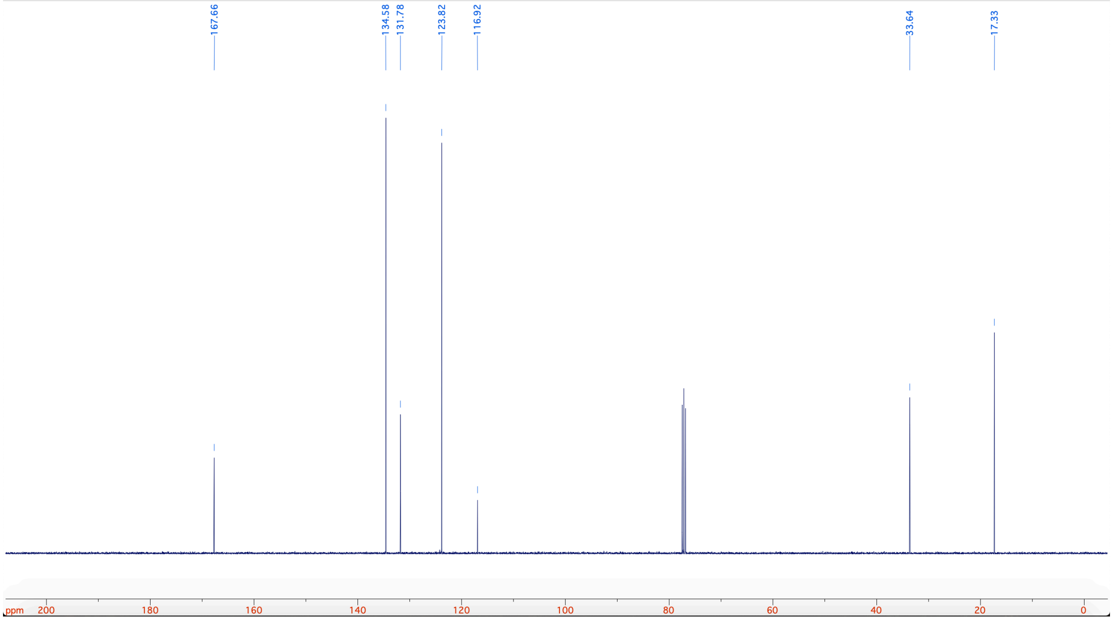

**Diethyl (2-cyanoethyl)phosphonate (25)**

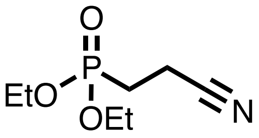


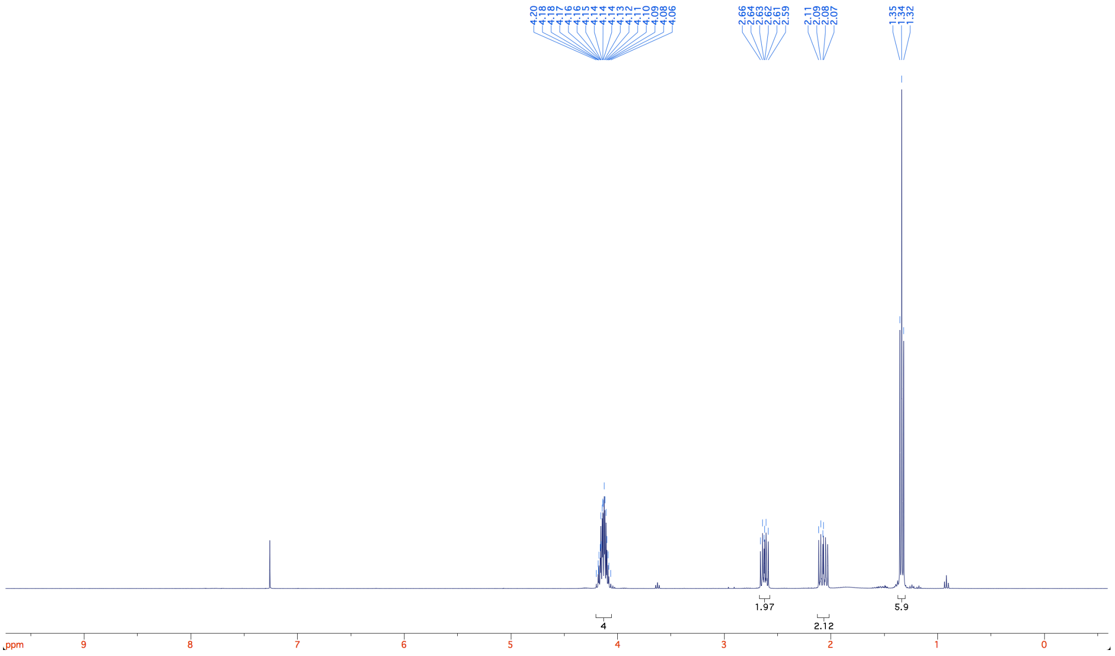


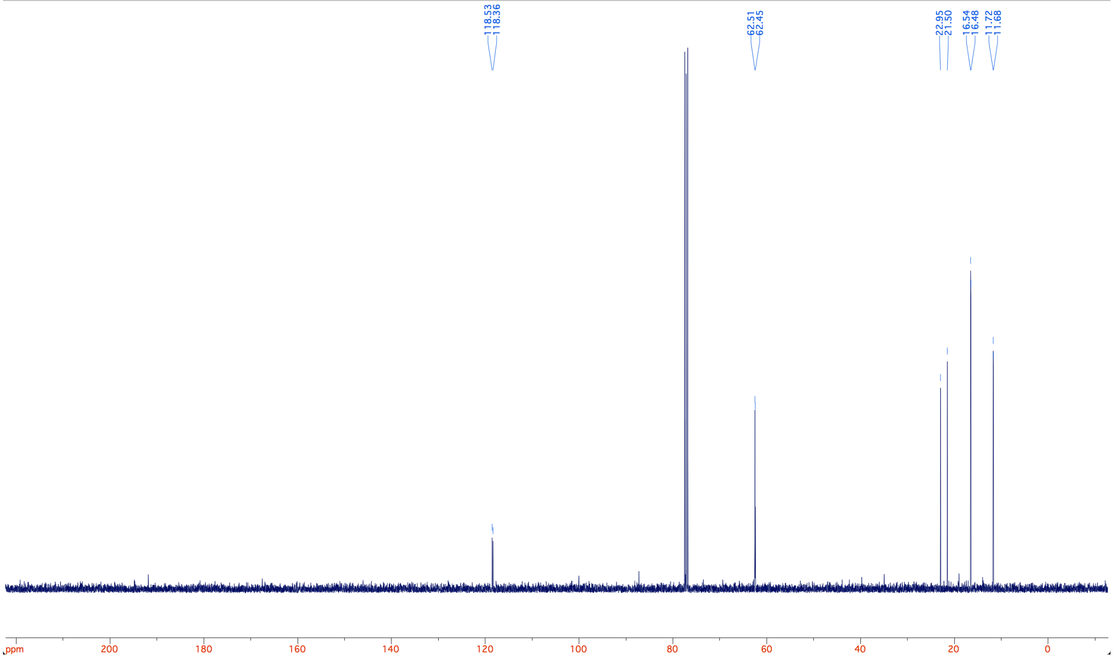

**tert-Butyl 4-(cyanomethyl)piperidine-1-carboxylate (26)**

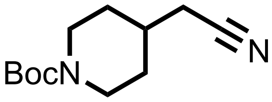

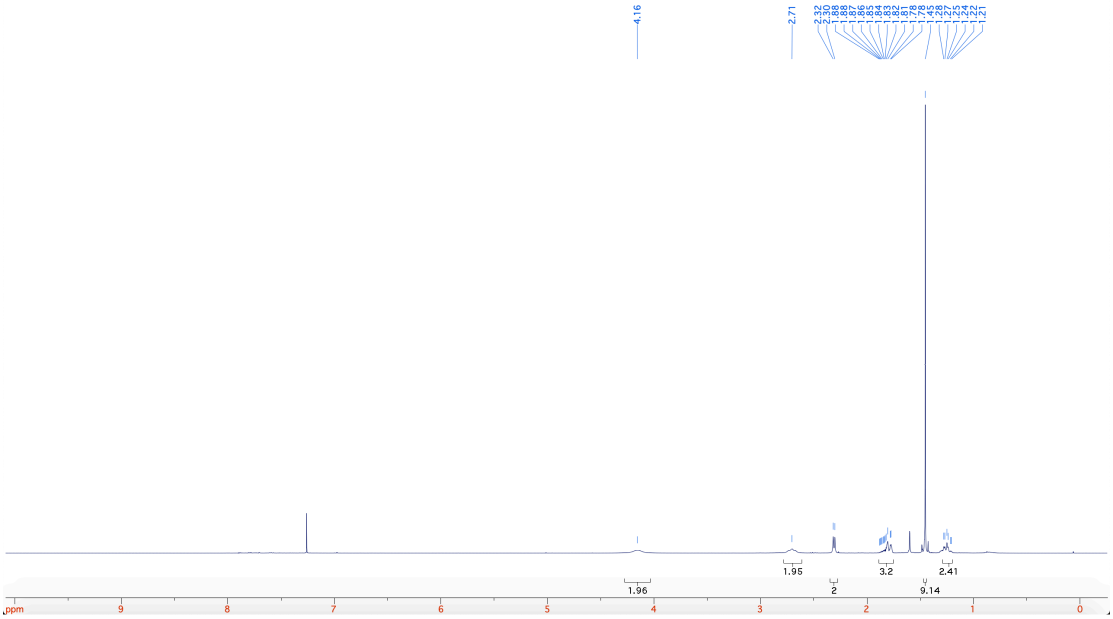


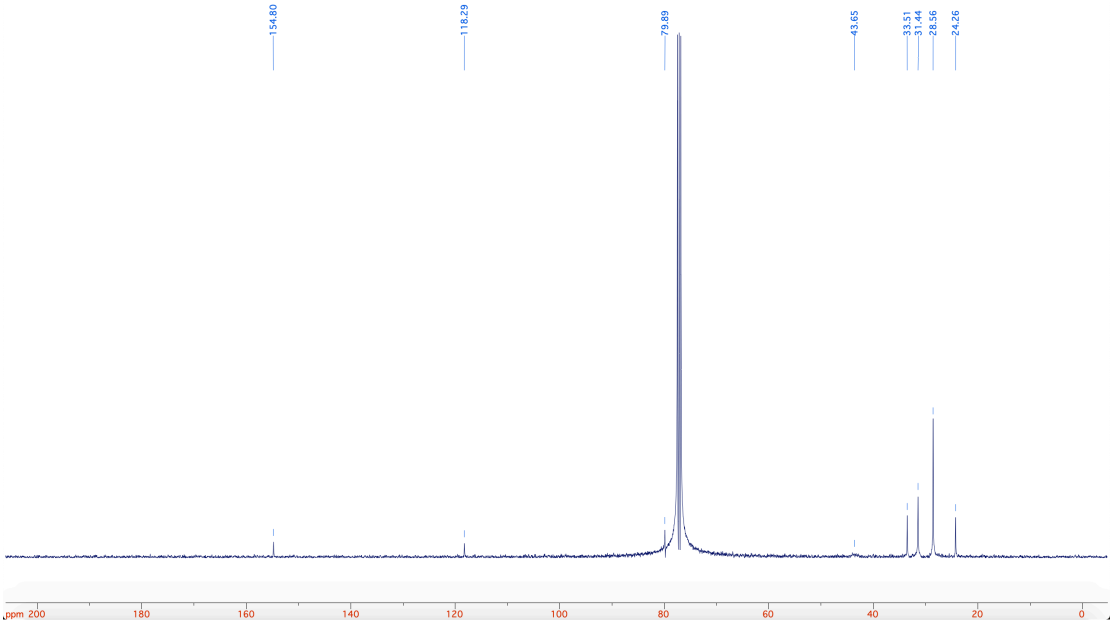

**2-(tetrahydro-2*H*-pyran-4-yl)acetonitrile (27)**

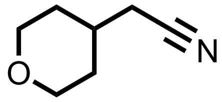


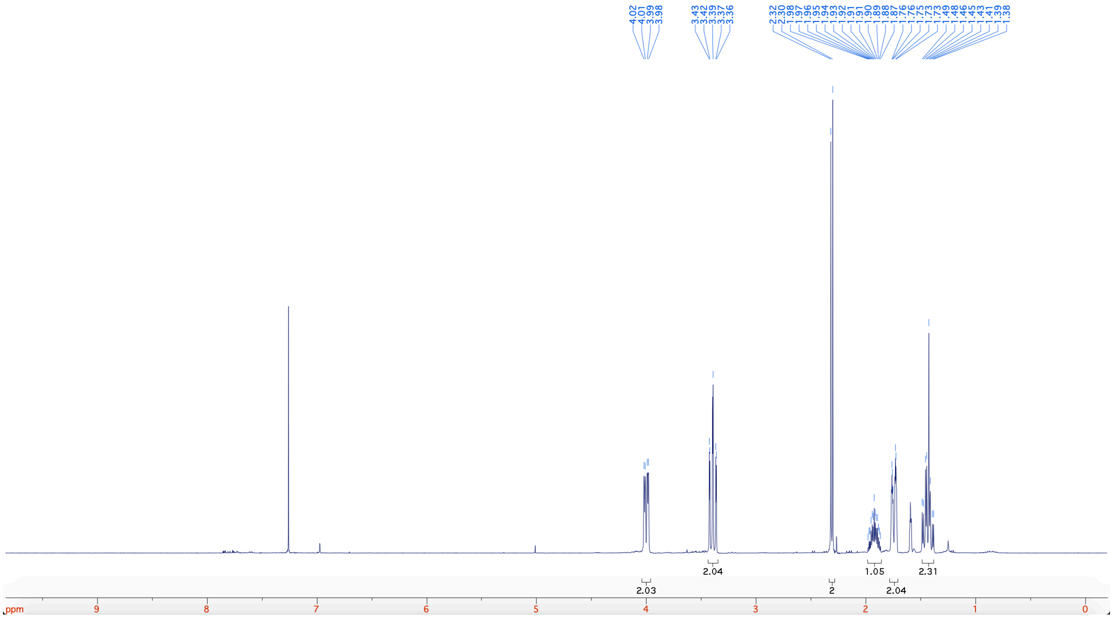


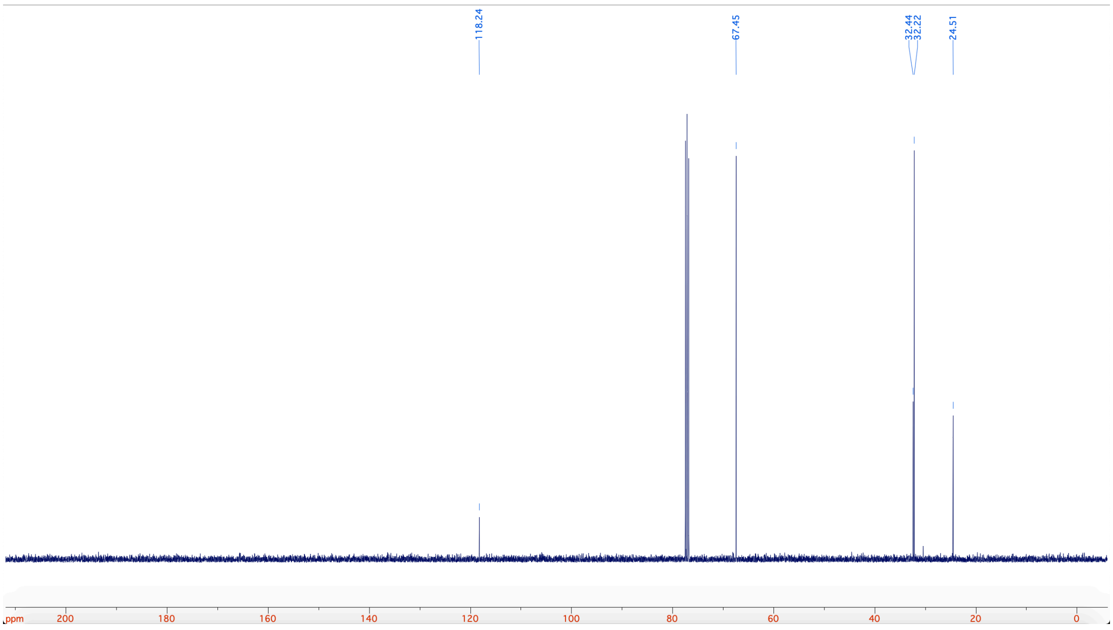

## 2-(2,3-Dihydro-1*H*-inden-2-yl)acetonitrile (28)

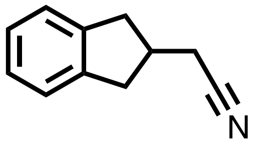


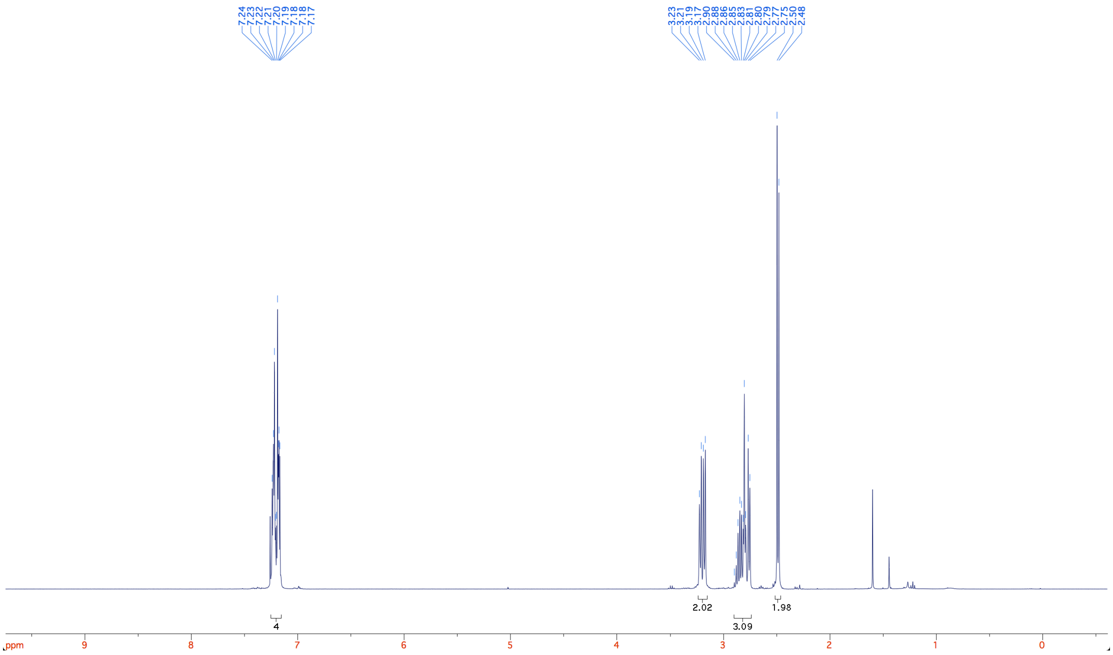


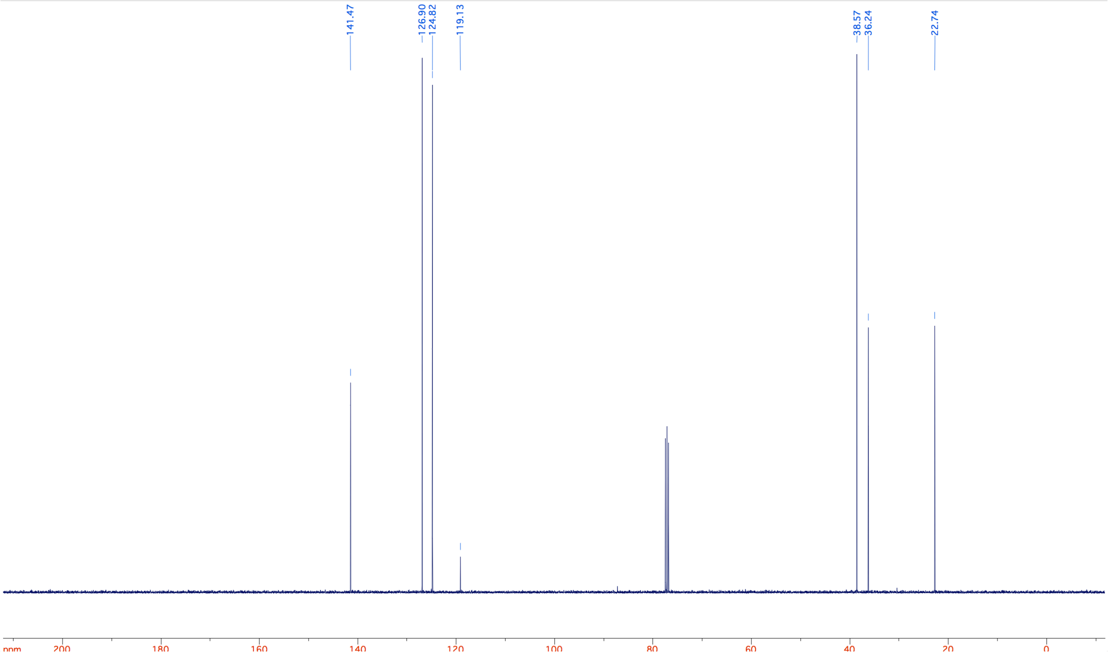

## (±)-*tert*-Butyl 2-(cyanomethyl)pyrrolidine-1-carboxylate (29)

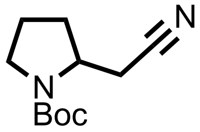


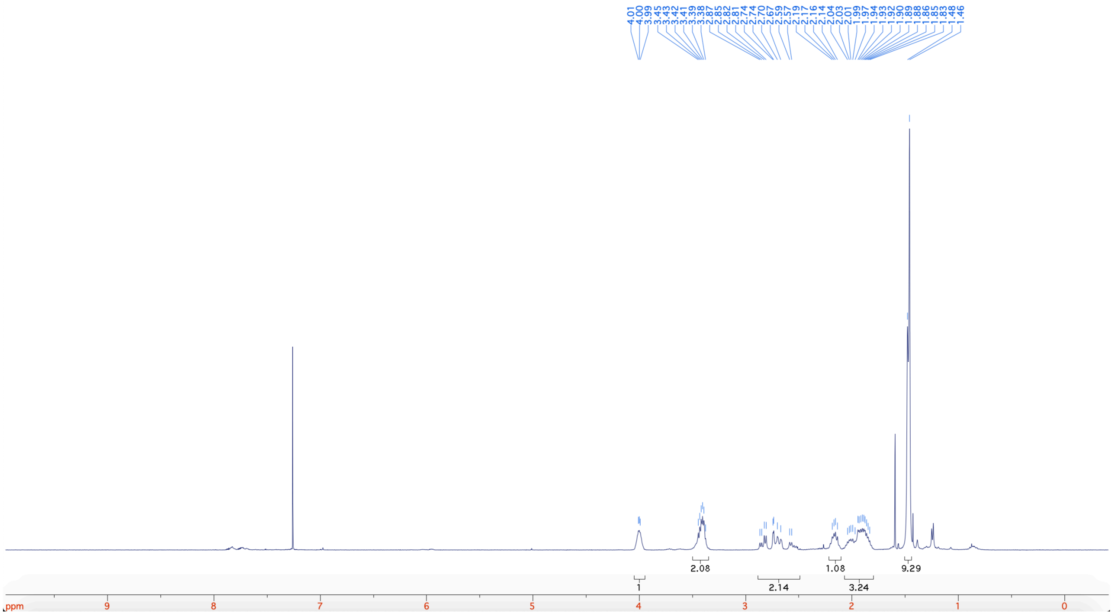


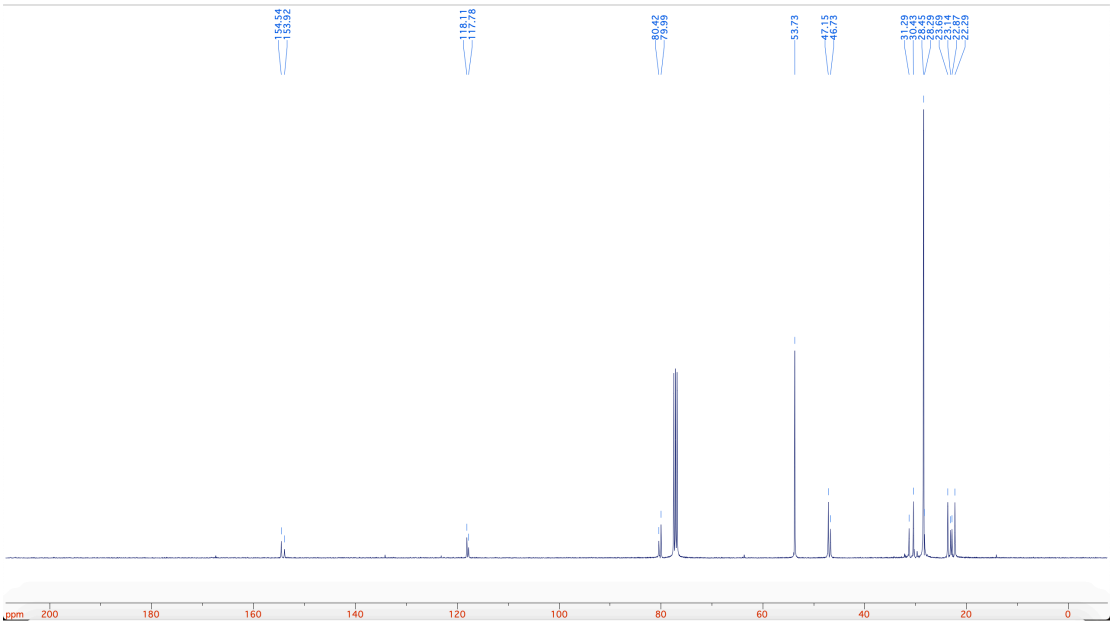

## 2-(Cyclohexylsulfonyl)acetonitrile (30)

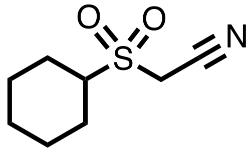


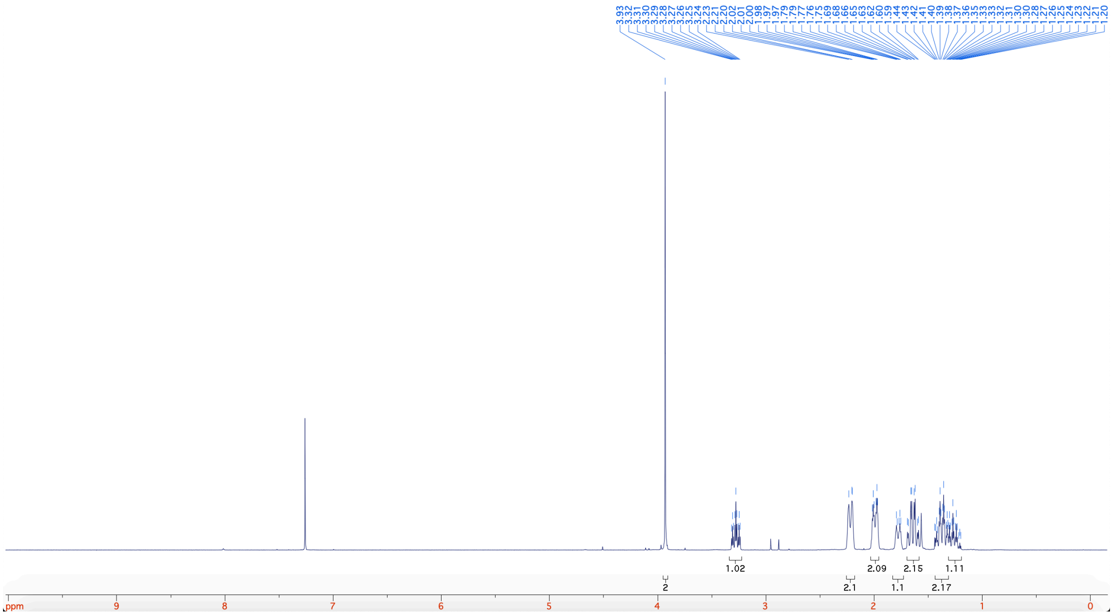


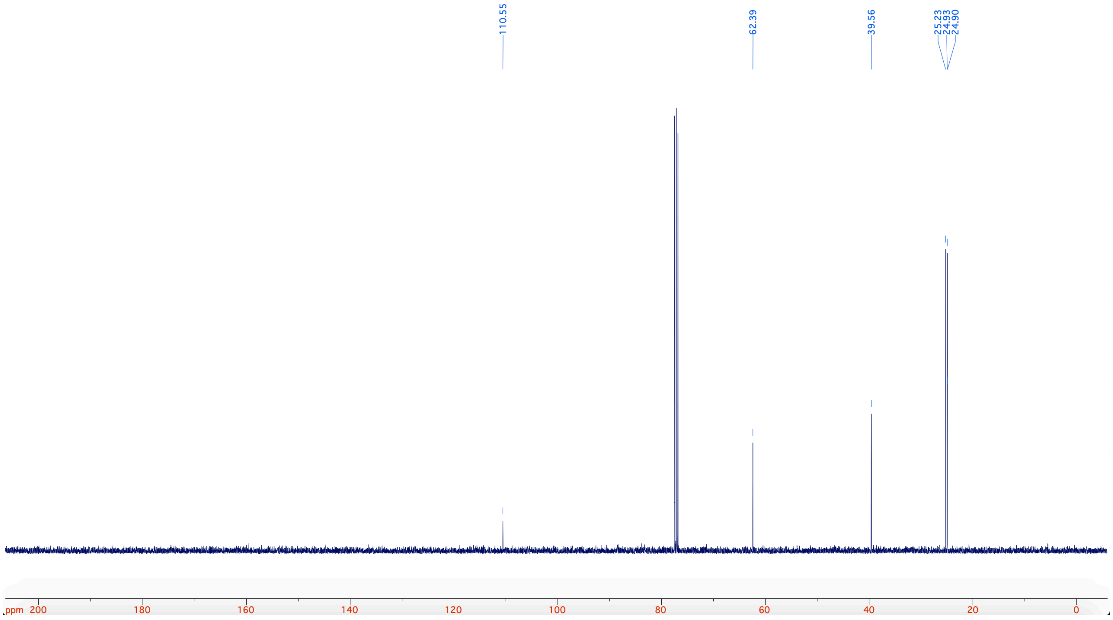

## 2-((((1*R*/*S*,4*S*/*R*)-7,7-dimethyl-2-oxobicyclo[2.2.1]heptan-1-yl)methyl) sulfonyl) acetonitrile (31)

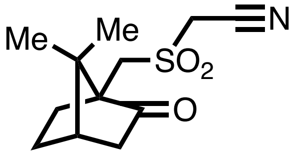


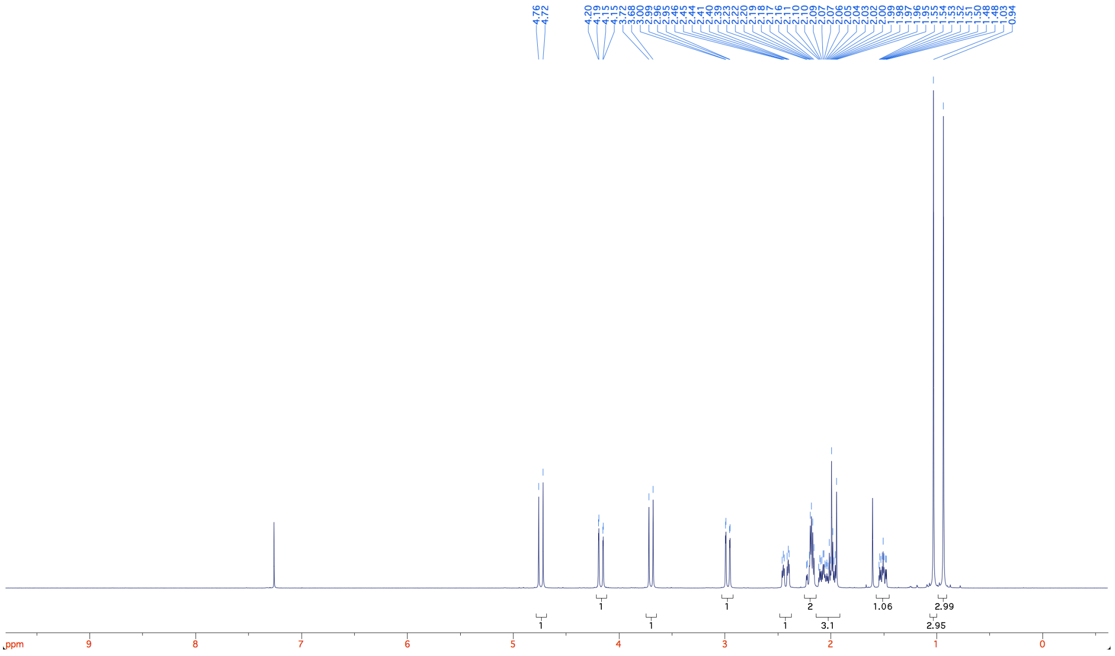


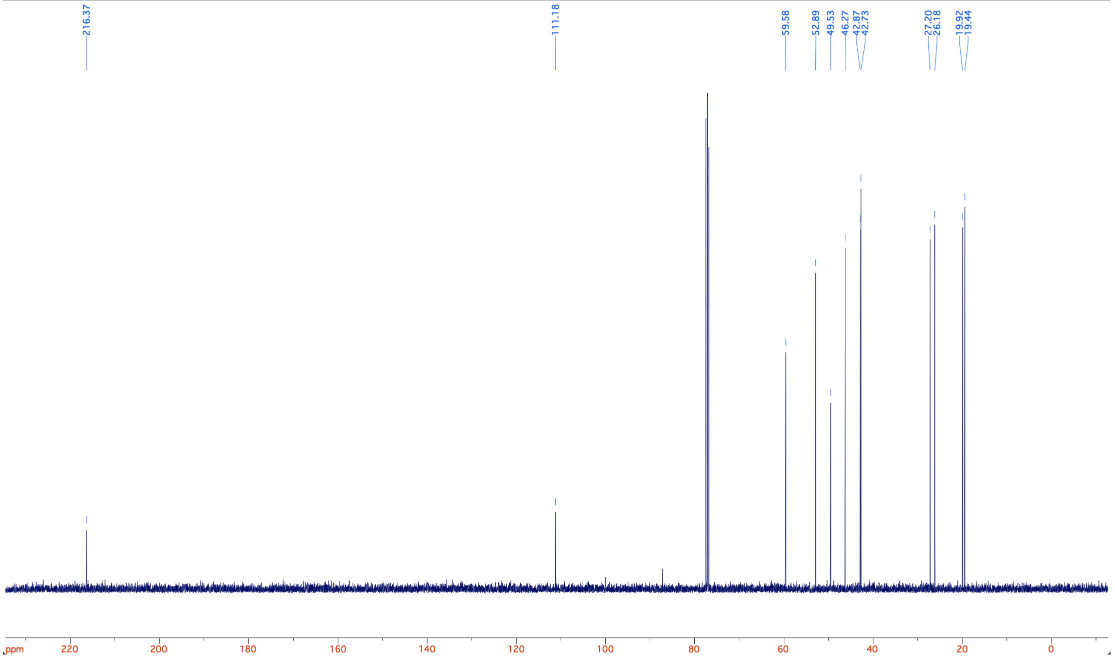

**2-Tosylacetonitrile (32)**

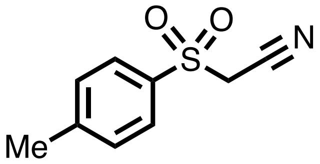


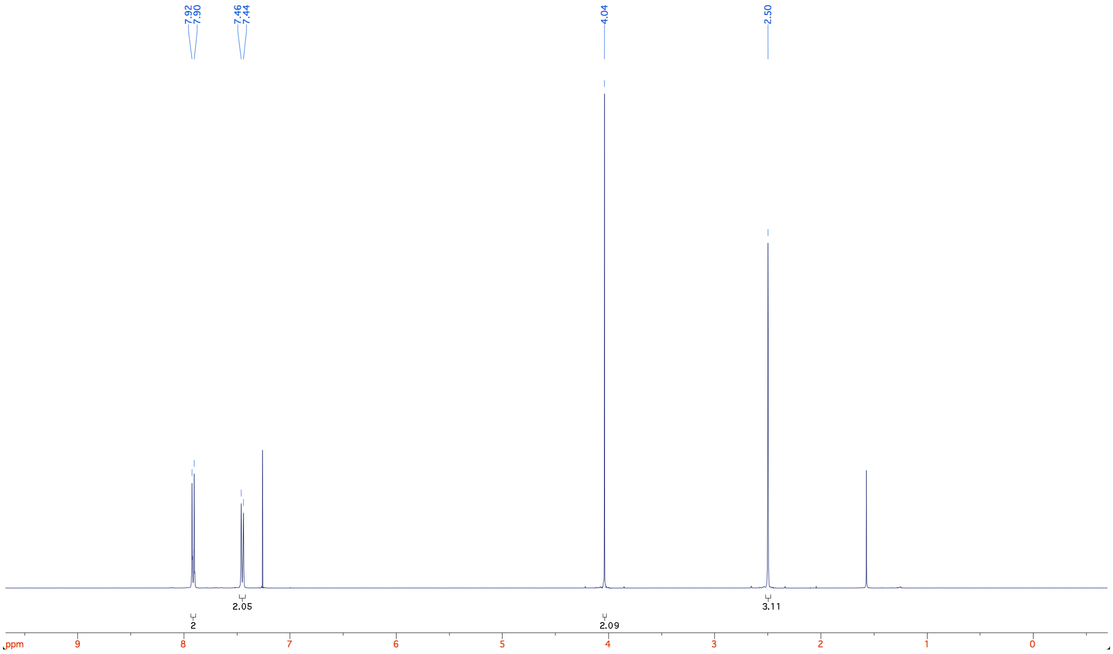


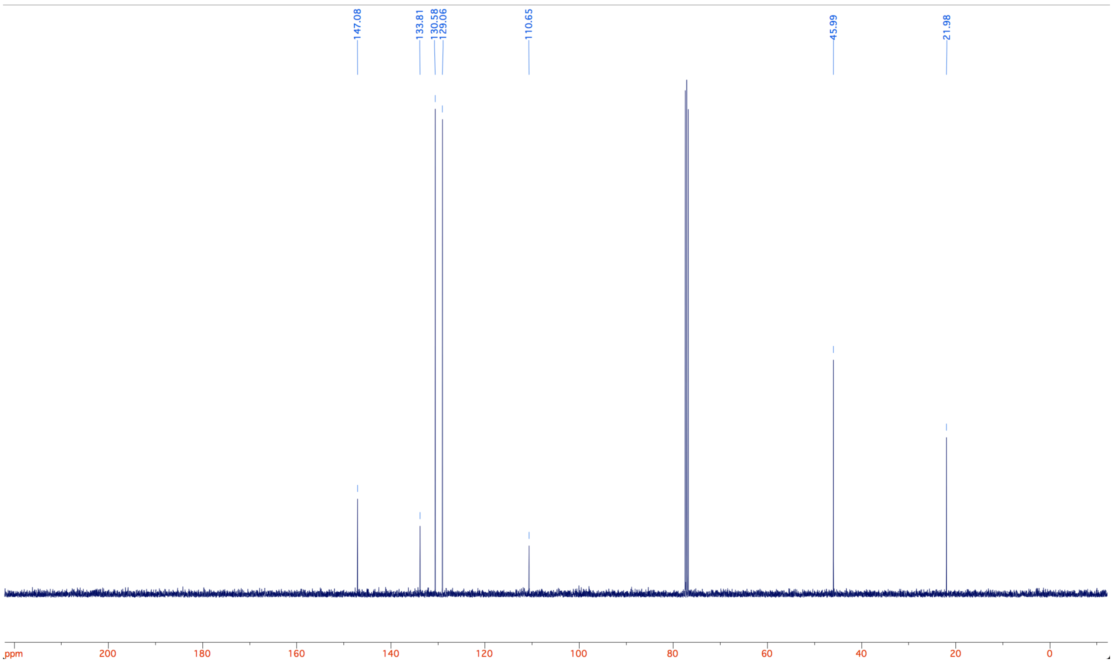

**2-((2,4,6-Triisopropylphenyl)sulfonyl)acetonitrile (33)**

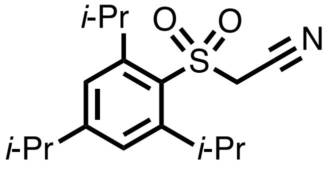


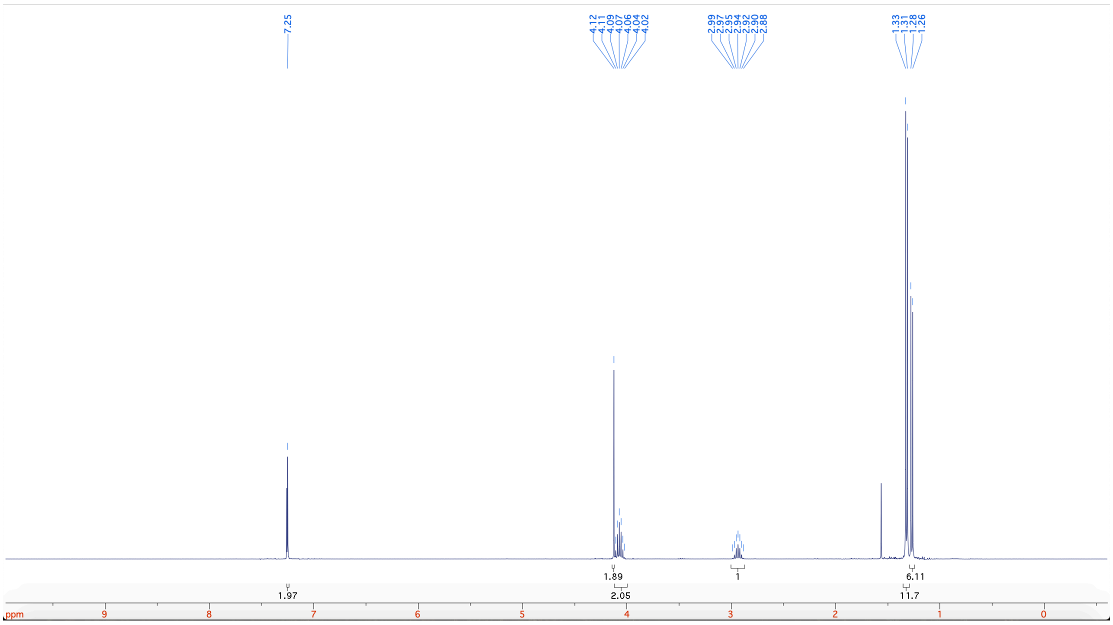


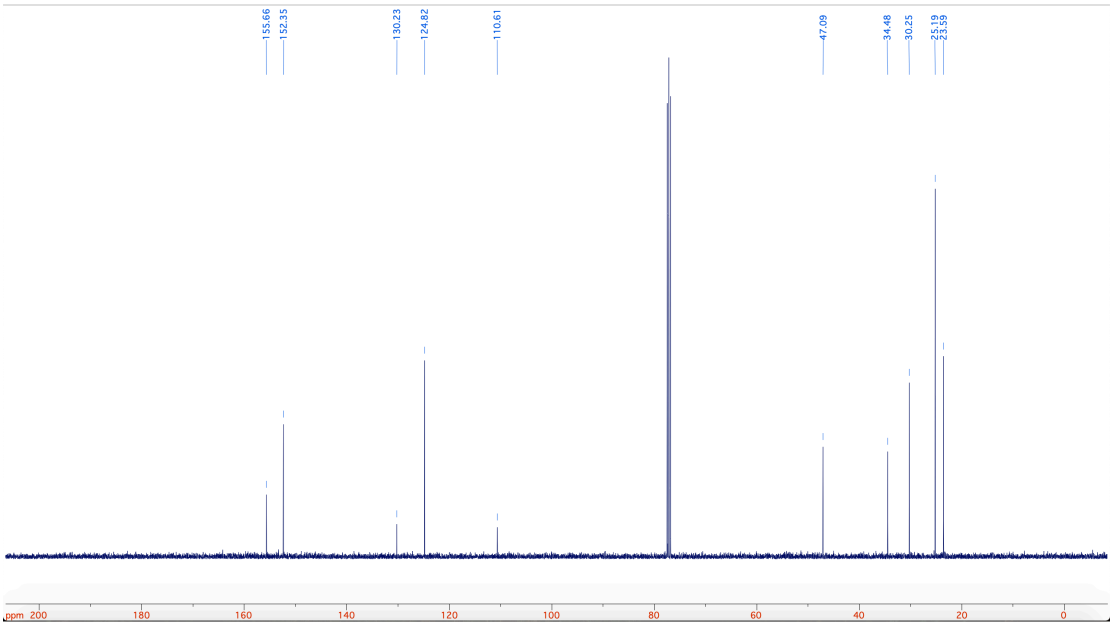

**2-((2-Iodophenyl)sulfonyl)acetonitrile (34)**

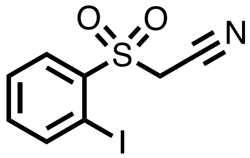


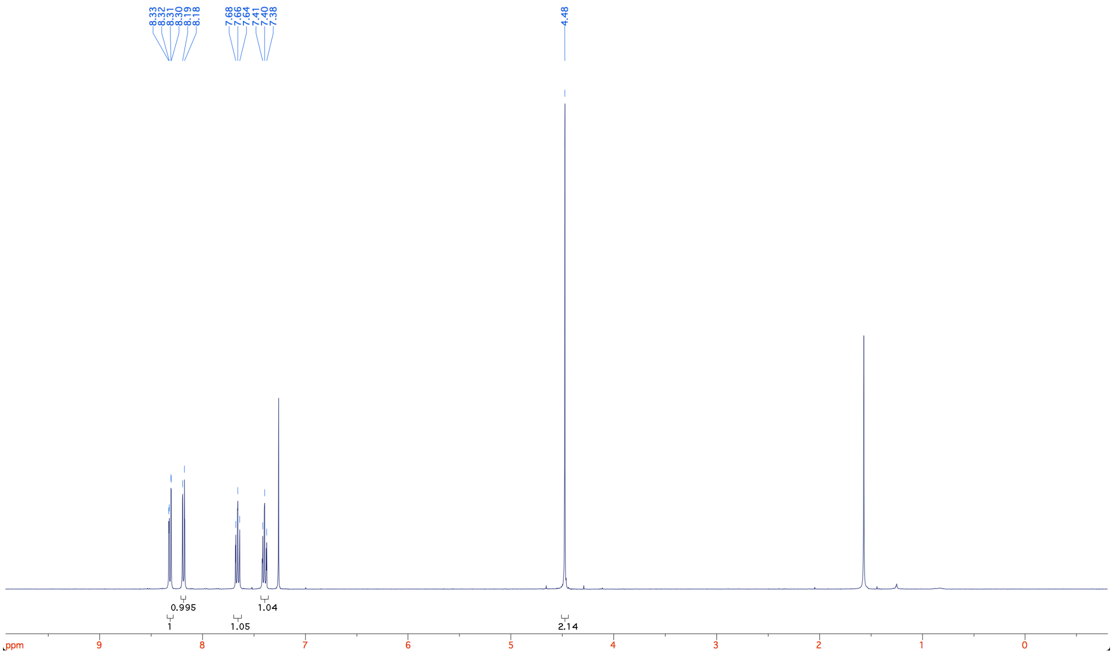


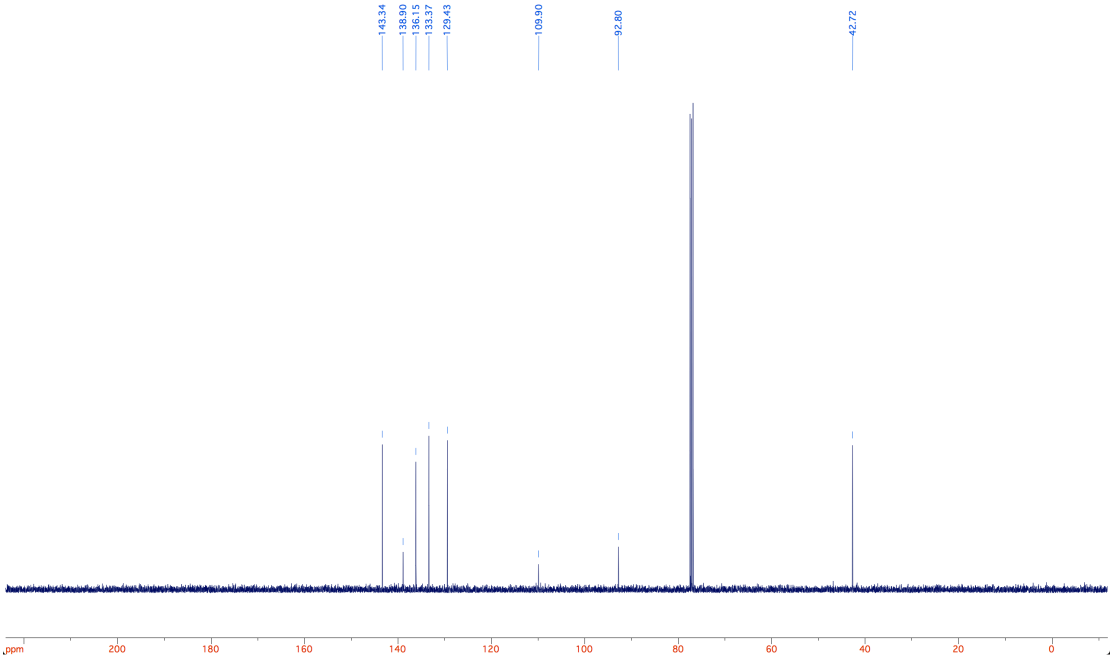

**2-((2-(Cyanomethyl)phenyl)sulfonyl)acetonitrile (35)**

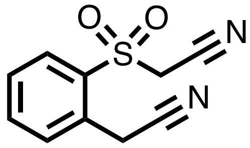


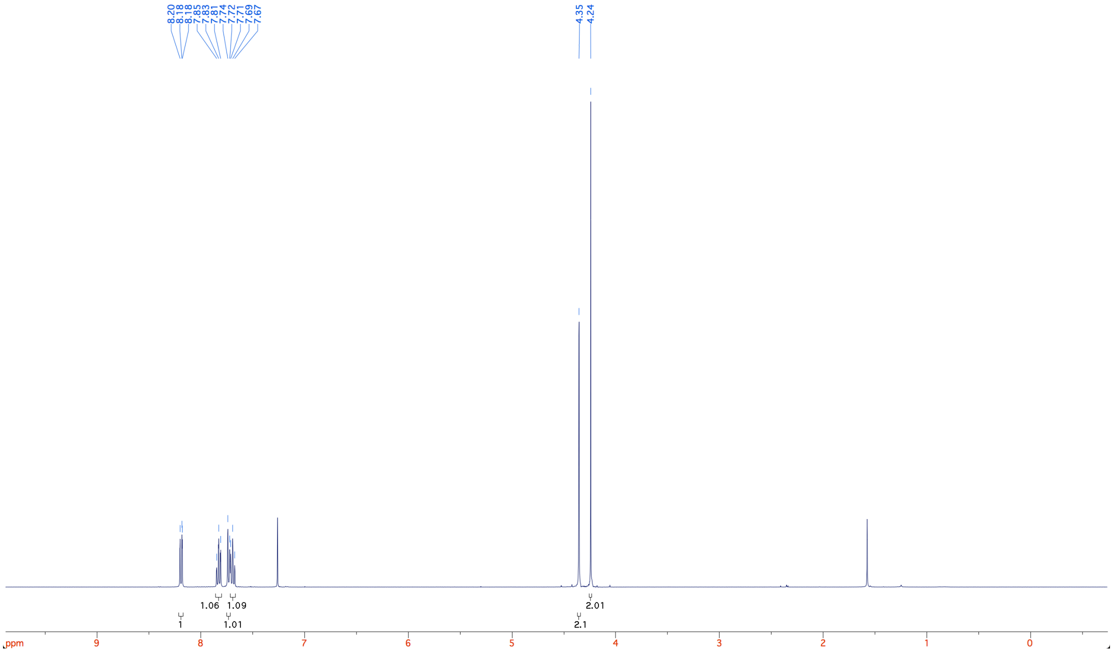


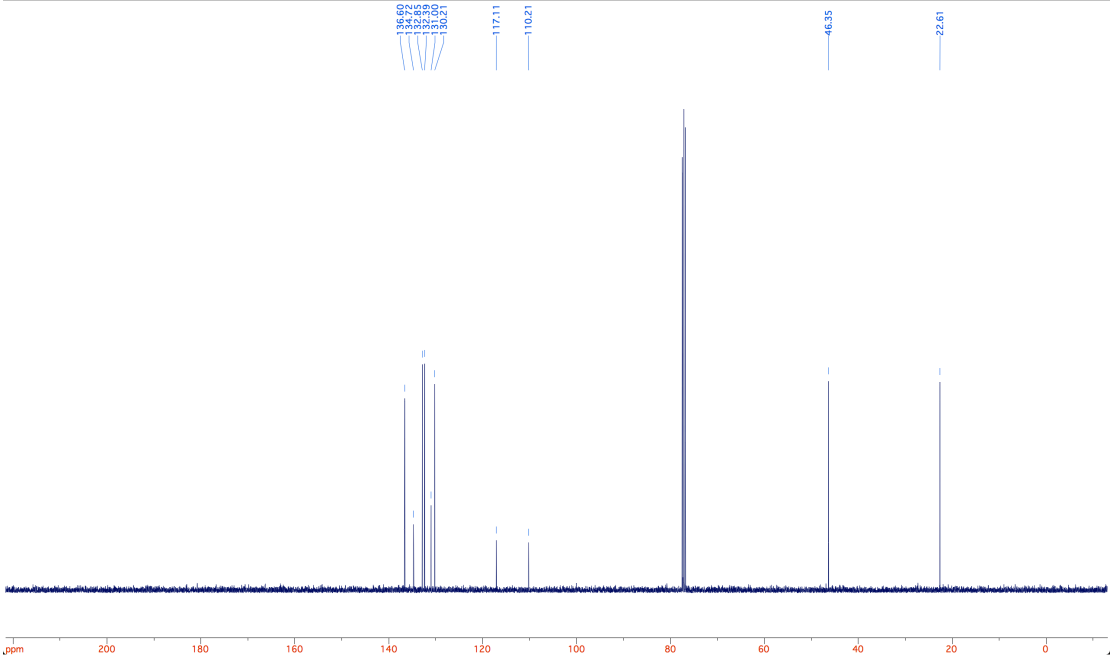

## 2-(Quinolin-3-yl)acetonitrile (36)

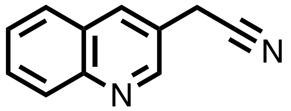


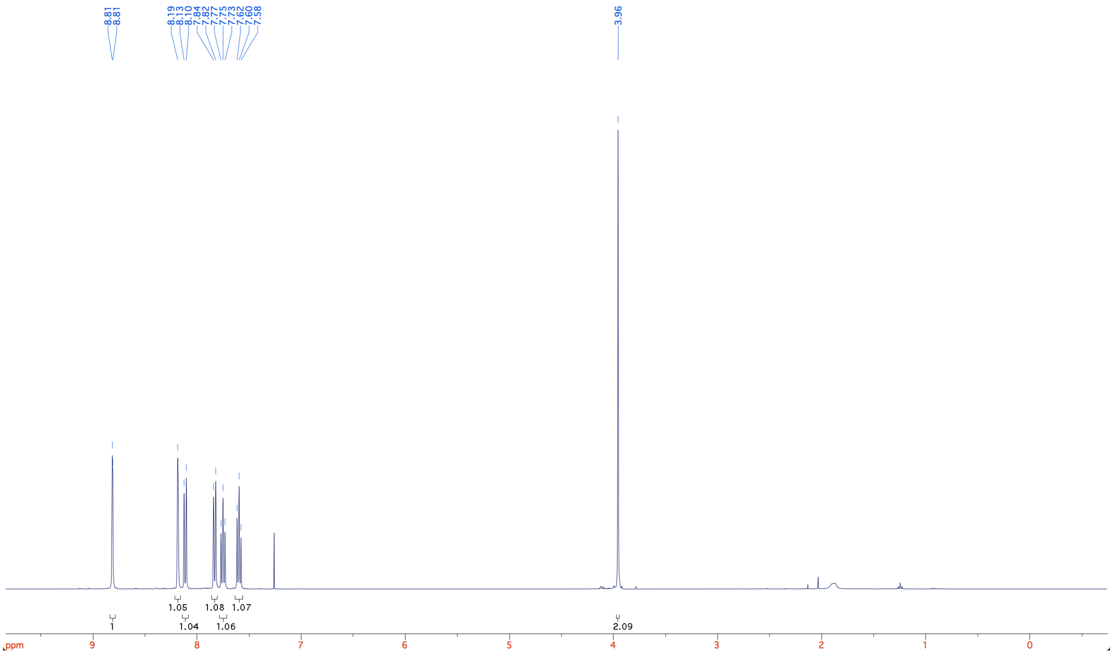


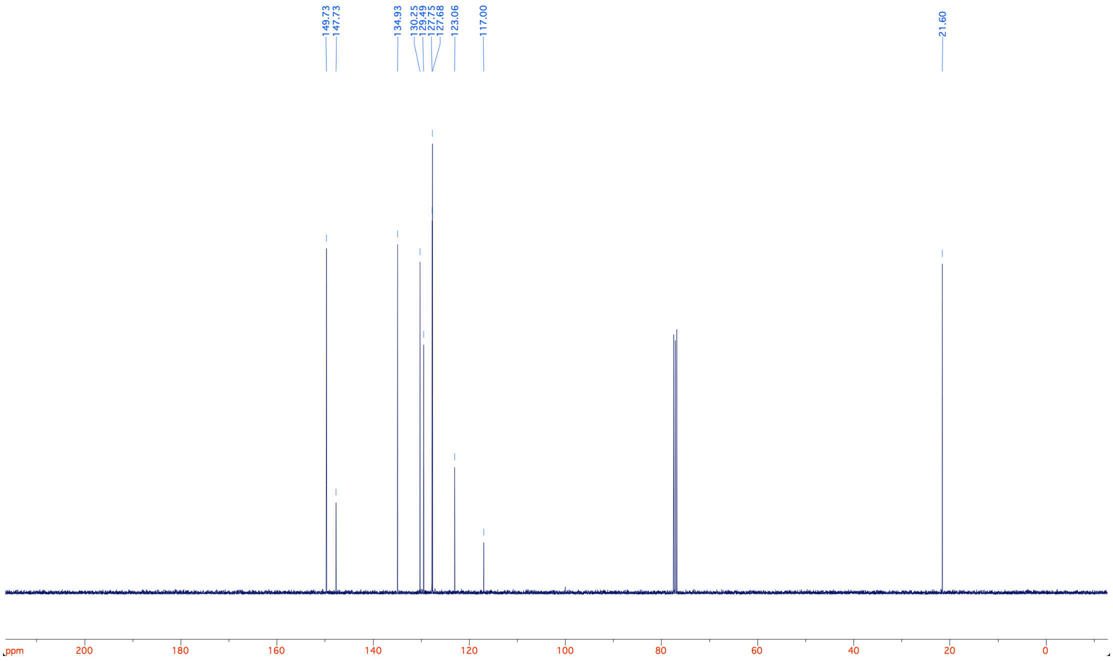

**2-(4-Nitrophenyl)acetonitrile (37)**

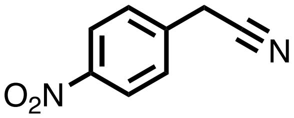


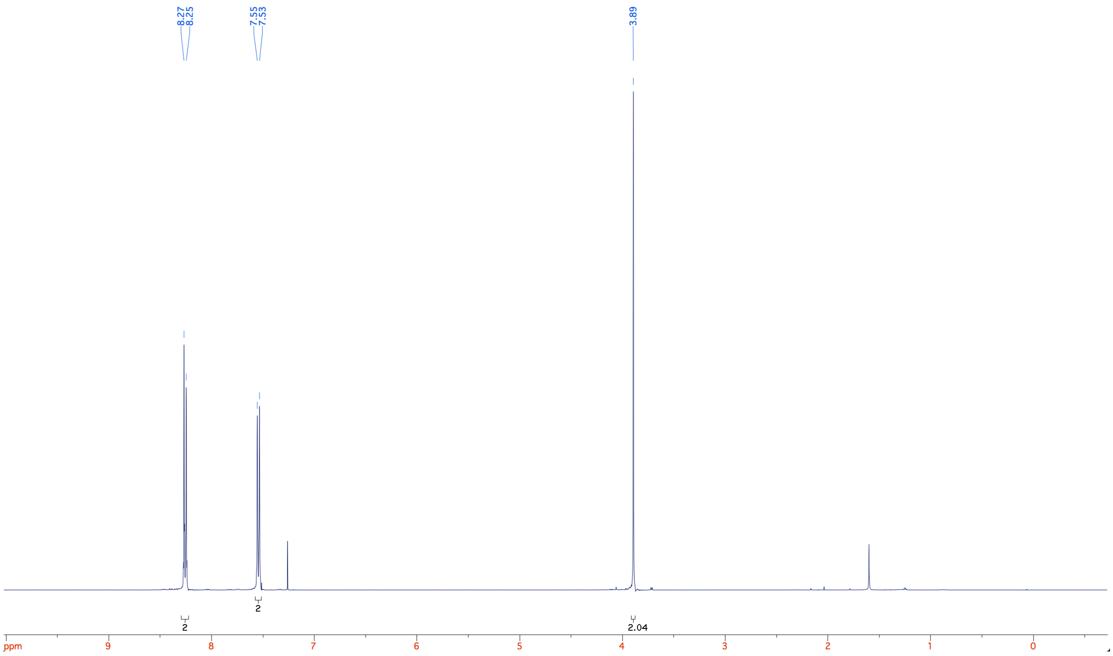


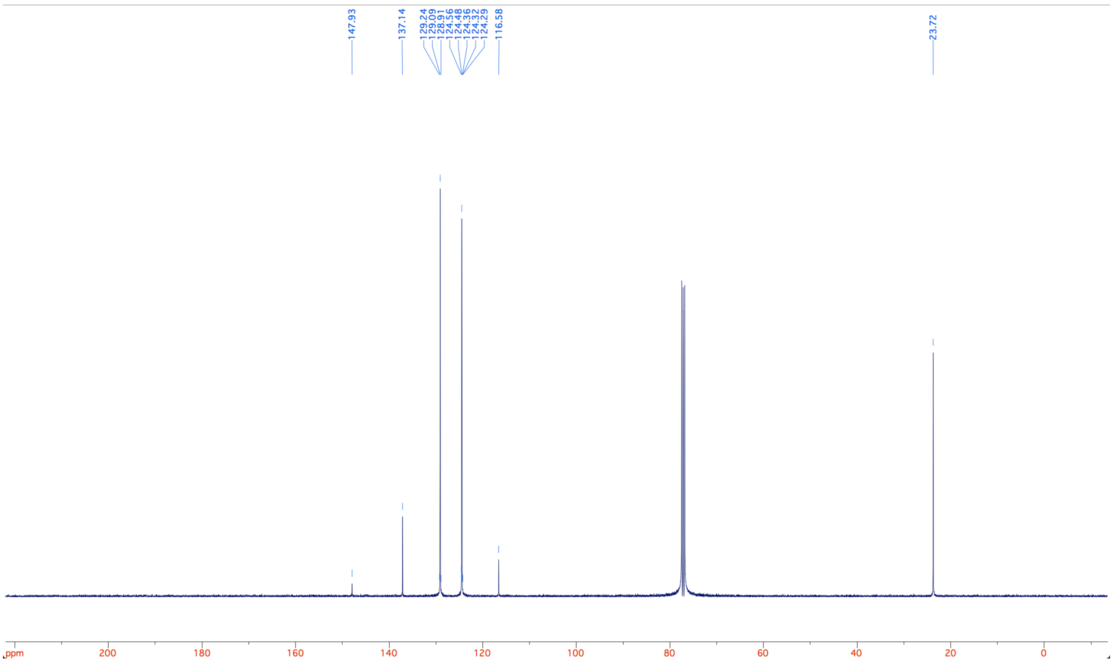

**2-(3-Bromophenyl)acetonitrile (38)**

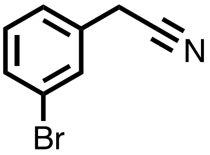


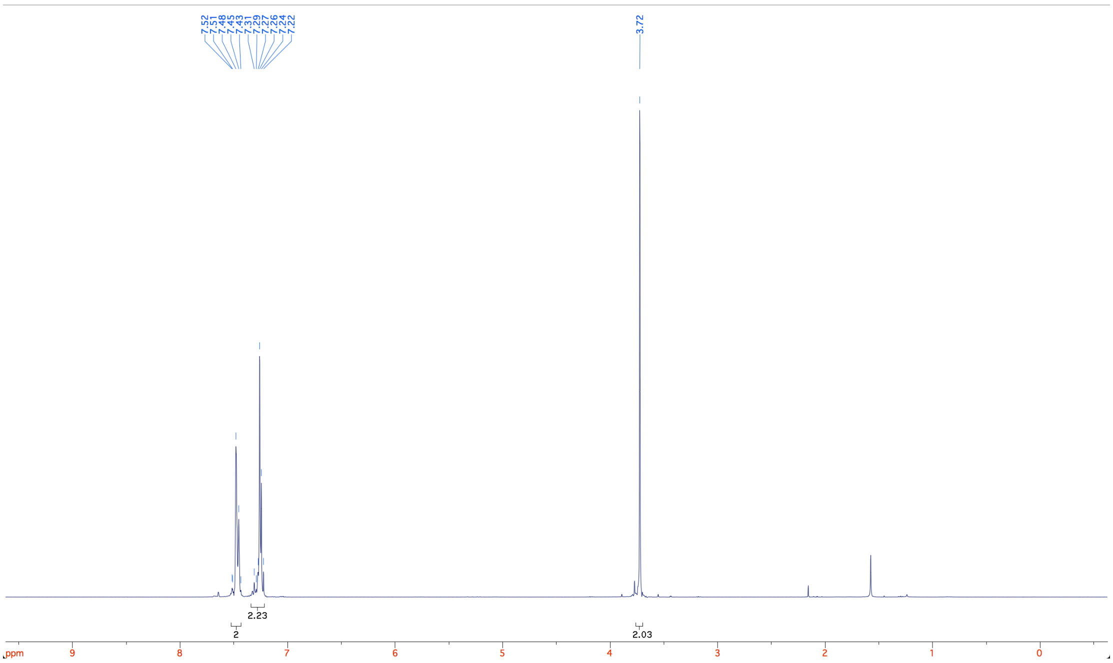


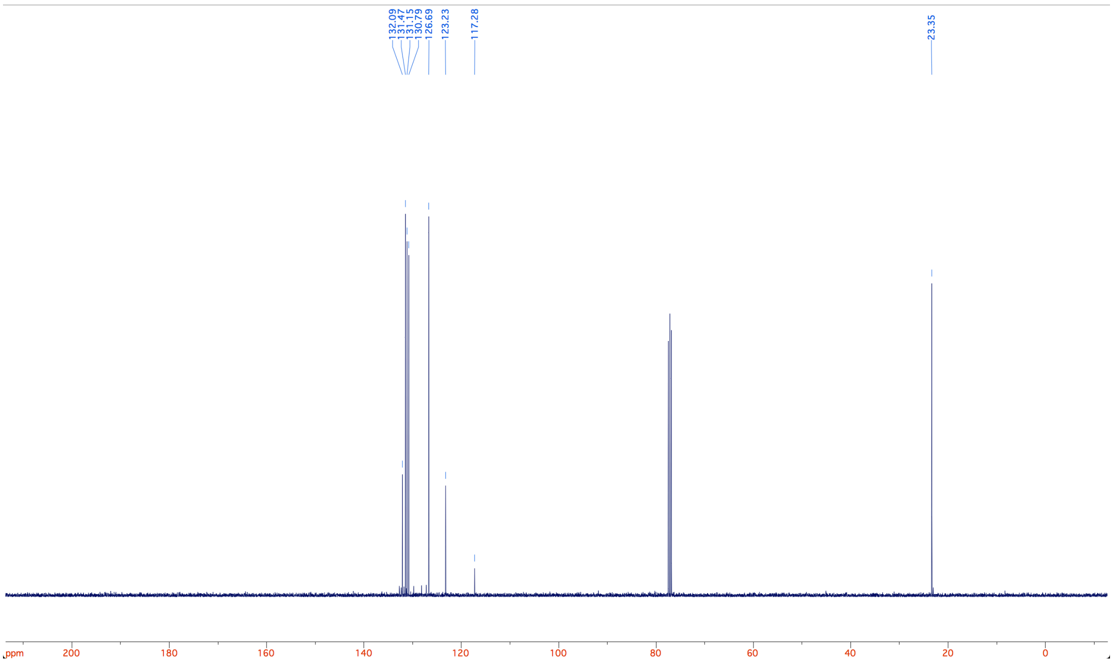

## Methyl 3-(cyanomethyl)benzoate (39)

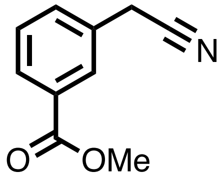


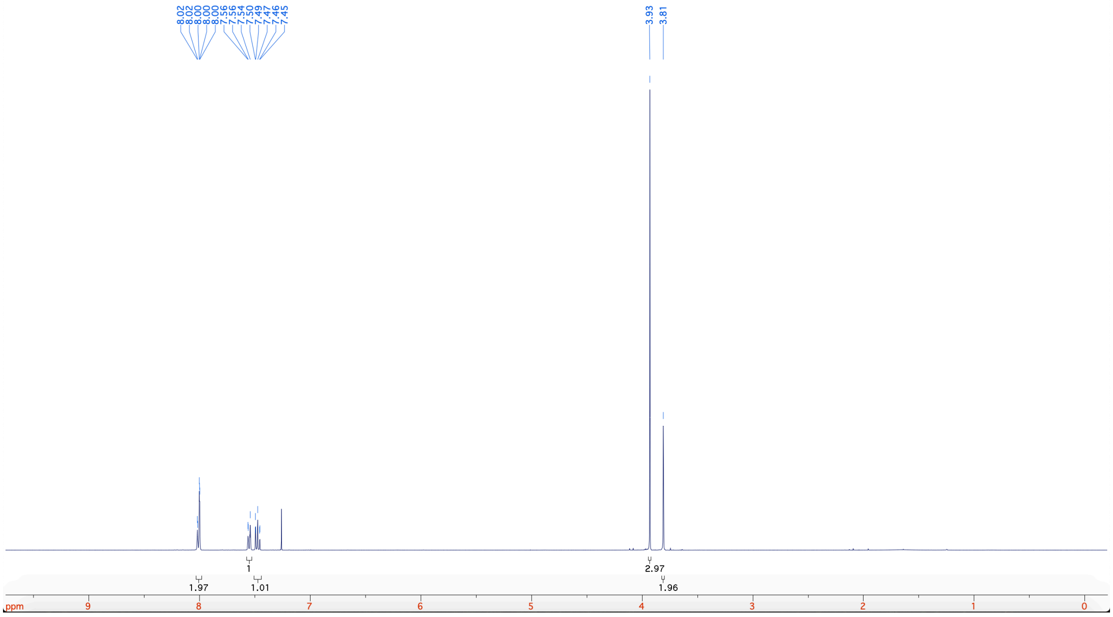


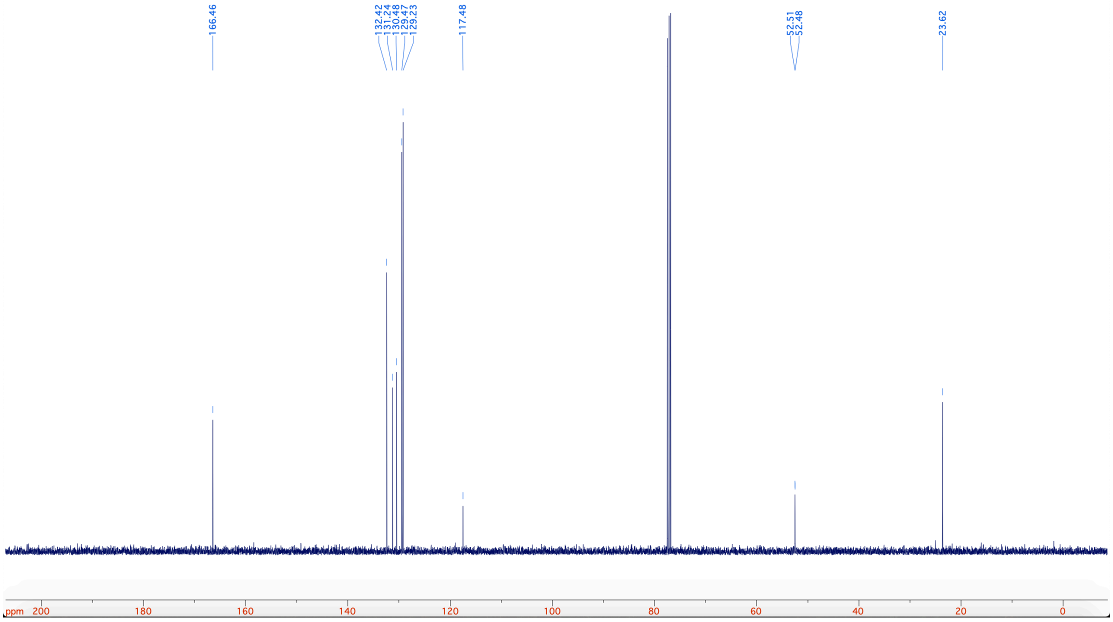

## 1,3-Dioxoisoindolin-2-yl 3-(4,5-diphenyloxazol-2-yl)propanoate

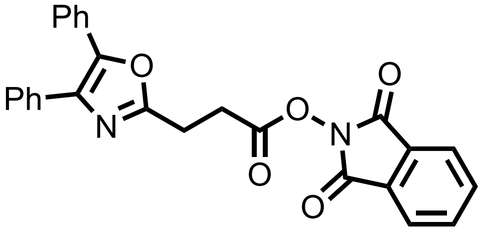


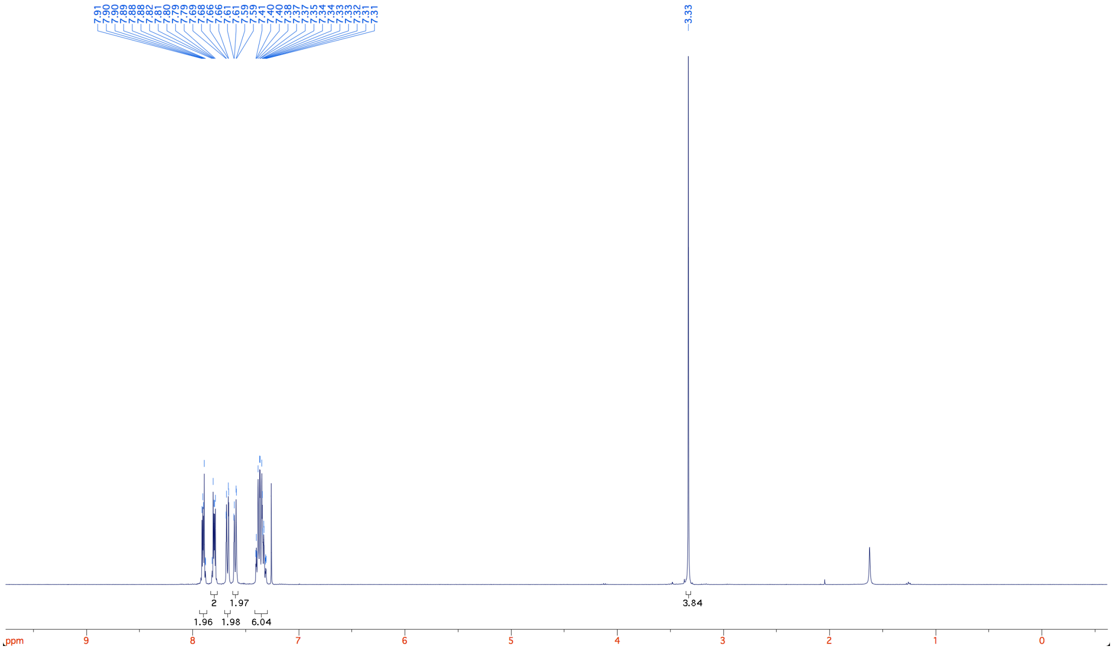


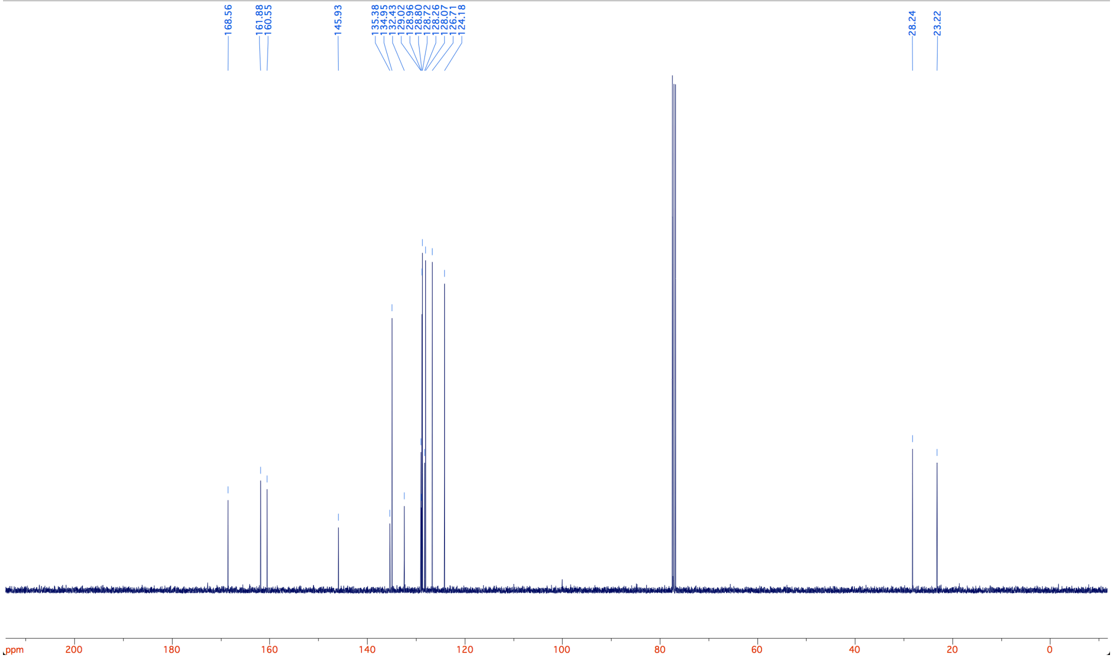

## 4-(4,5-Diphenyloxazol-2-yl)butanenitrile (41)

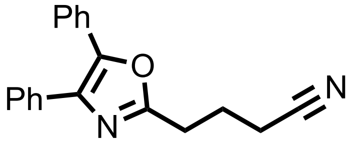


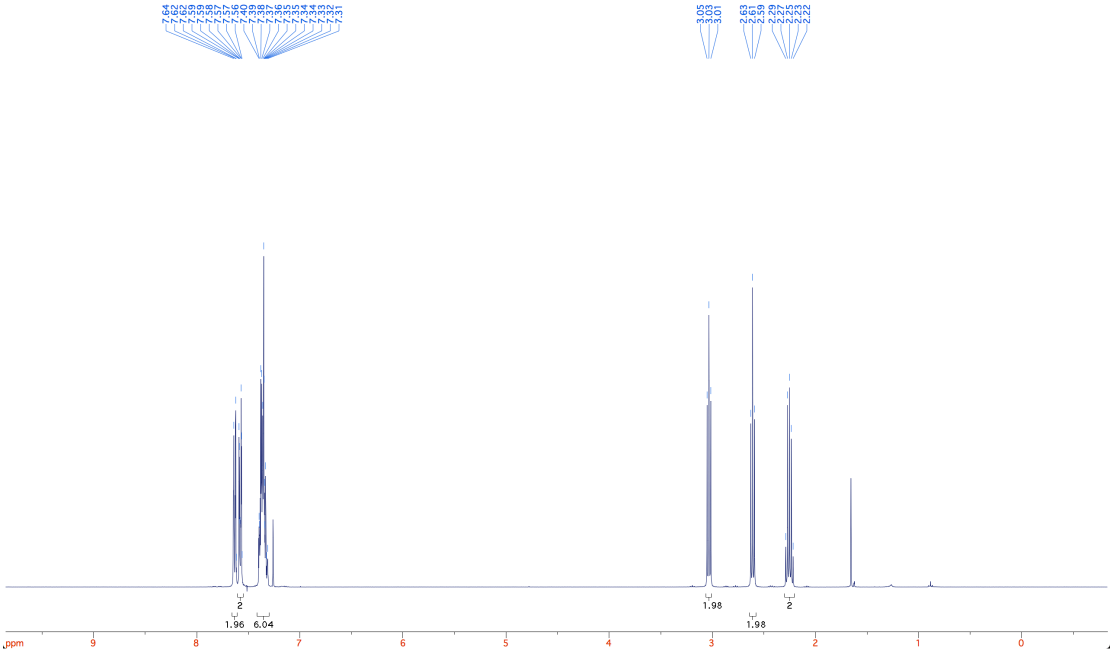


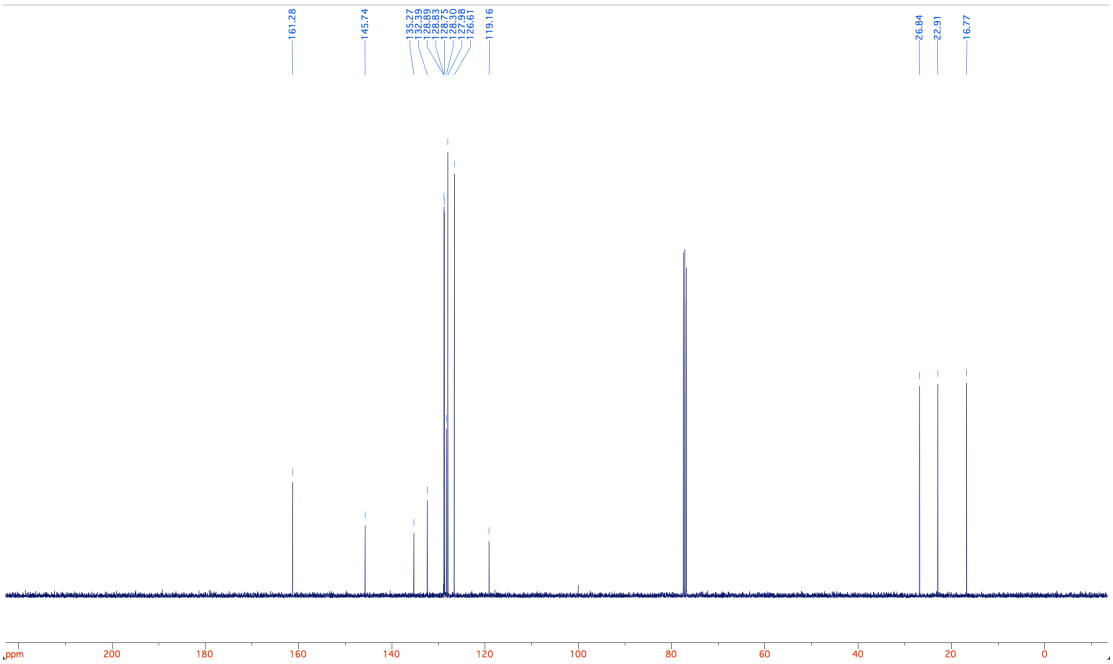

**6-Methylthiochroman-7-sulfonyl chloride 1,1-dioxide**

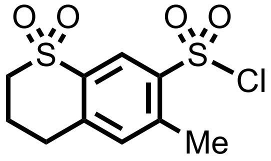

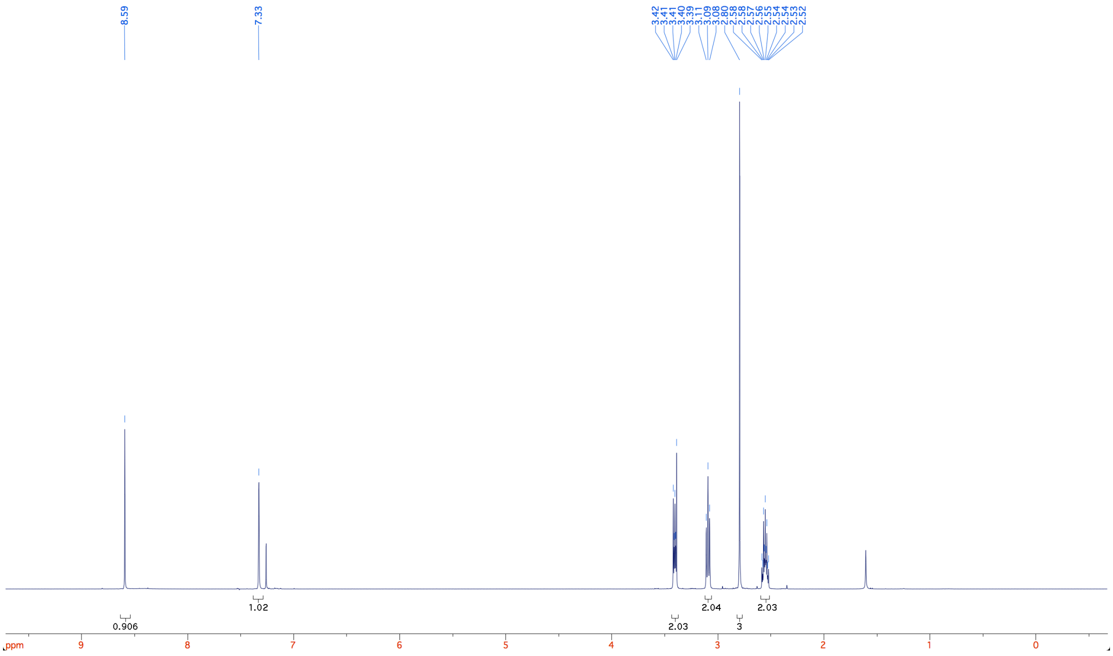


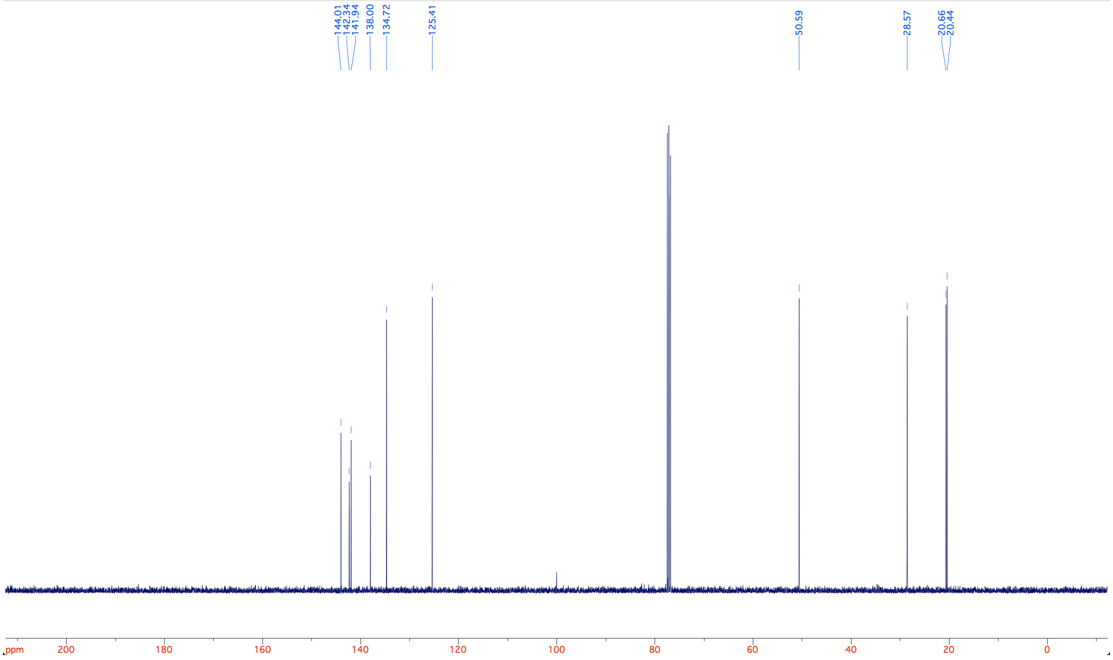

## 2-((6-Methyl-1,1-dioxidothiochroman-7-yl)sulfonyl)acetonitrile (43)

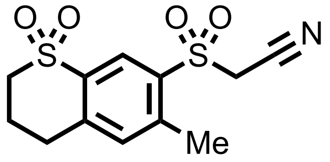


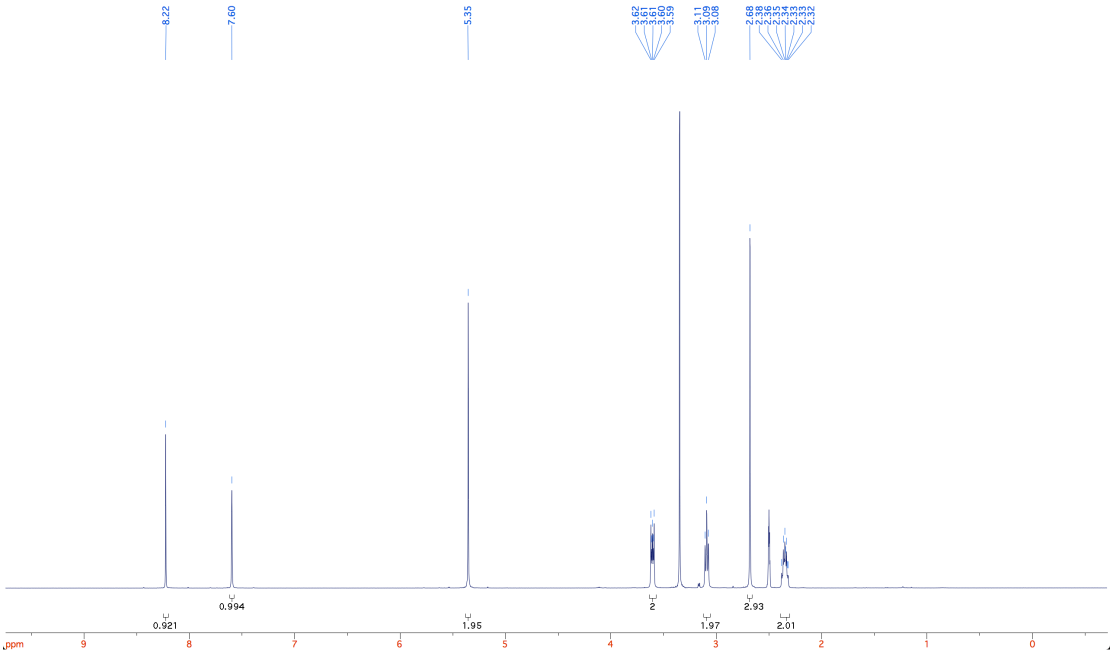


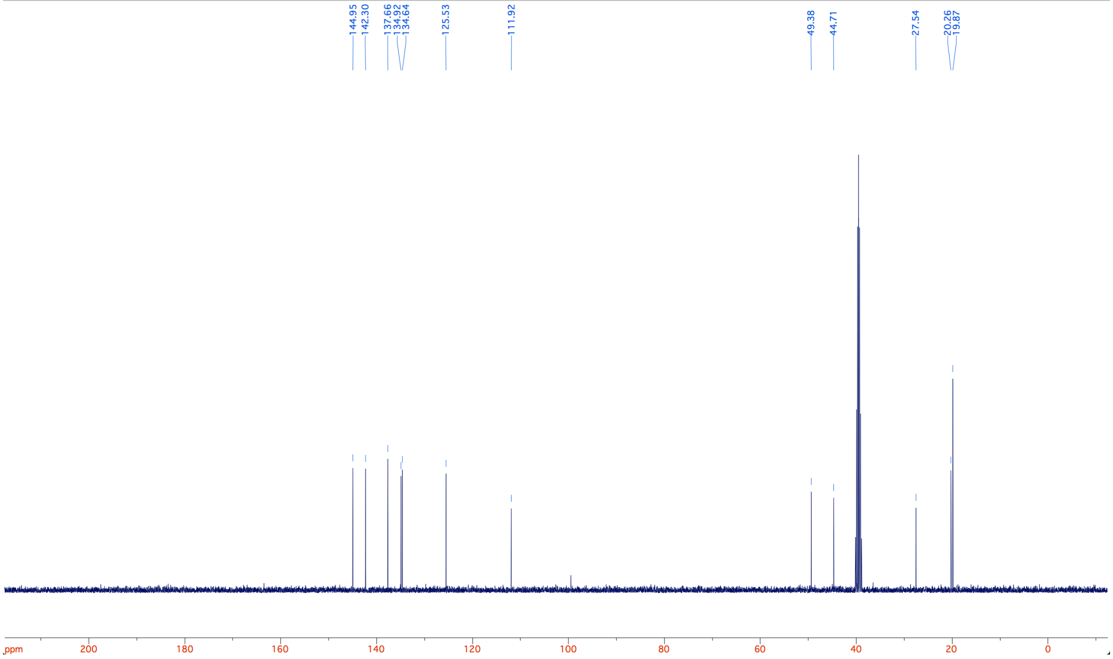

## 2-(4-(3-Ethyl-2,6-dioxopiperidin-3-yl)phenyl)acetonitrile (45)

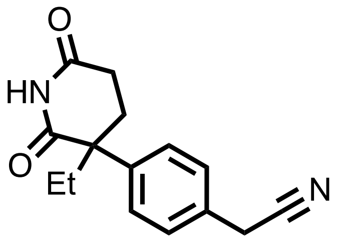


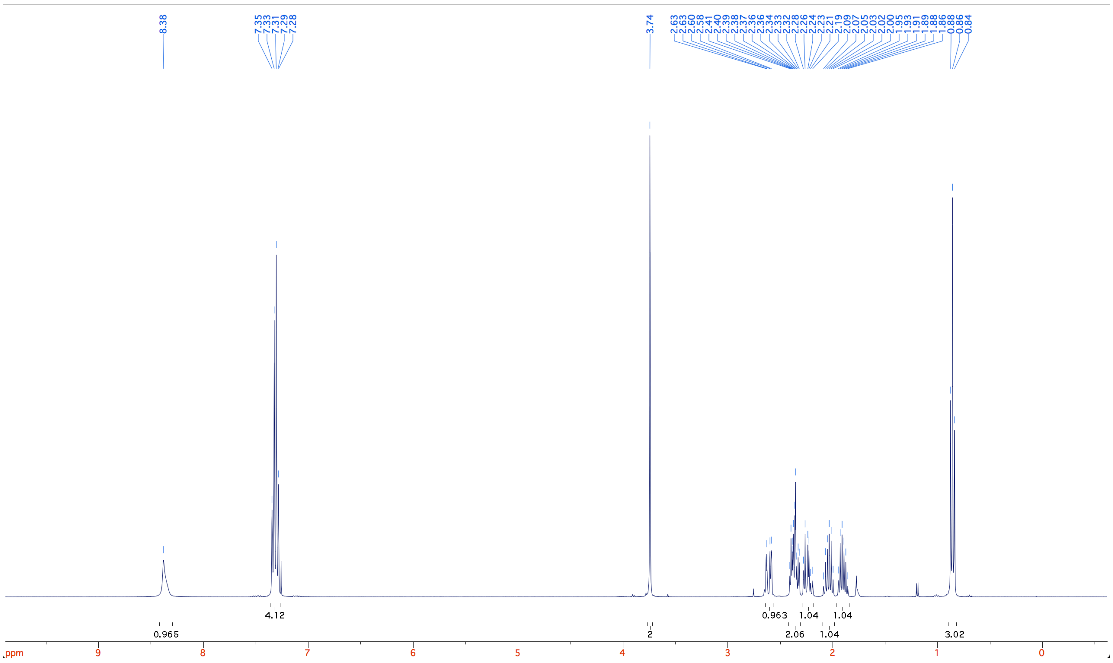


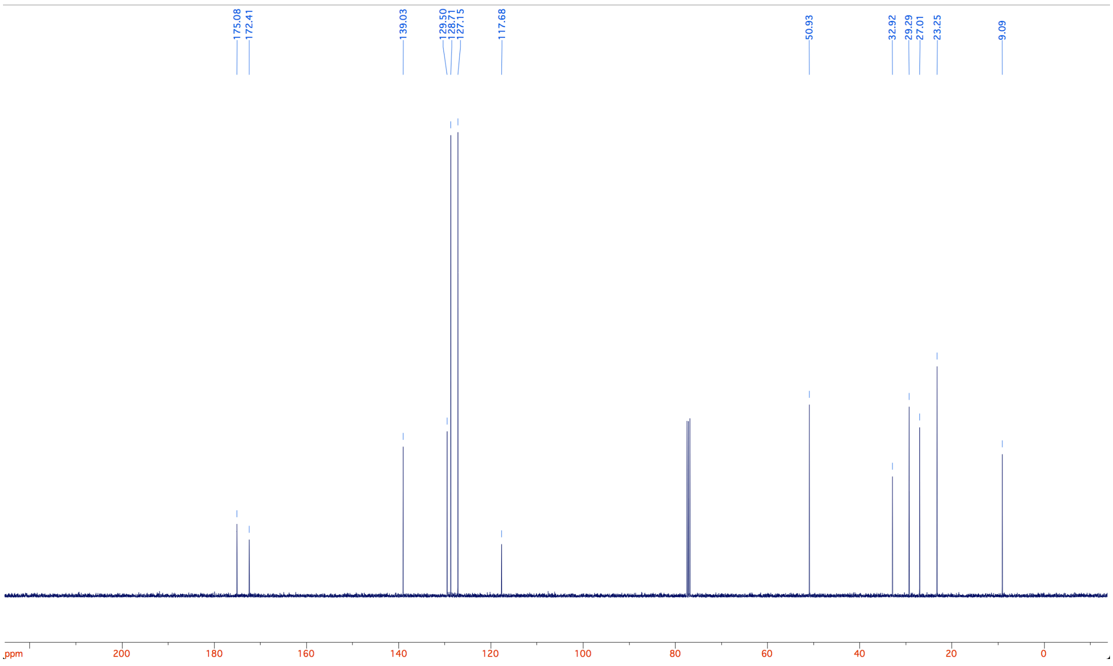

Supplement: Supplementary file 1 [file SC-010-C9SC01370A-s001.pdf]
